# New Indole-3-Propionic Acid and 5-Methoxy-Indole Carboxylic Acid Derived Hydrazone Hybrids as Multifunctional Neuroprotectors

**DOI:** 10.3390/antiox12040977

**Published:** 2023-04-21

**Authors:** Neda Anastassova, Denitsa Stefanova, Nadya Hristova-Avakumova, Irina Georgieva, Magdalena Kondeva-Burdina, Miroslav Rangelov, Nadezhda Todorova, Rumiana Tzoneva, Denitsa Yancheva

**Affiliations:** 1 Institute of Organic Chemistry with Centre of Phytochemistry, Bulgarian Academy of Sciences, Acad. G. Bonchev Str., Building 9, 1113 Sofia, Bulgaria; 2 Laboratory of Drug Metabolism and Drug Toxicity, Department of Pharmacology, Pharmacotherapy and Toxicology, Faculty of Pharmacy, Medical University-Sofia, 2 Dunav Str., 1000 Sofia, Bulgaria; 3 Department of Medical Physics and Biophysics, Faculty of Medicine, Medical University of Sofia, 2 Zdrave Str.,1431 Sofia, Bulgaria; 4 Laboratory of Transmembrane Signaling, Institute of Biophysics and Biomedical Engineering, Bulgarian Academy of Sciences, Acad. G. Bonchev Str., Block 21, 1113 Sofia, Bulgaria; 5 Institute of Biodiversity and Ecosystem Research, Bulgarian Academy of Sciences, 2 Gagarin Str., 1113 Sofia, Bulgaria

**Keywords:** neurodegenerative disorders, Parkinson’s disease, indoles, indole-3-propionic acid, hybrid compounds, neuroprotection, oxidative stress, metal chelation, BBB permeability, MAO-B

## Abstract

In light of the known neuroprotective properties of indole compounds and the promising potential of hydrazone derivatives, two series of aldehyde-heterocyclic hybrids combining those pharmacophores were synthesized as new multifunctional neuroprotectors. The obtained derivatives of indole-3-propionic acid (IPA) and 5-methoxy-indole carboxylic acid (5MICA) had good safety profiles: Hemolytic effects < 5% (200 μM) and IC_50_ > 150 µM were found in the majority of the SH-SY5Y and bEnd3 cell lines. The 2,3-dihydroxy, 2-hydroxy-4-methoxy, and syringaldehyde derivatives of 5MICA exhibited the strongest neuroprotection against H_2_O_2_-induced oxidative stress in SH-SY5Y cells and 6-OHDA-induced neurotoxicity in rat-brain synaptosomes. All the compounds suppressed the iron-induced lipid peroxidation. The hydroxyl derivatives were also the most active in terms of deoxyribose-degradation inhibition, whereas the 3,4-dihydroxy derivatives were able to decrease the superoxide-anion generation. Both series of compounds showed an increased inhibition of hMAO-B, with greater expression detected in the 5MICA hybrids. The in vitro BBB model with the bEnd3 cell line showed that some compounds increased the permeability of the endothelial monolayer while maintaining the tight junctions. The combined results demonstrated that the derivatives of IPA and 5MICA showed strong neuroprotective, antioxidant, MAO-B inhibitory activity and could be considered as prospective multifunctional compounds for the treatment of neurodegenerative disorders.

## 1. Introduction

Parkinson’s disease (PD) is a neurodegenerative disorder with a complex multifactorial etiology involving a variety of pathological processes, including protein-misfolding and the generation of insoluble fibrils; the impairment of mitochondrial functions; the dysregulation of the transition metal-ion homeostasis; the overproduction of reactive oxygen species; and neuroinflammation, leading to dopaminergic neuronal death [1,2,3]. There is growing evidence that iron metabolism is disrupted in PD, and that the resulting generation of reactive oxygen species (ROS), in combination with relatively low antioxidant levels and reduced repair capacity (non-replicating neuronal cells), make the brain tissue vulnerable to oxidative stress injury [4,5,6]. The exacerbation of ROS generation results in the alterations of oxidative stress biomarkers, especially those associated with lipid peroxidation (LP). HNE (4-hydroxynonenal) was elevated in the postmortem brain tissue and the cerebrospinal fluid in PD patients [7]. The plasma levels of MDA and isoprostanes also increased, exhibiting toxic effects and contributing to neurodegeneration [7]. HNE and Nε-(carboxymethyl)lysine have been localized in Lewy bodies. Furthermore, the HNE modification of α-synuclein was proven to result in oligomerization, and the generated HNE-modified oligomers were described as potentially toxic and contributing to neuronal death [8]. The aforementioned results have suggested that the design of new multi-potent neuroprotectors, targeting not only the dopaminergic pathway but also ROS productions and Fe-induced damage of biologically important molecules (especially LP), is a promising strategy in drug development. Therefore, different ligands were designed with multifunctional activity to interact with various pathological hallmarks [9,10].

**Scheme 1:**
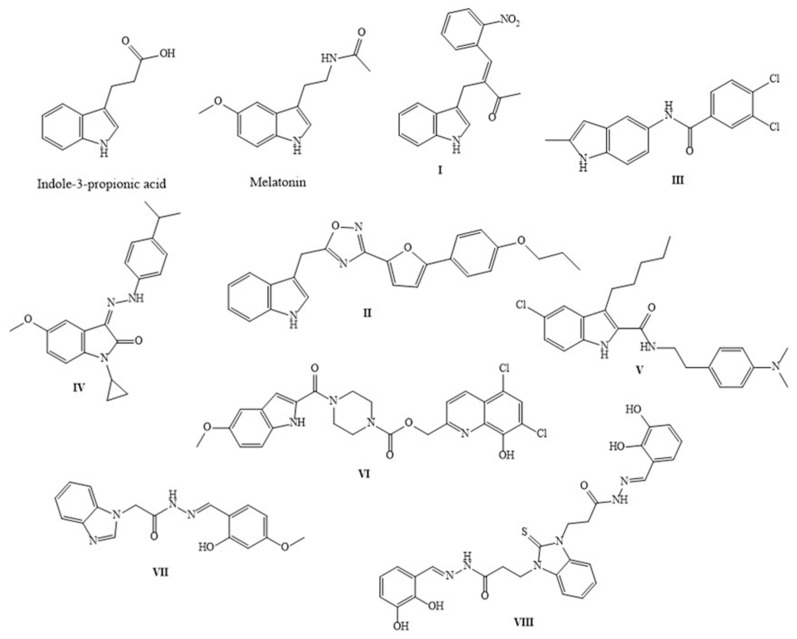
Chemical structures of indole derivatives with versatile activity for the treatment of neurological conditions.

Indole-3-propionic acid (IPA) was considered a key compound for developing treatments for neurodegenerative disorders and other neurological diseases [11,12,13,14]. The lack of pro-oxidant effects was, perhaps, the most important feature of IPA, which was shared only by melatonin [15]. Melatonin and IPA (Scheme 1) were not cytotoxic and, in contrast to synthetic antioxidants, could be found in the body under physiological conditions. The free radical-scavenging properties of IPA surpassed those of melatonin. The latter was extensively studied by this team in a novel pin+icvAβ_1-42_ Alzheimer animal model, and its role as a direct free radical scavenger was unequivocally demonstrated in the prevention of oxidative stress and neurodegenerative features induced by that model [16]. IPA was able to prevent ROS damage and cell death caused by the addition of Aβ-peptide on SK-N-SH human neuroblastoma cells and primary rat hippocampal neurons [17]. IPA also demonstrated full protection against the death of primary neurons and SK-N-SH cells exposed to Aβ-mediated damage and DDTC-induced lipid peroxidation in PC12 cells [15].

Another endogenous indole derivative, indole propionamide, has been shown to prolong the rotifer lifespan and recover the mitochondrial metabolic function in rodents by decreasing the generation of free radicals [18]. NC009-1 (**I**, Scheme 1) demonstrated neuroprotective effects by modulating inflammatory and anti-oxidative pathways in mouse models of PD [19]. A series of indole-based 1,2,4-oxadiazoles were evaluated as multifunctional neuroprotective agents, of which two compounds containing a 4-ethoxyphenyl and 4-propoxyphenyl (**II**) residues demonstrated a combined activity against Aβ_25–35_-, H_2_O_2_-, and oxygen–glucose-deprivation-induced neurotoxicity in SH-SY5Y cells [20]. The indole nucleus may also serve as a suitable scaffold for the creation of MAO inhibitors. Series of indole derivatives were found to be selective inhibitors of MAO-B, with potencies in the sub-micromolar range, of which the 3,4-dichloro derivative (**III**) had the most potent activity [21]. Indolin-2-one 3-arylhydrazones were outlined as privileged multi-target structures for AD therapy, among which the N1-cyclopropyl derivative (**IV**) showed strong cytoprotective effects against both Aβ_42_ oligomer toxicity and oxidative stress [22].

The indole-2-carboxamide is another indole-based scaffold of current interest that has exhibited broad biological activities, including anticancer, antiviral, and antioxidant, especially with strong radical-scavenging properties against superoxide radicals [23]. Within this class of compounds, a potent allosteric modulator of the cannabinoid receptor I (**V**) was discovered [24]. A hybrid compound based on 8-hydroxyquinoline-5-methoxy indole-2-carboxamide (**VI**) afforded strong inhibition against Cu^2+^-induced and Zn^2+^-induced Aβ_1–42_ aggregation assays and was able to chelate Cu Zn^2+^ and Fe^2+^ [25].

In the search for new treatments for neurological diseases, hydrazones have emerged as a promising class of compounds with versatile biological activities. N-acylhydrazones showed strong iron-chelating properties and, therefore, were originally suggested as drug candidates for the treatment of iron-overload diseases [26]. In particular, they were able to moderately bind biometals, and this affinity and specificity could be modified by the appropriate selection of the aldehyde precursor and substituents [27]. For example, in the well-studied N-acylhydrazone INHHQ, it has been shown that, in healthy Wistar rats, N-acylhydrazone was capable of crossing the blood–brain barrier. Moreover, it did not affect the GSH and biometal levels under normal homeostasis in different tissues such as brain, liver, kidneys, and heart, whereas in an in vitro threefold excess α-Syn, it efficiently competed for the binding of copper [28]. Moreover, hydrazone derivatives have been synthesized as dual AChE and MAO-B inhibitors for the treatment of PD [29,30].

Our earlier studies on broad series of benzimidazole-based arylhydrazones [31,32,33] outlined the molecular structures bearing the pharmacophores with the most potent multi-target activities in terms of neuroprotection, radical scavenging, and MAO-B inhibition. In particular, the leading structure outlined within a series of benzimidazole hybrids with MAO-B-inhibiting activity was the derivative containing a 2-hydroxy-4-methoxy vanilloid residue (**VII**) [32]. Our latest study on 17 *N, N*′-disubstituted benzimidazole-arylhydrazone hybrids [31] outlined some prospective compounds containing hydroxyl and vanilloid fragments, among which the catecholic derivative containing 2,3-dihydroxy moiety (**VIII**) had excellent potential due to its good safety profile, its neuroprotection of SH-SY5Y cells, and its isolation of rat-brain synaptosomes under conditions induced by toxic agents of oxidative stress. It was able to restore the cellular and synaptosomal viability, up to 80%, and showed a more potent MAO-B inhibitory effect, as compared to the rest of the series and the reference melatonin and rasagiline. Moreover, it exhibited the strongest capability to decrease lipid peroxidation in iron-induced lecithin oxidative damage and the iron/deoxyribose system, as well as a scavenging capability against superoxide radicals. Our studies unequivocally demonstrated that hydroxy and methoxy substitution in the arylhydrazone fragment improved the neuroprotective properties. Currently, based on the well-known biological properties of indoles, and more precisely, of melatonin, IPA, and 5-methoxy-indole carboxylic acid (5MICA) derivatives, it was considered of interest to widen our research with the synthesis of new selected series based on these scaffolds that possessed the already proven functionalities. The current pilot study aimed to investigate their potential in the treatment of neurodegenerative disorders by establishing their safety profiles and evaluating their potential as iron-chelators with neuroprotective, antioxidant, and MAO-B inhibiting properties, as well as to elucidate their capabilities to cross the BBB.

## 2. Materials and Methods

### 2.1. Chemistry

A Büchi B-540 instrument was used to determine the melting points (mp) of the compounds. The corresponding values were reported uncorrected. ATR-IR spectroscopy measurements were carried out on a Bruker Invenio R instrument with a diamond ATR module. The spectra were recorded with 64 scans at a resolution of 2 cm^−1^. NMR spectra (^1^H and ^13^C) were measured on a Bruker Neo 600 MHz NMR spectrometer. The spectra were referred to as the solvent signal. Chemical shifts were reported in ppm and coupling constants in Hz. The progress of the reactions and purity of the products were established by thin-layer chromatography. Merck pre-coated plates (silica gel 60 F254 at 0.25 mm) were used, and the locations were visualized by UV light (254 nm). Melting points and spectral data were provided only for the novel target compounds **3a–f** and **5a–f**.

### 2.2. Synthesis of Compounds ***1***, ***2*** and ***4***

To obtain the methyl ester of indole-3-propionic acid (**1**), we utilized a method consisting of refluxing the carboxylic acid (0.005 mol) with thionyl chloride (4 equiv.) and methanol (2 mL/mmol carboxylic acid). Hydrazine hydrate (0.06 mol) and 0.01 mol of methyl ester of indole-3-propionic acid **1** (Series I) or methyl ester of 5-methoxyindole-2-carboxylic acid (Series II) were refluxed in ethanol for 2 h. After the reaction was complete, the mixture was cooled, and the obtained crystals were filtered and recrystallized with ethanol. The progress of the reaction was monitored using TLC (ethylacetate:hexane = 4:1). The precipitated product was recrystallized with ethanol, and the structure was confirmed by ^1^H and ^13^C NMR spectroscopy (Figures S1, S11 and S12, Appendix A).

### 2.3. General Procedure for the Synthesis of Compounds ***3a–f*** and ***5a–f***

To a solution of the hydrazide 2 (0.5 mmol) in absolute ethanol were added variously substituted hydroxy and methoxy benzaldehydes (1.0 equiv.), and the reaction was refluxed for 1–6 h, depending on the substituents. The progress of the reaction was monitored using TLC (benzene:methanol = 4:1). After completion and concentration under reduced pressure, the precipitated product was filtered and washed with cool ethanol. The compounds were recrystallized with ethanol, and the purity was confirmed by TLC, IR, and ^1^H NMR spectroscopy. The ATR-IR and NMR spectra are provided in the Supplementary Materials.

#### 2.3.1. N′-(2,3-Dihydroxybenzylidene)-3-(1H-indol-3-yl)propanehydrazide (**3a**)

Yield 81%, Mp 204–205 °C, IR (ν_max_/cm^−1^) 3438 (νO-H); 3268 (νN-H); 2961, 2926 (ν_as_CH_2_); 2951 (ν_s_CH_3_); 1650 (νC=O) amide I; 1608 (νC=N); 1564 (δN-H); 1259 (νC-O-C). ^1^H NMR (600 MHz, DMSO-*d*_6_) δ, ppm: δ 11.65 (s, 1H, NH), 11.04, 11.26 (s, 1H, OH), 10.77 (s, 1H, NH), 9.49, 9.24 (d, *J* = 5.9 Hz; s, 1H, OH), 8.25, 8.28 (s, 1H, CH), 7.55–7.57 (d, *J* = 7.7 Hz, 1H, Ar-H), 7.33–7.35 (d, *J* = 8.0 Hz, 1H, Ar-H), 7.12–7.13 (m, 1H, Ar-H), 7.03–7.09 (m, 1H, Ar-H), 6.96–7.02 (m, 1H, Ar-J), 6.78–6.84 (m, 1H, Ar-H), 6.65–6.74 (m, 1H, Ar-H), 2.93–3.04 (m, 2H, CH2), 2.59–2.62 (m, 2H, CH2) ^13^C NMR (151 MHz, DMSO-*d*_6_) δ, ppm: 174.03, 168.78, 147.74, 146.35, 145.99, 145.94, 145.57, 142.51, 136.71, 127.47, 127.41, 122.73, 121.48, 120.72, 120.50, 119.80, 119.61, 119.14, 118.79, 118.74, 117.99, 117.71, 117.04, 114.23, 113.91, 111.86, 35.33, 33.55, 21.07, 20.34, 18.95.

#### 2.3.2. N′-(3,4-Dihydroxybenzylidene)-3-(1H-indol-3-yl)propanehydrazide (**3b**)

Yield 75%, IR (ν_max_/cm^−1^) 3395 (νO-H); 3274 (νN-H); 2967, 2919 (ν_as_CH_2_); 2850 (ν_s_CH_2_); 1641 (νC=O) amide I; 1604 (ν C=N); 1590 (δ N-H); 1282 (νC-O-C). ^1^H NMR (400 MHz, DMSO-*d*_6_) δ 11.01 (s, 1H, NH), 9.11 (s, 1H, OH), 7.55, 7.52 (s, 1H, CH), 7.26–7.31 (m, 2H, Ar-H), 7.07–7.11 (m, 2H, Ar-H), 6.89–7.00 (m, 2H, Ar-H), 6.68–6.83 (m, 1H, Ar-H), 3.15–3.19 (m, 2H, CH_2_), 2.39–2.43 (m, 2H, CH_2_). ^13^C NMR (151 MHz, DMSO-*d*_6_) δ, ppm: 171.82, 156.91, 140.40, 139.40, 137.23, 128.42, 127.28, 125.54, 123.77, 122.75, 119.32, 118.81, 111.78, 111.68, 34.88, 21.26, 18.96.

#### 2.3.3. N′-(2,4-Dihydroxybenzylidene)-3-(1H-indol-3-yl)propanehydrazide (**3c**)

Yield 87%, Mp 239–240 °C, IR (ν_max_/cm^−1^) 3377, 325 8 (νO-H); 3227 (νN-H); 2951, 2926 (ν_as_CH_2_); 2856 (ν_s_CH_2_); 1677 (νC=O) amide I; 1632 (νC=N); 1562 (δN-H); 1220 (νC-O-C). ^1^H NMR (400 MHz, DMSO-*d*_6_) δ, ppm: 11.41, 11.06 (s, 1H, OH), 11.36, 10.14 (s, 1H, NH), 10.77 (s, 1H, NH), 9.90, 9.77 (s, 1H, OH), 8.13, 8.19 (s, 1H, CH), 7.53–7.56 (d, *J* = 7.6 Hz, 0H), 7.23–7.38 (m,2H, Ar-H), 7.12–7.13 (m, 1H, Ar-H), 6.96–7.09 (m, 2H, Ar-H), 6.28–6.34 (m, 2H, Ar-H), 2.89–3.02 (m, 3H, CH2), 2.54–2.58 (m, 1H, CH2). ^13^C NMR (151 MHz, DMSO-*d*_6_) δ, ppm: 168.23, 160.96, 159.76, 158.50, 147.83, 142.53, 136.72, 131.66, 127.43, 122.72, 121.41, 118.78, 118.66, 113.96, 111.99, 111.83, 110.92, 108.24, 108.02, 103.06, 35.35, 33.51, 21.17, 19.02.

#### 2.3.4. N′-(4-Hydroxy-3-methoxybenzylidene)-3-(1H-indol-3-yl)propanehydrazide (**3d**)

Yield 85%, Mp 157–158 °C, IR (ν_max_/cm^−1^) 3373 (νO-H); 3197 (νN-H); 2961 (ν_as_CH_3_); 2921 (ν_as_CH_2_); 2852 (ν_s_CH_2_); 1650 (νC=O) amide I; 1600 (ν C=N); 1588 (δ N-H); 1270 (ν C-O-C). ^1^H NMR (600 MHz, DMSO-*d*_6_) δ, ppm: 11.19, 11.12 (s, 1H, NH), 10.79 (s, 1H, NH), 9.47 (s, 1H, OH), 8.01, 7.87 (s, 1H, CH), 7.54–7.57 (s, 1H, Ar-H), 7.32–7.33 (m, 1H, Ar-H), 7.20–7.24 (m, 1H, Ar-H), 7.12–7.15 (m, 1H-Ar-H), 7.01–7.07 (m, 2H, Ar-H), 6.94–6.99 (m, 1H, Ar-H), 3.76, 3.81 (s, 3H. OCH3), 2.96–3.01 (m, 3H, CH2), 2.53–2.56 (m, 1H, CH2). ^13^C NMR (151 MHz, DMSO-*d*_6_) δ, ppm: δ 175.35, 169.90, 149.00, 148.73, 148.40, 148.29, 147.76, 144.68, 136.62, 136.55, 127.30, 126.16, 126.02, 122.83, 122.69, 121.66, 118.97, 118.87, 118.77, 118.63, 115.93, 115.67, 114.16, 113.85, 111.88, 109.44, 55.99, 55.88, 35.23, 33.39, 20.99, 20.77.

#### 2.3.5. N′-(4-Hydroxy-2-methoxybenzylidene)-3-(1H-indol-3-yl)propanehydrazide (**3e**)

Yield 78%, Mp 152–153 °C, IR (ν_max_/cm^−1^) 3290 (νO-H); 3200 (νN-H); 2986 (ν_as_CH_3_); 2940 (ν_as_CH_2_); 2845 (ν_s_CH_2_); 1624 (νC=O) amide I; 1608 (νC=N); 1566 (δN-H); 1283 (νC-O-C). ^1^H NMR (600 MHz, DMSO-*d*_6_) δ 11.52 (s, 1H, NH), 11.15, 10.79 (s, 1H, NH), 8.87 (s, 1H, CH), 8.17, 8.24 (s, 1H, OH), 7.48–7.55 (m, 2H-Ar-H), 7.32–7.38 (m, 1H-Ar-H) 6.97-7.13 (m, 2H, Ar-H), 6.56–6.58 (dd, *J* = 8.6, 2.4 Hz, 1H), 6.42–6.53 (m, 2H, Ar-H), 3.80 (s, 3H, OCH3), 2.91–3.01 (m, 2H, CH2), 2.56–2.59 (m, 2H, CH2). ^13^C NMR (151 MHz, DMSO-*d*_6_) δ, ppm: 174.58, 169.48, 162.50, 159.41, 158.43, 148.25, 136.56, 131.74, 127.27, 122.71, 121.67, 118.97, 118.76, 118.63, 114.04, 113.79, 112.82, 111.93, 111.89, 107.02, 101.51, 101.40, 56.80, 55.73, 35.15, 20.95, 20.34, 18.42.

#### 2.3.6. N′-(4-Hydroxy-3,5-dimethoxybenzylidene)-3-(1H-indol-3-yl)propanehydrazide (**3f**)

Yield 80%, Mp 134–135 °C, IR (ν_max_/cm^−1^) 3396 (νO-H); 3188 (νN-H); 2965 (ν_as_CH_3_); 2924 (ν_as_CH_2_); 2850 (ν_s_CH_2_); 1657 (νC=O) amide I; 1619 (νC=N); 1587 (δN-H); 1285, 1107 (νC-O-C); ^1^H NMR (600 MHz, DMSO-*d*_6_) δ 11.18, 11.23 (s, 1H, NH), 10.79 (s, 1H, NH), 8.85 (s, 1H, OH), 8.01, 7.87 (s, 1H, CH), 7.55–7.57 (dd, *J* = 7.9, 4.2 Hz, 1H, Ar-H), 7.31–7.34 (m, 1H, Ar-H), 7.12–7.16 (m, 2H, Ar-H), 7.04–7.08 (m, 1H, Ar-H), 6.91–6.99 (m, 3H, Ar-H), 3.76, 3.80 (s, 3H, OCH3), 2.97–3.01 (m, 3H, CH3), 2.54–2.61 (m, 1H, CH2). ^13^C NMR (151 MHz, DMSO-*d*_6_) δ, ppm: 169.92, 161.87, 148.43, 148.35, 147.84, 139.07, 137.85, 136.55, 127.30, 124.87, 124.44, 122.69, 121.65, 118.97, 118.77, 113.84, 111.87, 106.10, 105.04, 104.52, 56.79, 35.20, 20.98.

#### 2.3.7. N′-(2,3-Dihydroxybenzylidene)-5-methoxy-1H-indole-2-carbohydrazide (**5a**)

Yield 88%, Mp 262–263 °C, IR (ν_max_/cm^−1^) 3315 (νO-H); 3259 (νN-H); 2970, 2938 (ν_as_CH_2_); 2836 (ν_s_CH_3_); 1637 (νC=O) amide I; 1608 (νC=N); 1556 (δN-H); 1233 (νC-O-C); ^1^H NMR (600 MHz, DMSO-*d*_6_) δ, ppm: 12.17 (s, 1H, OH), 11.70 (s, 1H, NH), 11.08 (s, 1H, OH), 9.35 (s, 1H, NH), 8.59 (s, 1H, CH), δ 7.36-7.37 (d, *J* = 8.9 Hz, 1H, Ar-H), 7.23 (s, 1H, Ar-H), 7.14–7.15 (d, *J* = 2.4 Hz, 1H, Ar-H), 6.99–7.00 (d, *J* = 2.4 Hz, 1H, Ar-H), 6.89–6.90 (d, *J* = 2.4 Hz, 1H, Ar-H), 6.87–6.88 (d, *J* = 2.5 Hz, 1H, Ar-H), 6.86–6.87 (d, *J* = 1.6 Hz, 1H, Ar-H), 6.85–6.86 (d, *J* = 1.6 Hz, 1H, Ar-H), 6.74–6.77 (t, *J* = 7.8 Hz, 1H), 3.77 (s, 3H, OCH_3_). ^13^C NMR (151 MHz, DMSO-*d*_6_) δ, ppm: 157.87, 154.43, 148.53, 146.42, 146.04, 132.75, 130.24, 127.78, 120.38, 119.73, 115.91, 113.79, 104.05, 102.51, 55.74, 55.74.

#### 2.3.8. N′-(3,4-Dihydroxybenzylidene)-5-methoxy-1H-indole-2-carbohydrazide (**5b**)

Yield 80%, Mp 252–253 °C, IR (ν_max_/cm^−1^) 3307 (νO-H); 3262 (νN-H); 2936 (ν_as_CH_2_); 2840 (ν_s_CH_2_); 1620 (νC=O) amide I; 1604 (νC=N); 1565 (δN-H); 1248 (νC-O-C); ^1^H NMR (600 MHz, DMSO-*d*_6_) δ, ppm: 11.62 (s, 1H, NH), 11.42 (s, 1H, NH), 9.73 (s, 1H, OH), 9.43 (s, 1H, OH), 8.25 (s, 1H, CH), δ 7.30-7.36 (m, 1H, Ar-H), 7.25-7.26 (d, *J* = 2.1 Hz, 1H, Ar-H), 7.18 (s, 1H, Ar-H), 7.04–7.11 (m, 1H, Ar-H), 6.95–6.99 (m, 1H, Ar-H), 6.86–6.88 (dd, *J* = 8.9, 2.4 Hz, 1H, Ar-H), 6.79–6.82 (m, 1H, Ar-H), 3.76 (s, 3H, OCH_3_). ^13^C NMR (151 MHz, DMSO-*d*_6_) δ, ppm: 157.90, 154.34, 154.20, 148.41, 148.11, 146.19, 132.52, 132.03, 130.96, 126.26, 121.06, 116.07, 115.50, 114.79, 113.70, 113.55, 113.16, 103.44, 102.46, 102.18, 55.73, 55.68.

#### 2.3.9. N′-(2,4-Dihydroxybenzylidene)-5-methoxy-1H-indole-2-carbohydrazide (**5c**)

Yield 85%, Mp 271–272 °C, IR (ν_max_/cm^−1^) 3326 (νO-H); 3256 (νN-H); 2939 (ν_as_CH_2_); 2834 (ν_s_CH_2_); 1631 (νC=O) amide I; 1610 (νC=N); 1565 (δN-H); 1210 (νC-O-C); ^1^H NMR (600 MHz, DMSO-*d*_6_) δ, ppm: 11.93 (s, 1H, NH), 11.66 (s, 1H, OH), 11.65 (s, 1H, NH), 10.00 (s, 1H, OH), 8.50 (s, 1H, CH), δ 7.34-7.36 (dd, *J* = 8., 4.37 Hz, 2H, Ar-H), 7.19 (s, 1H, Ar-H), 7.13–7.14 (d, *J* = 2.4 Hz, 1H, Ar-H), 6.87–6.88 (dd, *J* = 8.9, 2.5 Hz, 1H, Ar-H), 6.36–6.38 (dd, *J* = 8.4, 2.3 Hz, 1H, Ar-H), 6.33–6.34 (d, *J* = 2.3 Hz, 1H), 3.77 (s, 3H, OCH_3_). ^13^C NMR (151 MHz, DMSO-*d*_6_) δ, ppm: 161.13, 159.79, 157.58, 154.39, 148.63, 132.63, 131.54, 130.52, 127.80, 115.66, 113.72, 111.18, 108.20, 103.65, 103.11, 102.50, 55.74.

#### 2.3.10. N′-(4-Hydroxy-3-methoxybenzylidene)-5-methoxy-1H-indole-2-carbohydrazide (**5d**)

Yield 87%, Mp 261–261 °C, IR (ν_max_/cm^−1^) 3494 (νO-H); 3288 (νN-H); 2942 (ν_as_CH_3_); 2919 (ν_as_CH_2_); 2835 (ν_s_CH_3_); 1633 (νC=O) amide I; 1602 (νC=N); 1548 (δN-H); 1225, 1201 (νC-O-C); ^1^H NMR (600 MHz, DMSO-*d*_6_) δ, ppm: 11.70 (s, 1H, NH), 11.63 (s, 1H, NH), 9.59 (s, 1H, OH), 8.35 (s, 1H, CH), δ 7.35–7.36 (d, *J* = 8.9 Hz, 1H, Ar-H), δ 7.33–7.34 (d, *J* = 1.9 Hz, 1H, Ar-H) 7.21 (s, 1H, Ar-H), 7.10–7.13 (m, 2H, Ar-H), 6.85–6.88 (m, 2H, Ar-H), 3.84 (s, 3H, OCH_3_), 3.77 (s, 3H, OCH_3_). ^13^C NMR (151 MHz, DMSO-*d*_6_) δ, ppm: 157.89, 154.36, 149.43, 148.53, 148.17, 132.56, 130.98, 127.81, 126.22, 122.62, 115.94, 115.50, 113.70, 109.40, 103.50, 102.48, 56.03, 55.74.

#### 2.3.11. N′-(2-Hydroxy-4-methoxybenzylidene)-5-methoxy-1H-indole-2-carbohydrazide (**5e**)

Yield 85%, Mp 248–249 °C, IR (ν_max_/cm^−1^) 3285 (νO-H); 3186 (νN-H); 2963 (ν_as_CH_3_); 2936 (ν_as_CH_2_); 2839 (ν_s_CH_2_); 1643 (νC=O) amide I; 1632 (νC=N); 1549 (δN-H); 1225 (νC-O-C); ^1^H NMR (600 MHz, DMSO-*d*_6_) δ, ppm: 12.01 (s, 1H, NH), 11.67 (s, 1H, OH), 11.54 (s, 1H, NH), 8.55 (s, 1H, CH), δ 7.45–7.47 (d, *J* = 8.6 Hz, 1H, Ar-H), δ 7.35-7.37 (d, *J* = 8.8 Hz, 1H, Ar-H) 7.21 (s, 1H, Ar-H), 7.14–7.15 (d, *J* = 2.5 Hz, 1H, Ar-H), 6.87–6.89 (dd, *J* = 8.9, 2.5 Hz, 1H, Ar-H), 6.53–6.55 (dd, *J* = 8.6 2.5 Hz, 1H, Ar-H), 6.50–6.51 (d, *J* = 2.4 Hz, 1H, Ar-H), , 3.78 (s, 3H, OCH_3_), 3.77 (s, 3H, OCH_3_). ^13^C NMR (151 MHz, DMSO-*d*_6_) δ, ppm: 162.49, 159.71, 157.67, 154.40, 148.25, 132.68, 131.35, 130.43, 127.80, 115.73, 113.74, 112.44, 106.94, 103.78, 102.50, 101.64, 55.78, 55.7

#### 2.3.12. N′-(4-Hydroxy-3,5-dimethoxybenzylidene)-5-methoxy-1H-indole-2-carbohydrazide (**5f**)

Yield 87%, Mp 239–240 °C, IR (ν_max_/cm^−1^) 3493 (νO-H); 3298 (νN-H); 2963 (ν_as_CH_3_); 2939 (ν_as_CH_2_); 2834 (ν_s_CH_2_); 1641 (νC=O) amide I; 1627 (νC=N); 1545 (δN-H); 1213 (νC-O-C); ^1^H NMR (600 MHz, DMSO-d_6_) δ, ppm: 11.74 (s, 1H, NH), 11.63 (s, 1H, NH), 8.95 (s, 1H, CH), 8.34 (s, 1H, OH), δ 7.35-7.36 (d, *J* = 8.9 Hz, 1H, Ar-H) 7.22 (s, 1H, Ar-H), 7.13–7.14 (d, *J* = 2.4 Hz, 1H, Ar-H), 7.99 (s, 1H, Ar-H), 6.87–6.88 (dd, *J* = 8.7, 2.4 Hz, 1H, Ar-H), 3.83 (s, 6H, OCH_3_), 3.77 (s, 3H, OCH_3_). ^13^C NMR (151 MHz, DMSO-d_6_) δ, ppm: 157.92, 154.37, 148.64, 148.33, 138.37, 132.58, 132.58, 130.95, 127.80, 125.05, 115.53, 113.70, 105.06, 103.58, 102.48, 56.51, 55.74.

### 2.4. Red Blood Cell Hemolysis Assay

The potential of the test compounds to induce hemolysis was evaluated following the protocol described by Evans et al. [34]. The blood was collected from the heart of male Wister rats. The red blood cells were separated from the blood by repeated centrifugation in 0.9% NaCl buffer and were resuspended in phosphate-buffered saline (PBS, pH 7.4). The test compounds in concentrations 10, 100, and 200 µM, a negative control (distilled water), and a positive control (20% Triton X-100) were pipetted into 96-well plates. Then, the erythrocyte suspension in PBS was added, and the plates were incubated for 1 h at 37 °C. After centrifugation (5 min at 500× *g*), the supernatant was transferred to new 96-well plates in order to measure hemoglobin absorbance at 430 nm on a Synergy 2 plate-reader (BioTek Instruments, Inc., Highland Park, Winooski, VT, USA). The obtained results were presented as a percentage of hemolysis relative to the hemoglobin absorbance values in the positive controls. Hemoglobin absorbance of negative controls was accepted as zero hemolysis. According to ISO 10993-5, the compounds that caused hemolysis below 5% were considered biocompatible [35].

### 2.5. Cell lines and Culturing Conditions

Neuroblastoma cell line SH-SY5Y (94030304) was acquired from the European Collection of Cell Cultures (ECACC, Salisbury, UK). Cells were cultured in a medium prepared with RPMI basic media supplemented with 10% heat-inactivated FBS (fetal bovine serum), 2 mM L-glutamine, 1% antibiotics (penicillin/streptomycin), and incubated at 37 °C in a humidified atmosphere containing 5% of CO_2_. The culture’s medium was replaced at a time interval of 2–3 days.

Mouse-brain endothelial cell line bEnd3 [36] (CRL-2299) was purchased from the American Type Culture Collection (ATCC, Manassas, VA, USA). The cells were cultured in DMEM (Dulbeco’s modified essential medium) (cat. no. 30-2002, ATCC, Manassas, VA, USA), supplemented with 10% heat-inactivated FBS (cat. no. F7524, Sigma-Aldrich Co. LLC, St. Louis, MO, USA), 1 mM L-glutamine (cat. no. G7513, Sigma-Aldrich Co. LLC, St. Louis, MO, USA) and 1× Antibiotic-Antimycotic solution (penicillin: streptomycin: amphotericin B (100 U/mL: 0.10 mg/mL: 0.25 µg/mL) (cat. no. A5955, Sigma-Aldrich Co. LLC, St. Louis, MO, USA) at 37 °C in a humidified atmosphere containing 5% CO_2_. The cells were passaged in 75 cm^2^ flasks every 3–5 days via trypsinization (StableCell Trypsin solution, cat. no. T2610, Sigma-Aldrich Co. LLC, St. Louis, MO, USA).

### 2.6. In Vitro Cell Viability Assay

Cell viability was assessed via the mitochondrial reductase-dependent reaction of (3-(4,5-dimethylthiazol-2-yl)- 2,5-diphenyl tetrazolium bromide (MTT, cat. no. 475989, Calbiochem, Merck KGaA, Darmstadt, Germany) [37]. The SH-SY5Y and bEnd3 cell lines were seeded in 96-well plates at a density of 0.7 × 10^4^ (bEnd3) or 2 × 10^4^ (SH-SY5Y) cells/well and allowed to adhere for 24 h at 37 °C, 5% CO_2_. Then, the cells were incubated with the compounds (1–500 μM) for a preset time (24 h). For the solvent, the control cells were treated with DMSO corresponding to the highest concentration of DMSO in the samples (0.5% DMSO) in order to exclude its cytotoxic effect on the cells. For each concentration, a set of at least 6 wells were used. After incubation, the solution from each well was exchanged with fresh culture medium (100 µL/well), containing the MTT reagent (0.5 mg/mL) and incubated for 3 h at 37 °C in a humidified atmosphere and 5% CO_2_. The obtained formazan crystals were dissolved in 100 µL/well of 5% formic acid in propan-2-ol solution (or DMSO). The absorbance was measured at 570 nm via multiplate reader Tecan Infinite F200 PRO (Tecan Trading AG., Männedorf, Switzerland) or Synergy 2 (BioTek Instruments, Inc., Highland Park, Winooski, VT, USA).

### 2.7. H_2_O_2_-Induced Oxidative Stress Model in SH-SY5Y Cells

The SH-SY5Y cells were seeded in 96-well plates at a density of 3.5 × 10^4^/well in 100 μL of RPMI supplemented with FBS for 24 h. Then, the cell medium was aspirated, and the cells were treated with different concentrations of the compounds—1, 10, and 50 µM for 90 min. Afterwards, the SH-SY5Y cells were washed with PBS and were exposed to hydrogen peroxide (H_2_O_2_) (1 mM in PBS for 15 min) to induce oxidative stress. All well contents were replaced by fresh culture medium and incubated for 24 h. MTT assay was used to evaluate the amount of attached viable cells. Negative controls (cells without H_2_O_2_ treatment) were considered as 100% protection and H_2_O_2_-treated cells as 0% protection. Melatonin and rasagiline (Sigma-Aldrich Co. LLC, St. Louis, MO, USA) were used as references because of their potent antioxidant and neuroprotective effects, which were established by several in vitro and in vivo studies [17,38].

### 2.8. Ferrous Iron-Induced Oxidative Damage

The lipid peroxidation assay [39,40] and a modification of the deoxyribose-degradation assay [41] were used. Two types of measurement compositions in phosphate buffer (PB–K_2_HPO_4_/KH_2_PO_4_, pH 7.4) were prepared for both tests: (i) samples, containing the biologically relevant molecules (lecithin emulsion or deoxyribose), iron, and the tested hydrazones at the concentrations indicated below the figures; and (ii) controls, in which the tested hydrazones were omitted. The data for the sample activity were presented as a percentage of the control measurement.

### 2.9. Lipid Peroxidation Assay (LP Assay)

The thiobarbituric-acid reactive-substance colorimetric test was performed in a model system of Fe(II)-induced lipid peroxidation as an emulsion prepared from egg yolk phospholipids. Each sample contained 1 mg/mL of lecithin. LP was initiated using 0.1 mM FeCl_2_. After 30 min incubation at 37 °C, 0.5 mL of 2.8% trichloroacetic acid solution and 0.5 mL of 0.5% thiobarbituric acid solution were added, following a second incubation at 100 °C for 20 min. Centrifugation at 3000 rpm for 20 min at 4 °C was performed, and we determined the absorbance value of the supernatant at 532 nm via Shimadzu UV-1601 UV-VIS spectrophotometer.

### 2.10. Deoxyribose Degradation Assay

The deoxyribose (0.5 mM) damage was induced by adding FeCl_2_ (0.1 mM). The measurement compositions were incubated at 37 °C for 30 min. The next step comprised adding 0.5 mL of 2.8% trichloroacetic acid and 0.5 mL of thiobarbituric acid. Then, the samples were heated in boiling water for 20 min, and their absorbance was estimated at 532 nm (characteristic band for the generated chromophore) via Shimadzu UV-1601 UV-vis spectrophotometer.

### 2.11. O-Phenanthroline Test

The Fe (II)-chelating activity of the tested hydrazones was determined by the O-phenanthroline method [42]. A total of 1 mL sample solutions of 50 mM PB pH 7 contained 0.2 mM O-phenanthroline and 0.05 mM FeCl_2_ and 0.2 mM hydrazone. In the controls, the tested compounds were omitted. Absorbance was measured at 515 nm via GENESYS 50 UV-VIS spectrophotometer after incubation for 5 min at 37 °C. The data for the sample activity were presented as a percentage of the control measurement. Absorbance spectra of all the samples, the control, and the measurement compositions containing the tested hydrazones and Fe(II) at the concentrations used in the samples were measured.

### 2.12. Superoxide Radicals Scavenging Activity

The 1 mL cuvette for the control contained 1 mM xanthine, 20 µL xanthine oxidase (100 IU/L), and 0.04 mM NBT [43,44]. In the sample compositions, the tested hydrazones at different concentrations were added. All measurement compositions were incubated at 37 °C, and the absorbance was determined spectrophotometrically at 560 nm via Shimadzu UV-1601 UV-vis spectrophotometer.

### 2.13. Permeability Assay

To evaluate the compounds’ effect on a BBB model, bEnd3 endothelial cells were seeded on polycarbon (PC) cell-culture inserts with 0.4 µm pore size (cat. no. 1CH3.1, BRAND GMBH + CO KG, Wertheim, Germany) and a density of 5.6 × 10^3^ cells/insert. The inserts were placed in a 24-well plate containing 1 mL of cell-culture medium. The plate was then incubated for 4 days at 37 °C in a humidified atmosphere and 5% CO_2_. On the second day of incubation, the cell-culture medium was refreshed. To ensure 100% confluence, the inserts were evaluated for medium leakage before the experiment. If no leakage was observed, the cells were treated with 10 and 50 µM concentrations of the compounds and incubated for 90 min at 37 °C in a humidified atmosphere and 5% CO_2_. Following the incubation, the inserts were placed in a new 24-well plate containing 1 mL Phenol Red free–DMEM (cat. no. D1145, Sigma-Aldrich Co. LLC, St. Louis, MO, USA) without serum. In the upper chamber, a solution of streptavidin, conjugated with horse-radish peroxidase (HRP, cat. no. SA00001-0, Proteintech, Rosemont, Illinois, USA) (1:1000) in serum-free phenol red medium was added. The cells were further incubated for 5 min at 37 °C and 5% CO_2_, after which the inserts were removed. The remaining medium in the 24-well plate was collected and vortexed briefly, and 3 × 50 µL aliquots were transferred into a 96-well plate. A total of 50 µL TMB Signal+ substrate solution (BUF054A, Bio-Rad, Hercules, CA, USA) was added to each well and incubated for 5 min at 37 °C. The HRP converted the TMB to a blue-colored product. To stop the reaction, 50 µL of 0.2 N H_2_SO_4_ was added that caused the blue color to turn yellow. The absorption was measured at 450 nm via multiplate reader Tecan Infinite F200 PRO (Tecan Trading AG., Männedorf, Switzerland). Each condition was tested at least twice.

### 2.14. Immunofluorescence

Immunofluorescence of zonula occludens-1 (ZO-1) was performed to determine the in vitro effect of the compounds on tight junctions (TJs) between bEnd3 cells. The experiment was conducted simultaneously with the permeability assay. Briefly, cells were seeded on cover slides in 24-well plates at a concentration of 3.5 × 10^4^ cells/well and incubated for 3–4 d at 37 °C and 5% CO_2_ in a humidified atmosphere, until they reached 100% confluence. The cells were then treated with 10 and 50 µM concentrations of the compounds for 90 min. After the allotted treatment time, the cells were fixed with ice-cold acetone at −20 °C for 10 min; blocked with 1% bovine serum albumin (BSA, cat. no. 9048-46-8, Acros Organics, Thermo Fischer Scientific, Waltham, MA, USA) in PBS, containing 0.1% Tween^®^ 20 (cat. no. P1379, Sigma-Aldrich Co. LLC, St. Louis, MO, USA); and labeled with anti-ZO-1 (rabbit polyclonal antibody, 1:500, cat. no. ab96587, Abcam, Cambridge, UK) at 4 °C overnight. The slides were then incubated with a secondary donkey anti-rabbit Alexa Fluor™ 555 antibody (1:1000, cat. no. 4413S, Molecular Probes, Thermo Fischer Scientific, Waltham, MA, USA) for 1 h at room temperature (RT) in the dark. Finally, cell nuclei were stained for 5 min/RT/dark with 1 µg/mL 4′,6-diamidino-2-phenylindole (DAPI, D9542, Sigma-Aldrich Co. LLC, St. Louis, MO, USA). The slides were washed with 3 x PBS between each step. Finally, the samples were mounted via Mowiol^®^4-88 (cat. no. 81381, Sigma-Aldrich Co. LLC, St. Louis, MO, USA). Fluorescence microscopy was performed on a spinning disk confocal microscope system (Andor Dragonfly 505, Oxford Instruments, Abingdon, UK) with 60 × oil NA 1.4 objective, with em./ex. = 561/617 nm for ZO-1 and 405/445 nm for DAPI. The obtained data were analyzed with Fiji [45] and compiled via the FigureJ plugin [46].

### 2.15. hMAO-B Enzyme Activity Inhibition

Inhibition of monoamine oxidase activity assay of recombinant human MAO-B (hMAO-B) was monitored by a fluorimetric assay that was slightly modified according to [47]. Amplex^®^ UltraRed reagent [48] was used for detection of the hydrogen peroxide and peroxidase activity in biological samples and enzymes. In the presence of peroxidase, a red phosphorescent oxidation product, resorufin, was produced due to the 1:1 stoichiometric reaction of Amplex^®^ Red with hydrogen peroxide. The analysis was conducted spectrophotometrically based on the high extinction coefficient of the color product. Using this method, small amounts of hydrogen peroxide (of the order of 10 pM in a volume of 100 µL) could be detected.

The following controls were used: pure MAO-B working solution in reaction buffer, MAO-B working solution containing hydrogen peroxide, and pure reaction buffer. The final concentration of the substances was 1 µM. The substances, together with hMAO-B, were instilled into a 96-well plate (8 samples for each substance), after which the plate was placed in an incubator for 30 min (in the dark at 37 °C). At the end of the incubation period, the reaction was started by adding a 50 µL of a mix solution containing solutions of Amplex^®^ Red reagent, horseradish peroxidase (HRP), and tyramine as a substrate for the enzyme in a reaction buffer, to each well on the 96-well plate. The reaction kinetics were monitored by fluorescence measurements at 30 min intervals (0, 30, 60, 90, 120, and 150 min, respectively) in the dark while constantly shaking the reaction mixture at 37 °C. Fluorimetric measurements were performed on a Synergy 2 Microplate Reader, at two different wavelengths (λ = 570 nm and λ = 690 nm) [49].

### 2.16. Animals

Male Wistar rats (7 months old, body weight 250–300 g) were provided by the National Breeding Center, Sofia, Bulgaria. Food and water were provided ad libitum. A minimum of eight days of acclimatization were allowed before the study. The animals’ health was regularly monitored by a veterinary physician.

The animals were anesthetized with pentobarbital, and the brains were perfused through the left ventricle with tris-buffered saline before decapitation. The brains were quickly removed, rinsed with the perfusion buffer, and used for the preparation of synaptosomes. The manipulations were carried out at 4 °C, in accordance with approved ordinance of the Institutional Animal Care Committee (Ordinance No. 15/2006 for the humane treatment of experimental animals (vivarium certificate of registration of farm No. 0072/1 August 2007)). All experiments with isolated rat-brain synaptosomes were conducted in accordance with the ethical approval № 323 from the Bulgarian Agency for Food Safety (valid until 22 December 2026).

### 2.17. Isolation of the Rat-Brain Synaptosomes

Preparation of the synaptosomes was carried out by multiple centrifugations using the Percoll gradient. Homogenization of the brains was performed in 10 volumes of cold buffer 1, which contained 5 mM HEPES and 0.32 M sucrose (pH = 7.4), followed by centrifugation at different speeds. For preparation of the gradient, the synaptosomes were isolated by Percoll reagent. Synaptosomes were resuspended and incubated in buffer 2, which contained 290 mM NaCl, 0.95 mM MgCl_2_x6H_2_O, 10 mM KCl, 2.4 mM CaCl_2_xH_2_O, 2.1 mM NaH_2_PO_4_, 44 mM HEPES, and 13 mM D-glucose. Incubations were performed in a 5% CO_2_ + 95% O_2_ atmosphere [50]. The method of Lowry et al., using bovine serum albumin as a standard content, was applied for determination of synaptosomal protein [51].

### 2.18. Measurement of Synaptosomal Viability

Synaptosomal viability was monitored based on a (3-(4,5)-dimethylthiazol-2-yl)-2,5-diphenyl tetrazolium bromide) assay [52]. After incubation with the compounds, synaptosomes were treated with 0.5 mg/mL solution of (3-(4,5)-dimethylthiazol-2-yl)-2,5-diphenyl tetrazolium bromide) for 10 min at 7 °C. Then, the synaptosomes were centrifuged twice at 15,000× *g* for 1 min. The resulting formazan crystals were dissolved in DMSO (dimethyl sulfoxide), and the extinction was determined by spectrophotometric measurements at 580–660 nm.

### 2.19. Level of Reduced Glutathione (GSH) in Isolated Synaptosomes

After the treatment with the tested compounds, the glutathione levels of the synaptosomes were determined with an Ellman reagent (DTNB) [53]. The GSH levels were evaluated based on the synaptosomal sediment, precipitated with 5% TCA, and centrifuged for 10 min at 4000× *g*. GSH levels in the supernatant were measured spectrophotometrically at 412 nm by a Spectro UV-vis split spectrophotometer (LaboMed. Inc., Los Angelis, California, USA).

### 2.20. Model of 6-Hydroxy Dopamine-Induced Neurotoxicity in Isolated Rat Synaptosomes

This neurotoxicity model was based on the metabolism and auto-oxidation of 6-hydroxy dopamine responsible for the reactive oxygen species and quinones generation, ultimately leading to neurotoxicity and neurodegeneration [54]. The experiment was performed by pre-incubation of the synaptosomes with 10 µM of the compounds for 30 min, followed by the addition of 150 µM 6-hydroxy dopamine for induction of neurotoxicity [31]. After 1 h, they were centrifuged twice for 1 min at 15,000 × *g*. After the second wash, the precipitates were resolved with buffer B and glucose.

### 2.21. Statistical Analysis

All experiments were carried out in triplicate (unless otherwise stated in Materials and Methods), and the results were presented as mean ± SD (n = 6). One-way ANOVA, followed by a Dunnett’s post hoc test, was applied in the statistical analysis. The GraphPad 6 software (GraphPad Software, Inc., La Jolla, CA, USA) was used for statistical evaluation. Differences between groups were considered significant at *p* < 0.001.

### 2.22. Molecular Docking

Ligands were protonated according to their protonation state at 7.4 pH, and a set of conformations was compiled for the docking study using a systematic search over all rotatable bonds of the ligands. The optimization of all the initially generated ligands was achieved by MMFF94x force field to the gradient of 0.01, where all geometries with an RMSD of more than 0.25 were considered different. The energy window for the collection of conformations for the further docking analysis was set to 5 kcal/mol from the lowest found energy conformation of the ligand.

Molecular docking was carried out with the structure with PDB number 3PO7, which is a human monoamine oxidase B in complex with zonisamide obtained by XRD and with an overall resolution of 1.8 Å [55] by Molecular Operating Environment (MOE) software package [56]. The structure was protonated (pH 7.0, 300K, Salt 0.1M/L) using a 3D protonation algorithm implemented in the MOE package.

The ligands (in all respective conformations) were docked at the active site of MAO-B using the triangle matcher algorithm for the initial placement of structures. The algorithm returned up to 10^6^ poses of the ligand inside the pocket. London dG [56] function was used to score the respective poses, based on the estimation of the free energy of binding of the ligand from a given pose and consisting of terms for evaluation of the average gain/loss of rotational and translational entropy and loss of flexibility of the ligand, geometric imperfections of hydrogen bonds, and desolvation energy of atoms.

The best 50 poses for every ligand were further optimized with induced fit methodology, using MMFF94x force field and reaction field solvation model. The residues (including FAD molecule) that had atoms closer than 6 Å from any atom of the ligand were considered free during optimization, while the others were considered fixed. The GBVI/WSA dG [56] was used as a rescoring function, and the best 30 poses were collected for further analysis.

## 3. Results

### 3.1. Synthesis of the Target Compounds

**Scheme 2:**
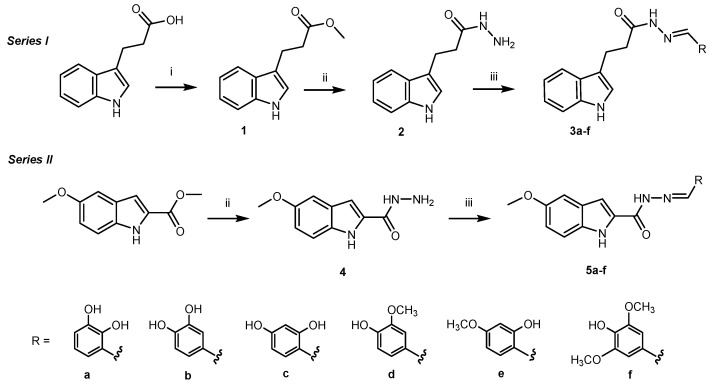
Synthesis of new arylhydrazones of IPA (Series I) and 5-methoxy-indole-2-carboxylate (Series II). Reagents and conditions: (i) thionyl chloride, methanol, refluxing; (ii) hydrazine hydrate, ethanol solution, refluxing; (iii) corresponding aldehyde, ethanol, refluxing.

The synthesis of the indole hybrids was carried out, as shown in Scheme 2. The IPA was transformed into a methyl ester using a widely known reaction with thionyl chloride and methanol. The hydrazinolysis of the ester compounds of both series with hydrazine hydrate in ethanol yielded the hydrazides **2** and **4**, which were then condensed with the respective methoxy and hydroxy-substituted benzaldehydes for obtaining the 12 target compounds, **3a–f** and **5a–f**. The structures of the products were confirmed by ^1^H and ^13^C NMR spectroscopy (Figures S1, S11 and S12, Appendix A).

### 3.2. Safety Profile

**Figure 1:**
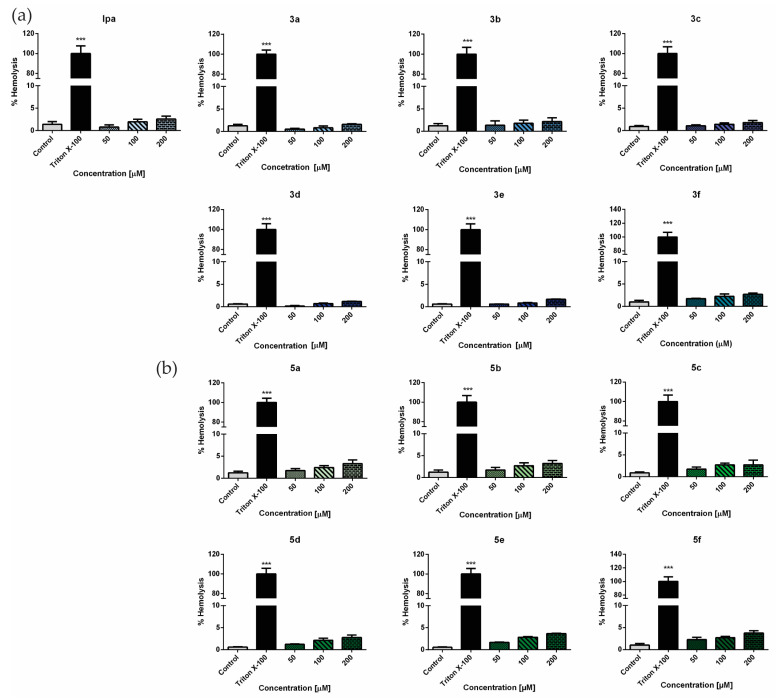
Hemolytic effects of (**a**) IPA and compounds **3a–f**; (**b**) compounds **5a–f**; on human erythrocytes. The results are expressed as means ± SD of triplicate assays (n = 3). All groups were compared statistically vs. untreated controls by one-way ANOVA with Dunnet’s post hoc test *** *p* < 0.001 vs. control.

Hemolysis assay, using red blood cells, is an essential part of the in vitro biocompatibility testing of new drug substances; and it was used as a screening method for the evaluation of potential in vivo toxicity. The release of hemoglobin was a definite sign of the cell lysis of the erythrocytes and could be measured spectrophotometrically [57]. For this purpose, the cell suspension (red blood cells in PBS) was incubated with the test compounds at concentrations of 50, 100, and 200 µM at 37 °C for 1 h. As compared to the positive control (Triton X-100), which induced complete hemolysis (100%), all tested newly synthesized compounds showed no or very low hemolytic effects (below 5%), even at the highest concentration (200 μM) (Figure 1).

Furthermore, the safety profiles of the newly synthesized compounds were examined based on the cell viability of the human neuroblastoma SH-SY5Y cell line and the immortalized mouse-brain microvascular endothelial cell line bEnd3. The cytotoxic effects of the compounds were evaluated via the MTT cell viability assay.

Both the SH-SY5Y and bEnd3 cells were treated with the test compounds in a concentration range from 1 to 500 µM for 24 h. Thereafter, the corresponding IC_50_ values were calculated (Table 1). The compounds **3e** and **3f**, as well as the IPA, did not show any significant cytotoxic effects, as the estimated IC_50_ values were >500 µM, followed by compounds **3b** (265.3 µM), **3c** (275.2 µM), and **5f** (286.9 µM). The relatively higher cytotoxic effect was exhbited by compounds **5a** (79.3 µM), **5c** (73.9 µM) and **5d** (98.2 µM).

Interestingly, bEnd3 showed higher sensitivity to the compounds **3e** and **5e** (approximately 6- and 200-fold, respectively), as compared to the SH-SY5Y cells, and these compounds could be considered toxic to bEnd3 cells. In contrast, bEnd3 had a lower toxicity response with **5c** and **3c**, as compared to SH-SY5Y (Table 1). For both cell lines, **3f** did not exhibit any cytotoxic effect.

### 3.3. Neuroprotective Effects in H_2_O_2_-Induced Oxidative Stress on SH-SY5Y Cells and 6-OHDA-Induced Neurotoxicity in Rat-Brain Synaptosomes

**Figure 2:**
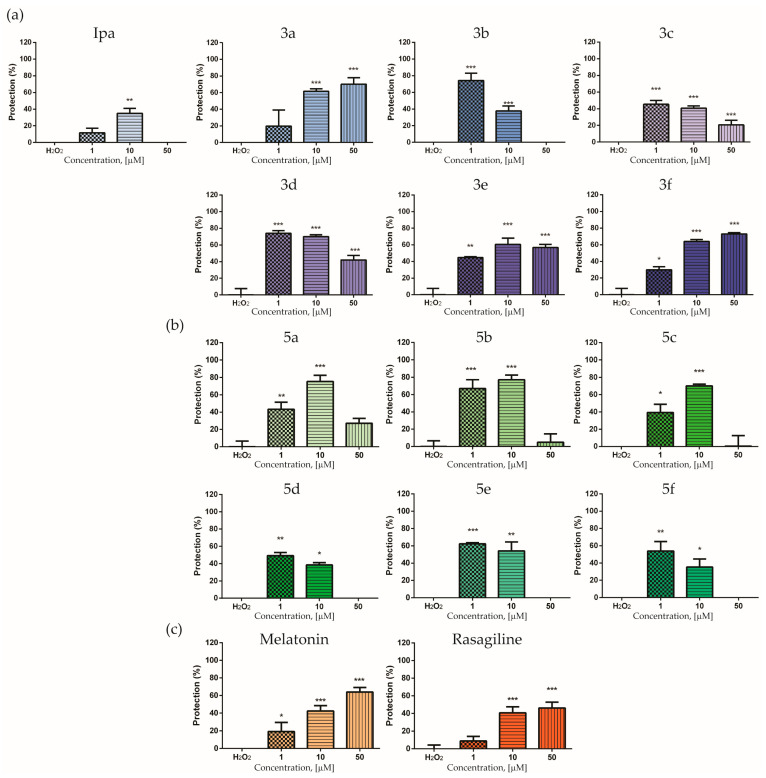
Protective effects of (**a**) IPA and compounds **3a–f**; (**b**) compounds **5a–f**; and (**c**) reference melatonin and rasagiline at different concentrations (1, 10, and 50 µM), in a model of H_2_O_2_-induced oxidative stress in human neuroblastoma SH-SY5Y cells. Data are presented as means ± SEM of three independent experiments. * *p* < 0.05, ** *p* < 0.01, *** *p* < 0.001, vs. H_2_O_2_-treated cells (one-way analysis of variance with Dunnet’s post hoc test).

To evaluate the possible neuroprotective effects of the compounds, the SH-SY5Y cells were pre-treated with different concentrations (1, 10, 50 µM) for 90 min and then exposed to H_2_O_2_ (1 mM) for 15 min. The cell viability of all compounds was measured to verify their protective effect against the toxicity of hydrogen peroxide. The viability of SH-SY5Y cells was drastically reduced after exposure to H_2_O_2_ (Figure 2). Pre-treatment for 90 min with the reference compound melatonin (1, 10, and 50 µM) improved the cells’ resistance towards H_2_O_2_, as portrayed by the significantly increased cell viability of SH-SY5Y (*p* < 0.05; *p* < 0.001 vs. H_2_O_2_ group) at 19%, 42%, and 64%, respectively. Similarly, rasagiline demonstrated a better protective effect at 10 and 50 µM concentrations (40% and 46%, respectively). Furthermore, pre-incubation with all tested compounds preserved the SH-SY5Y cells, suggesting a possible neuroprotective effect. For the first series of compounds (**3a–f**), the protective effect was more pronounced, as compared to the 3 reference compounds IPA, melatonin, and rasagiline. The compounds of series II (**5a–f**) were highly potent at the lower concentrations of 1 and 10 µM.

Some of our compounds showed reduced protection at the highest tested concentration (50 µM) in the model of H_2_O_2_-induced oxidative stress in the neuroblastoma cell line SH-SY5Y. In general, such an effect could have been related to the intrinsic cytotoxicity of the compounds. However, as the magnitude of the observed effect was not consistent with the established cytotoxicity order and the applied concentration was still better than the IC_50_ values, other reasons for this effect were also considered. A possible explanation could be the dual nature of antioxidants. Antioxidants are substances that, when present in low concentrations, delay or prevent oxidative damage caused by the presence of ROS. Some antioxidants at high doses or in the presence of metal ions have displayed pro-oxidant activities. The antioxidant or pro-oxidant activity closely depends on their concentration [58]. These data were available for some natural antioxidants. For instance, in various studies, quercetin exhibited antioxidant activity in vitro at low concentrations (0.1–20 μM), while at higher concentrations (>50 μM), it reduced cell survival and viability, as well as decreased the thiol content, total antioxidant capacity, and SOD, CAT, and glutathione S-transferase activities [59]. The potential pro-oxidant effects of exogenous antioxidants have also been found in some synthetic antioxidants, including BHA (butylated hydroxyanisole) and BHT (butylated hydroxytoluene) [60]. For certain studied compounds, which had less expressed radical-scavenging properties (see Section 3.4), the reduced neuroprotection at higher concentrations might have been related to the exhaustion of their antioxidant capacity.

In a similar manner, the studied compounds at higher concentrations could have inhibited the catalase and thus decreased its ability to convert H_2_O_2_ to H_2_O+O_2_ and to exert antioxidant activity. However, this hypothesis would need an additional experiment to be confirmed.

The stability of the compounds was also considered a potential issue. Therefore, selected compounds (**3a**, **3d**, **5d**, and **5e** at a concentration of 50 µM) were studied by UV-vis measurements under different pH conditions, including pH 2, 4.5, 7.4, and 8.4, to represent different physiological (gastric, intestinal, etc.) conditions. The UV-vis spectra of the compounds measured after 1, 2, 3, and 24 h (Figures S38–S41 in the Supplementary Materials) showed that most of the compounds were stable over time, and the spectra did not show substantial differences from the initial values in the corresponding solutions (**3a**, **3d**, and **5e**). Notably, **5e**, one of the compounds lacking a neuroprotective effect at the highest concentration, showed very good stability. After taking into account all the potential causes and the observed correlations, it could be concluded that the decrease in neuroprotection at a concentration of 50 M could not be linearly described by a single factor and had a more complicated character.

**Figure 3:**
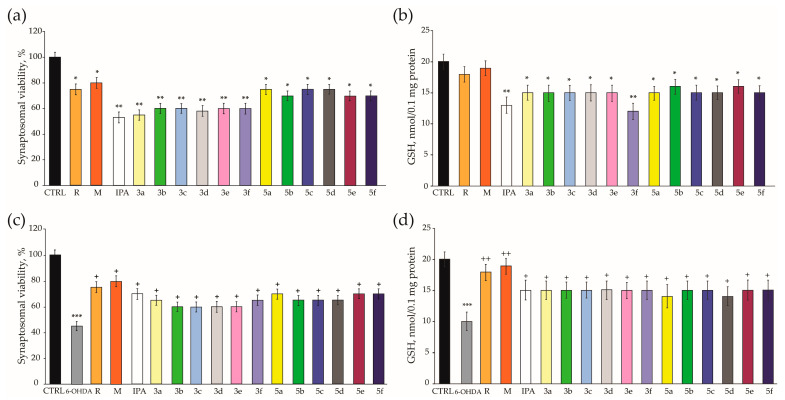
Effects of the tested compounds, IPA, rasagiline (R) and melatonin (M), administered alone at a concentration of 100 µM, on synaptosomal viability (**a**) and GSH levels (**b**). Effects of the exam compounds, IPA, rasagiline, and melatonin, at a concentration of 10 µM, in 6-OHDA-induced oxidative stress, on synaptosomal viability (**c**) and GSH levels (**d**). * *p* < 0.05; ** *p* < 0.01, *** *p* < 0.001 vs. control (non-treated synaptosomes); + *p* < 0.05; ++ *p* < 0.01 vs. 6-OHDA.

The low toxicity and substantial neuroprotective properties shown by the compounds in human neuroblastoma SH-SY5Y cells motivated the further characterization on a different cellular in vitro model, using freshly isolated rat-brain synaptosomes. As a first step, the compounds’ toxicity was evaluated by administering the exam compounds alone (in 100 µM concentration) and monitoring the synaptosomal viability (SV) (measured by MTT assay) and the level of reduced glutathione (GSH) in the synaptosomes, as compared to the control (non-treated synaptosomes) (Figure 3a,b). All exam compounds showed statistically significant effects on the functional-metabolic status of the isolated synaptosomes. Only rasagiline and melatonin did not decrease the GSH levels while reducing the SV by 25% and 20%, respectively. The compounds **5a–f** showed lower toxicity by reducing SV by 25–30% than the IPA derivatives **3a–f** (SV decrease of approx. 40%). The neurotoxicity of the exam compounds and IPA (47% reduction in SV) was higher than those of rasagiline and melatonin. Meanwhile, the GSH levels were primarily affected by **3f**, exhibiting a 40% reduction. The rest of the compounds decreased the GSH levels by approx. 20–25%.

Administered alone at a concentration of 150 µM, 6-hydroxydopamine (6-OHDA) revealed a statistically significant neurotoxic effect on the isolated rat-brain synaptosomes by decreasing the SV and GSH levels by 55% and 50%, respectively, as compared to the control (non-treated synaptosomes) (Figure 3c,d). In this model of neurotoxicity, synaptosomes were pre-incubated with 10 µM from the exam compounds, IPA, rasagiline I and melatonin (M). All exam compounds exhibited statistically significant neuroprotective effects, as shown by the SV and GSH levels (Figure 3c,d). Here, the references, IPA, rasagiline, and melatonin displayed better neuroprotection against 6-OHDA-generated ROS products, with SVs of 56%, 67%, and 78%, respectively. The GSH levels also remained relatively high in the presence of 6-OHDA and measured 50% for IPA, 80% for rasagiline, and 90% for melatonin, as compared to the toxic agent. Compounds **3a**, **3f**, **5b**, **5c**, and **5d** preserved the SV by 44%, whereas **3b**, **3c**, **3d**, and **3e** managed only by 33%. All compounds performed similarly to IPA in relation to the GSH levels, except **5a** and **5d** (40% protection, as compared to 6-OHDA). The highest defense of SV was observed for **5a**, **5e**, and **5f**, at 56%, as compared to 6-OHDA.

### 3.4. Iron-Induced Oxidative Damage of Lecithin and Deoxyribose

**Figure 4:**
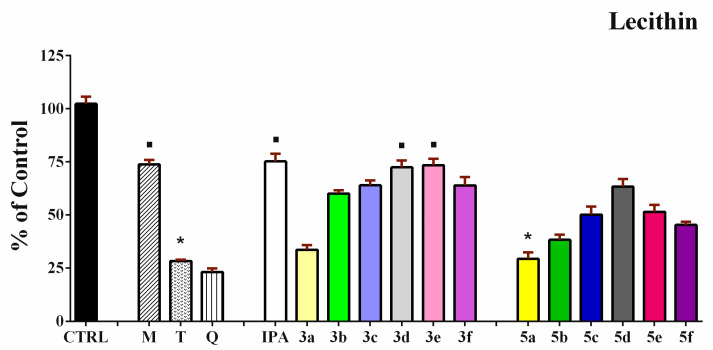
Effect of the tested compounds (90 µM) in the in vitro model system of Fe(II)-induced lecithin (1 mg/mL) peroxidation. Data concerning the effect of the references melatonin (M), Trolox (T) and quercetin (Q) were presented All data are expressed as means ± SEM. The effect of compounds labeled with ∎ is considered statistically identical to the effect of melatonin. Respectively, * labels the compounds with an effect statistically identical to Trolox.

In the lecithin-containing model system, a decrease in the determined parameter (sample/control absorbance ratio by percentage) was observed, as compared to the controls. All indole derivatives from both structural groups suppressed iron-induced lipid peroxidation and demonstrated antioxidant properties. The absorbance ratio varied from 30% for the most effective compound to 75% for the compounds with the lowest antioxidant activity (Figure 4). The effects of the three compounds possessing well-established antioxidant properties (melatonin, Trolox, and quercetin) were also determined for comparison.

The effectiveness of all studied structural modifications of the 5-methoxy-indole carboxylic acid derivatives **5a–f** was more pronounced (lower absorbance ratio values) than the analogous structures in the IPA derivatives **3a–f**. The absorbance ratio ranged from 34 to 74% for **3a–f** and from 29 to 65% for **5a–f**. As compared to the individual results of the derivatives of the IPA series, we could conclude that the structural modifications resulted in fewer expressed differences in the observed effects, except for the 2,3-dihydroxy hydrazone **3a**. We found two subgroups of derivatives with statistically identical potency via their absorbance ratios below 65% (**3b**, **3c**, and **3f**) and absorbance ratios of about 75% (**3d** and **3e**). Among the 5MIC derivatives, only 1 subgroup of compounds, comprising **5c**, **5e** and **5f**, exhibited the same extent of LP suppression, as observed. The absorbance ratio for these derivatives was about 45%.

The reference melatonin revealed the lowest antioxidant effect in the studied system (absorbance ratio 75%). In the presence of IPA and 2 other compounds from the IPA derivatives,**3d** and **3e**, the further amelioration of the antioxidant properties was not observed, as compared to melatonin. None of the studied compounds reached the effectiveness of the most potent reference, quercetin (23%). The two 2,3-dihydroxy compounds from both series (**3a** and **5a**) had the same effects statistically (absorbance ratios around 30%) and revealed themselves as the most prospective derivatives in the system. The effectiveness of **5a** was equivalent to the reference Trolox.

The capability of the compounds to preserve deoxyribose molecules from iron-induced oxidative damage was estimated using the deoxyribose-degradation test. For this purpose, we applied a protocol initiating oxidative damage with only ferrous ions [61,62,63].

**Figure 5:**
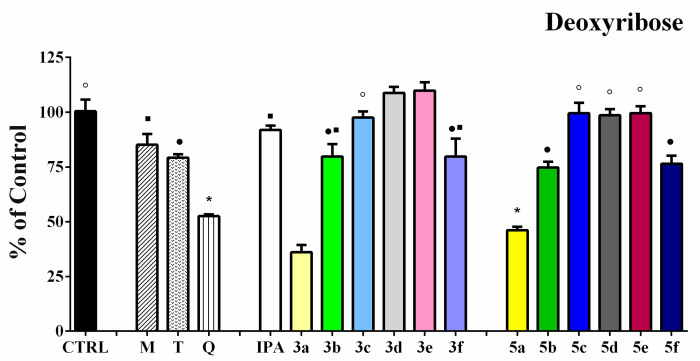
Effect of the tested hydrazones derivatives (80 µM) on the in vitro Fe(II)-induced degradation of 2-deoxyribose (0.5 mM). Data concerning the effect of the used references melatonin (M), Trolox (T) and quercetin (Q) were presented. Data are presented as means ± SEM from three independent experiments. The effect of compounds labeled with ∎ is considered statistically identical to the effect of melatonin. Respectively, ◦, ● and * label the compounds with an effect statistically identical to Control, Trolox and quercetin.

Depending on the structural modifications of the compounds in the model system containing deoxyribose, we observed an antioxidant effect and, in some cases, a weak pro-oxidant effect (Figure 5). Among the IPA derivatives, only 1 compound had an identical effect to that of the controls, while among the 5MPI series, three compounds were identified. The compounds **3c**, **5c**, **5d**, and **5e** did not induce a modulating effect on the iron-induced damage of deoxyribose, compared to the controls. Among the 5MIC series, none of the tested compounds exhibited pro-oxidant activity. In contrast, in the 3 substituted series, 2 substances had a weak but statistically significant pro-oxidant effect (**3d** and **3e**), which was about 10% above the controls. IPA, **3b**, and **3f** showed a relatively low antioxidant effect and an efficiency equivalent to the reference melatonin (absorbance ratio about 85%).

Among the 5MIC derivatives **5a–f**, all hydrazones exhibiting antioxidant properties in the system had more pronounced efficiency results than melatonin. The compounds **3b**, **3f**, **5b**, and **5f** had statistically identical results to Trolox, as a % of the controls below 80%, and **5a** had the same effect as quercetin at about 50%. Only the potency of **3c** was statistically identical to the series-specific reference IPA. Several derivatives among the two series of compounds all had similar effects: the dihydroxy derivatives with hydroxyl groups in the 3- and 4-position (**3b** and **5b**); those with hydroxyl groups in the 2- and 4-position (**3c** and **5c**), and the derivatives containing a syringaldehyde residue (**3f** and **5f**). This indicated that the accompanying modifications had no additional modulating effect on the effectiveness of these compounds in the system.

The structural analogs possessing a catechol moiety (2,3- and 3,4-dihydroxy) and those containing a syringaldehyde residue had better antioxidant properties. The most potent antioxidant effect in the system was founds in **3a**, which was statistically distinguishable from the reference quercetin and more pronounced (absorbance ratio around 35%).

### 3.5. Ortho-Phenanthroline Test

To explain the results of both systems of Fe(II)-induced oxidative damage, we studied the potency of the tested compounds to chelate-iron (Fe(II)). This was studied by spectrophotometric determination of free iron (Fe(II)) using the ortho-phenanthroline test, as 1,10-phenantroline reacts rapidly with Fe(II) ions in weakly acidic media, generating an orange–red complex (ferroin) with characteristic absorbance spectra (molar absorptivity 1.10.104 (α = 0.20) at ~max 512 nm and a molar ratio 3:1 (R: Fe(II))). The generated complex was stable, and the bound Fe(II) was resistant to oxidation. Lower absorbance of the reaction mixture indicated higher Fe^2+^-chelating activity.

**Figure 6:**
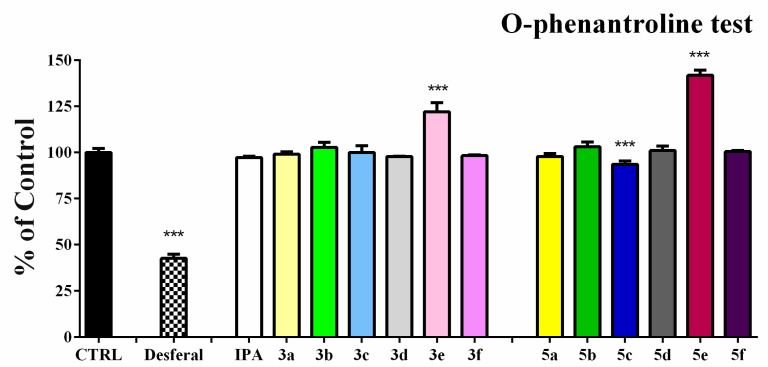
O-phenanthroline colorimetric chelation test in the presence of the tested compounds (200 µM): 1 mL of PBS buffer, pH 7.4, contains 0.02 mM 1,10-phenanthroline; 0.1 mM FeCl_2_; and the corresponding studied structural analogs. Desferal concentration was 10 µM. Data are presented as means ± SEM from three independent experiments. *** *p* < 0.001 vs. control. A one–way analysis of variance with Dunnet’s post hoc test was performed.

The obtained absorbance values measured at 515 nm for most compounds were statistically identical to the control samples (Figure 6). Only for 3 compounds, **3e**, **5c**, and **5e**, we observed statistically significant changes.

**Figure 7:**
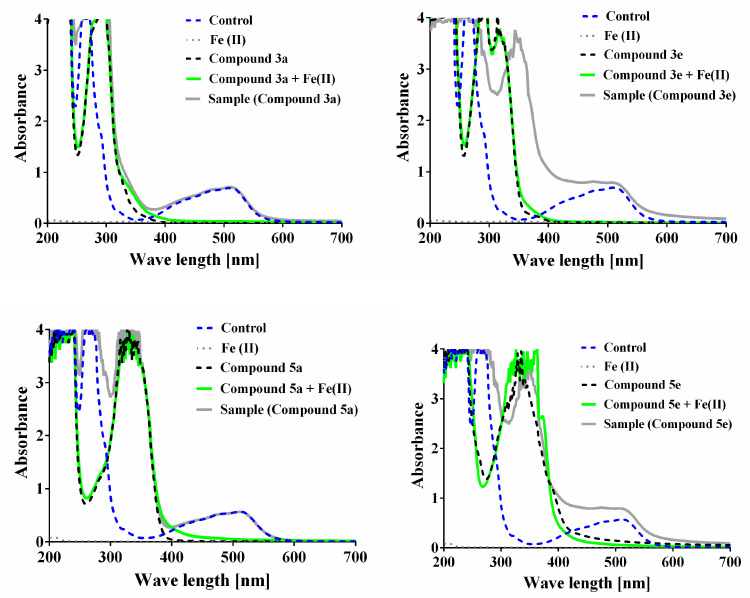
UV-vis spectra absorbance spectra of control-standard orange–red Fe(II)-1,10-phenantroline complex; the tested compounds **3a**, **3e**, **5a**, and **5e** (0.2 mM) alone and in the presence of Fe(II) (0.05 mM); and the sample solution containing 1,10-phenanthroline (0.2 mM), Fe(II) (0.05 mM), and the hydrazone derivatives (0.2 mM).

To clarify the obtained data, we also measured the absorbance spectra of the solutions containing: (i) the hydrazone derivatives alone; (ii) the hydrazones in the presence of iron; (iii) the hydrazones in the presence of iron and 1,10-phenanthroline (samples); and (iv) the ferroin complex (control) (Figure 7, see also Supplementary Materials).

Compounds IPA, **3b**, **3c**, **3d**, **3f**, **5b**, **5d**, and **5f** had statistically identical absorbance values at 515 nm, as compared to the controls. The sample solutions were orange–red, and the UV-vis spectra revealed the characteristic maximum in the 400–600 nm range for the Fe(II)–1,10-phenanthroline complex. The obtained absorbance spectra of the compounds alone and in Fe(II) presence were identical, in the range 190–1100 nm. This suggested the hydrazones were unable to interact directly with Fe(II).

The compounds **3a** and **5a** also had identical absorbance values, as compared to the controls (λ = 515 nm). In the presence of Fe(II), both compounds, as well as **5c**, had minimal changes in the absorbance spectra of the pure compounds, in the 370–420 nm range. Despite this, again, the UV-vis spectra of the samples revealed a characteristic maximum in the range 400–600 nm for the Fe(II)–1,10-phenanthroline complex, suggesting its incapacity to prevent ferroin complex formation.

Compounds **3e** and **5e** at λ = 515 nm had much higher absorbance values than the controls. The UV-vis spectra, measured in the presence of ortho-phenanthroline, the hydrazones, and Fe(II), showed changes, as compared to the ferroin complex, suggesting another structure’s formation.

### 3.6. Superoxide Radicals Scavenging Activity

**Figure 8:**
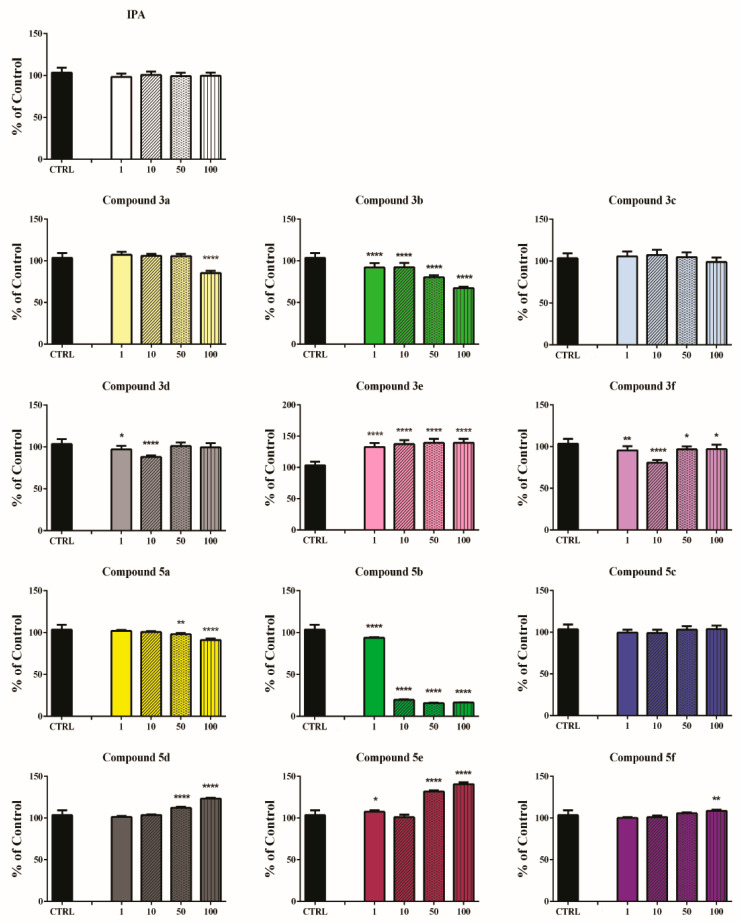
Superoxide-radical-scavenging activity assay in xanthine-oxidase model system. Data are presented as means ± SEM from three independent measurements. * *p* < 0.05, ** *p* < 0.01, **** *p* < 0.0001, vs. control. One-way analysis of variance with Dunnet’s post hoc test were used.

The potential capability of the investigated compounds to prevent the superoxide-anion radical-induced NBT reduction was investigated spectrophotometrically by xanthine-oxidase assay. The assay was carried out at the final concentration of the tested compounds, at 1, 10, 50, and 100 µM. It should be noted that compounds IPA, **3c**, and **5c** did not influence the estimated absorbance ratio at any of the tested concentrations (Figure 8), suggesting no capability to scavenge the superoxide-anion radical or to influence XO enzyme function.

In the presence of **3e**, **5d**, **5e**, and **5f**, we observed a statistically significant increase in the absorbance ratio parameter. In the samples containing **3e**, the observed effect was not concentration dependent, and the calculated parameter was approximately 135%. The presence of the other three compounds had a concentration-dependent impact on the system: the higher the hydrazones concentration, the more the absorbance ratio increased. At 100 µM, a minimal increase of 8%, as compared to the controls, was observed for **5f**. For the other two hydrazones, **5d** and **5e**, the increase, as compared to the controls, was more significant at 23% and 40%, respectively.

In the samples containing **3a**, **3d**, **3f**, and **5a** a moderate decrease in the %, as compared to the controls, was observed at the highest tested concentration. The detected decrease was less than 15%. The most prospective derivatives in this system were the 3,4-dihydroxy compounds. Both compounds had decreased NBT reduction in a concentration-dependent manner. In the presence of **3b** at a concentration of 100 µM, the reduction was more than 40%, and **5b** managed to decrease it to 20%, at a 10-times lower concentration.

### 3.7. hMAO-B enzyme Inhibition

**Figure 9:**
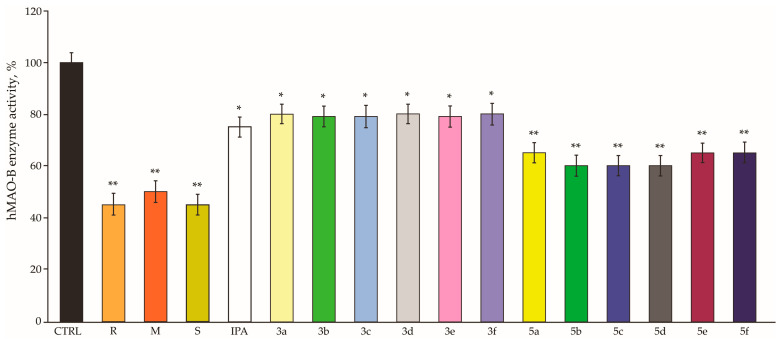
Effects of the compounds, IPA, rasagiline (R), melatonin (M) and selegiline (S), administered alone at a concentration of 1 µM, on hMAO-B enzyme activity. * *p* < 0.05; ** *p* < 0.01 vs. controls (pure hMAO-B).

The degree of hMAO-B enzyme activity inhibition by the compounds (1 µM) is depicted in Figure 9. All compounds revealed statistically significant hMAO-B inhibition. The 5-MeO Ind group performed better than the IPA group, as compared to the controls (pure hMAO-B), reducing the enzyme activity by approx. 35–40% and 20%, respectively. However, rasagiline (R), melatonin (M), and selegiline (S) performed better, inhibiting hMAO-B by approx. 50–55%. The reference IPA managed to reduce the enzyme activity by 25%.

**Figure 10:**
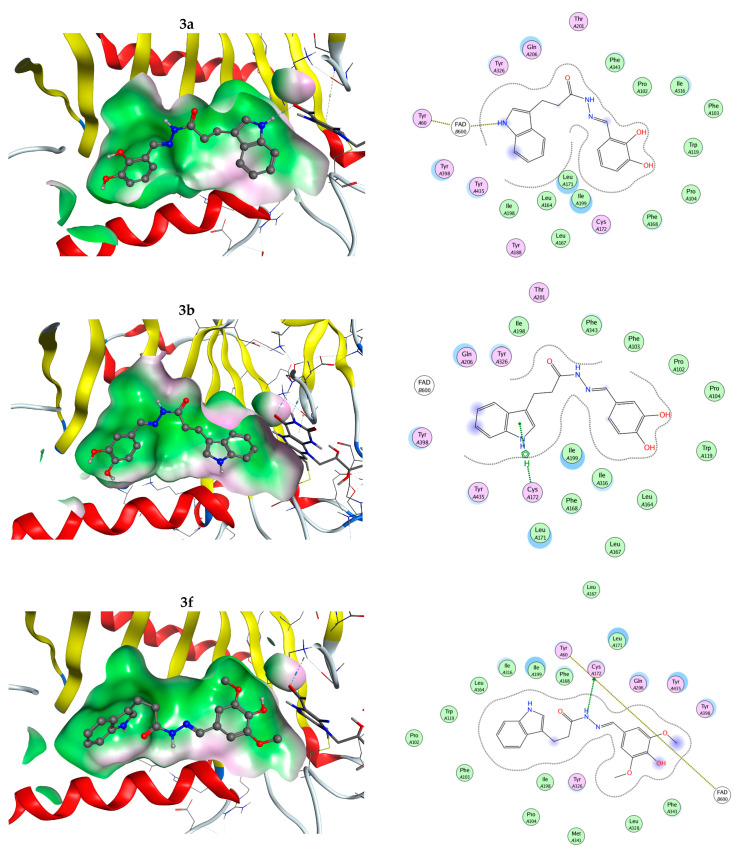

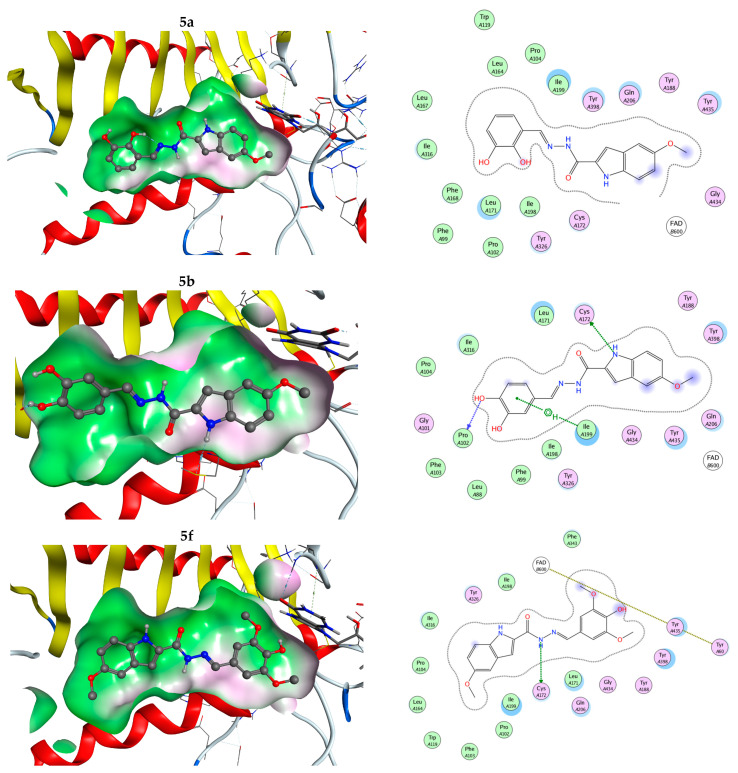
Docking poses of selected ligands in the MAO-B cavity (left part) and schematic representation of the neighboring amino acid residues and interactions (right part); the components and interactions are described by the following color patterns: proximity contour—black dotted line; polar amino acids—pink; lipophilic amino acids—green; basic amino acids—blue; acidic amino acids—red; hydrogen bond interactions—blue arrows; lipophilic interactions—green dotted lines; docked structures—represented by balls and sticks; and FAD—represented by sticks.

The possible interactions of the compounds of the two series with the MAO-B enzyme were studied by molecular docking with a model prepared from the published crystalline structure of the recombinant human MAO-B, in a complex with zonisamide [55]. The modeling results showed that all the synthesized inhibitors resembled similar planar conformations, which fit well in the flat hydrophobic cavity of the MAO-B active site (Figure 10). The interactions of selected ligands in the MAO-B active pocket are illustrated in Figure 9. As shown in the figure, the molecules occupied both parts of the specific enzyme’s active site, the substrate and the active part.

Compounds **5f** and **5e** were the first and third in the docking score as both parts of series II. Their position in the active site of the MAO-B was exactly between the 2 Tyr residues, Tyr398 and Tyr435, that form the aromatic cage. It was crucial for positioning the natural substrates at the right position for the FAD co-factor [64,65,66,67], thus the position of our inhibitors mimicked the optimal position of the attacked bond at the active site of the enzyme.

Most of the newly synthesized compounds were optimal in size and conformation, adapting to the flat active site cavity with no steric hindrances, as demonstrated by the appropriate position of the 2 gate residues, Ile199 and Leu171 (with either Ile199 or neighbor of Leu171, Cys172, involved in the H-bonding with our inhibitors). Therefore, the promising experimental results of in vitro systems had a legitimate explanation for being ideally sterically fitting in the enzyme’s active site, a feature identified by our docking procedure.

### 3.8. BBB Model Permeability

**Figure 11:**
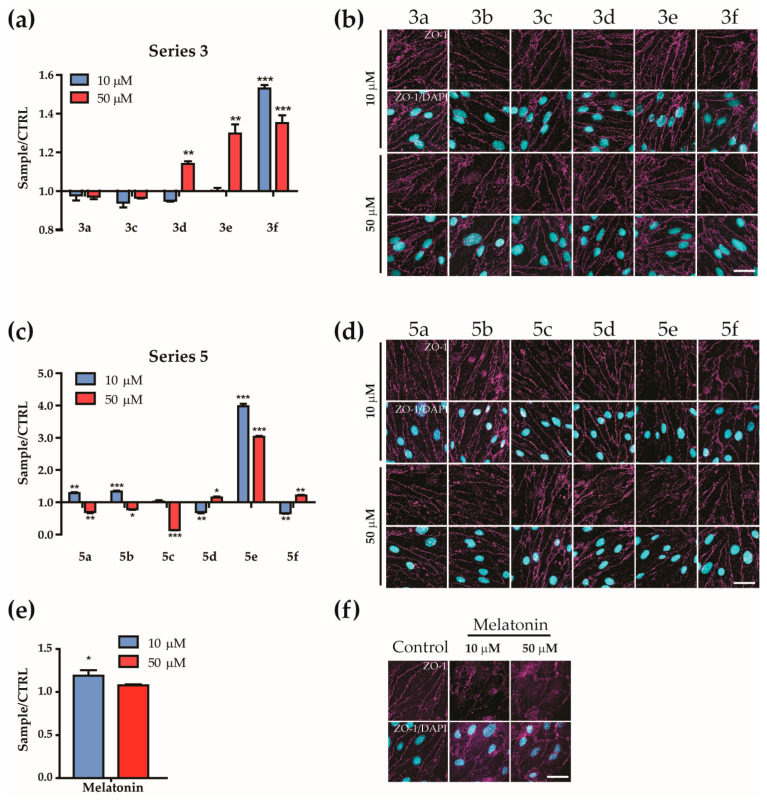
The bEnd3 blood–brain barrier model: Permeability of bEnd3 monolayer, treated with compounds **3a–f** (**a**), compounds **5a–f**, (**c**) or melatonin, (**e**) vs. control. The normalized control value was used as a baseline. Bars above the baseline indicate an increase in permeability; bars under the baseline indicate a decrease. Immunofluorescence of ZO-1 (tight junctions) of bEnd3 in vitro cell monolayer model, treated with series I (**b**), series II, (**d**) or melatonin (**f**). The cells were treated with 10 or 50 µM of the tested and reference compounds. ZO-1—magenta; DAPI (nucleus)—cyan. Scale bar—20 µm. Data are presented as means ± SD. * *p* < 0.05, ** *p* < 0.01, *** *p* < 0.001, vs. control. One-way analysis of variance and Dunnet’s post hoc test were used.

To further explore the neuroprotective effect of the compounds, we tested their ability to influence the BBB and its permeability, utilizing a static BBB model with brain microvascular endothelial cell line bEnd3 [36], as it would be essential for the compounds to pass the BBB and protect the neuronal cells. In addition to their endothelial origin, the bEnd3 cells were strongly connected by specific proteins (ZO-1, claudin-5) forming tight junctions (TJs). This was crucial for preventing unregulated transcellular transport and sustaining the apical–basal polarity of the monolayer. Here, we determined any changes in the permeability of the BBB by imitating an endothelial barrier via a static BBB model of bEnd3 cells (Figure 11a,c,e). This allowed us to determine the possibility of drug passage. It should be noted, that even though drug passage was desirable, any irreversible modifications of the cell monolayer and the TJs were considered threatening. Therefore, in parallel to the permeability tests, the bEnd3 cells were stained for TJ-formation and ZO-1 distribution (Figure 11b,d,f).

The BBB model was treated for 90 min with 10 and 50 µM concentrations of the compounds. From series I, only **3d**, **3e**, and **3f** increased the permeability at a 50 µM concentration. The compound **3f** appeared to have the highest influence, causing a 0.5-times increase in the permeability at 10 µM (Figure 11a), as compared to the ZO-1 distribution, i.e., TJs remained intact for all compounds. For series II, a significant increase in permeability was observed for **5e**, by 3 to 4 times. However, it appears to disrupt TJs, as shown in Figure 11d, where the cellular boundaries are not very clear, indicating that **5e** was not a good choice for future administration. Compounds **5a**, **5b**, and **5c** reduced the permeability at 50 µM, whereas **5d** and **5f** decreased it at 10 µM. The passage through the BBB model was induced by 10 µM concentrations of **5a** and **5b**, without affecting the TJs. Melatonin did not influence the permeability of the monolayer.

## 4. Discussion

Two series of indole derivatives, structurally related to indole-3-propionic acid and 5-methoxy-indole carboxylic acid, were synthesized by linking the heterocyclic indole moiety with hydroxy/methoxy-substituted phenyl fragments through a hydrazone chain. The reaction pathway involved a conversion via ester- and hydrazide-intermediates, resulting in high yields and purity of the target compounds.

Establishing a safety profile was an essential step in the initial characterization of the drug candidates. The properties of the erythrocyte cell membranes were extensively studied and may serve as a useful model system for assessing toxicity. Moreover, the in vitro hemolysis test could be correlated with the in vivo toxicity of the newly synthesized compounds. Favorably, all indole hybrids of both series I and II showed no or very low hemolytic effects below the acceptable hemolytic threshold, according to the ISO standards, indicating good safety and hemocompatibility. The effect on the cell viability of the human neuroblastoma SH-SY5Y cell line and the immortalized mouse-brain microvascular-endothelial cell line, bEnd3, also confirmed that the newly derived compounds did not exhibit any significant cytotoxic effects. The SH-SY5Y cell line is a well-known model used in neuroscience to study the development and the function of the nervous system, as well as for the study of neurogenerative disorders, such as Alzheimer’s and Parkinson’s diseases. The SH-SY5Y cells have dopamine-β-hydroxylase and tyrosine-hydroxylase activities [68,69] and high levels of axonally localized TAU protein [70]. To reach the neurons, substances must first cross the BBB without disrupting the barrier, since that could lead to neurological damage [71]. Therefore, the cytotoxic effects of the compounds on the endothelial cells were also evaluated using the immortalized mouse-brain microvascular-endothelial cell lin, bEnd3, which had been shown to offer a close representation of in vivo BBB characteristics [72,73,74,75]. Despite some differences in the overall response, this model confirmed the low toxicity of the compounds and emphasized the importance of such parallel analyses in selecting promising agents for future pharmacological studies. In a third model, using freshly isolated rat-brain synaptosomes, the hybrids showed statistically significant effects on the functional-metabolic status of the isolated synaptosomes, with **5a–f** showing lower toxicity than the IPA derivatives **3a–f**.

The neuroprotective effects of series I (**3a–f**) on H_2_O_2_-induced oxidative stress in SH-SY5Y cells exceeded those of the 3 reference compounds IPA, melatonin, and rasagiline. The compounds of series II (**5a–f**) also demonstrated excellent neuroprotection, performing better than the references at the lower concentrations of 1 and 10 µM. In this oxidative-stress cellular model, the toxic substance H_2_O_2_ depleted cellular antioxidants and produced reactive hydroxyl radicals that caused DNA damage; the oxidation of proteins and lipids; and activated numerous signaling pathways that could lead to inflammation and other cellular damage. In the model of 6-hydroxydopamine-induced oxidative stress on isolated synaptosomes, all compounds exhibited statistically significant neuroprotective effects, as shown by the SV and GSH levels, but this was less pronounced than in the reference compounds IPA, rasagiline, and melatonin. The strongest neuroprotection was observed with the 2,3-dihydroxy, 2-hydroxy-4-methoxy, and syringaldehyde derivatives from series II.

Over the years, several different alternative methods have been developed for the initiation of iron-induced oxidative damage, including (i) H_2_O_2_/Fe(III)–EDTA/ascorbic acid; (ii) an H_2_O_2_/Fe(III)–EDTA variant; (iii) Fe(III)–EDTA/ascorbic acid; and (iv) Fe(III)–EDTA and Fe(II) [61,62,63,76]. In the current case, the protocol that initiated oxidative damage only with ferrous ions was chosen due to its simplicity. In this protocol, the generation of many secondary products, including five deoxyribose radicals, only one of which could react with thiobarbituric acid [63], was avoided. The possibility of ascorbic-acid-induced amplification of iron’s oxidative potential and metal-ion-induced lipid peroxidation had also been eliminated. If an antioxidant effect was observed in the systems under investigation, it could, therefore, be attributed either to radical-scavenging properties, chelation activities, or both. The capacity of compounds to increase the iron-induced oxidative damage suggested their potential toxicity, the possibility of essential cellular components being altered, and the subsequent disruption of their original functions. Additionally, it could be connected to the production of toxic breakdown products that have been shown to cause mutations [77].

All derivatives from both series suppressed the iron-induced lipid peroxidation. In the deoxyribose-degradation assay, the compounds **3d** and **3e** increased the iron-induced oxidative damage. The obtained results for the chelation test and the data from both systems, including the iron-induced lipid peroxidation and the deoxyribose degradation, suggested that, in cases where the antioxidant effect had been observed, it could be attributed to the direct antiradical properties and could not be explained by any chelation ability. The newly synthesized hybrids possessed versatile antioxidant properties, showing a certain relationship with their structural characteristics. The two 2,3-dihydroxy compounds from both series (**3a** and **5a**) revealed the strongest inhibition against LP and deoxyribose degradation. Even though in the lecithin-containing system, among the IPA derivatives, the structural modifications in the phenyl fragment led to fewer expressed differences in the observed effects, with the analogs having catechol functions (the 2,3- and 3,4-dihydroxy derivatives) and those containing a syringaldehyde residue bearing a high potential as antioxidants in the systems.

In the evaluation of the potency of the tested hydrazones, both of the 3,4-dihydroxy derivatives, **3b** and **5b**, showed a promising effect for decreasing the amount of generated superoxide-anion radicals (O_2_^−●^) using the enzymatic in vitro system xanthine–xanthine-oxidase. In the presence of **3b** (100 µM), the reduction was more than 40%, as compared to the controls, and **5b** (10 µM) managed to decrease the effect to 20%. The selected system was especially important considering the major O_2_^−●^ generated in the oxidative phosphorylation. Moreover, it was consistent with data, revealing significantly higher XO activities in PD patients, as compared to healthy controls [78]. Although it was not very reactive, O_2_^−●^ could easily interact with other molecules to form more potent and harmful secondary ROS. Normally, O_2_^−●^ could be efficiently detoxified by superoxide dismutases (SODs), which have been associated with H_2_O_2_ release. This could then generate hydroxyl ions in the ferrous-ion-catalyzed breakdown. Superoxide anion is also produced during dopamine oxidation at its electron-rich catechol moiety [79].

In addition to neuroprotective and radical-scavenging properties, all compounds showed a statistically significant inhibition of hMAO-B, with series II being more active than series I. MAO-B is involved in dopamine oxidation and concurrent degradation, releasing hydrogen peroxide (H_2_O_2_) in the process. Therefore, PD patients have been affected not only by the loss of dopaminergic neurons and dopamine production [80,81] but also by the free radicals generated by MAO-B. As MAO-B’s activity increases with age, the brain is under a combined attack from dopamine depletion, oxidative stress, ammonia, and toxic 3,4-dihydroxyphenylacetaldehyde (DOPAL) [82,83,84]. Furthermore, Chamoli et al. used a MAO-B-overexpressed murine model, in which astrocytes developed senescence-associated behavior, to suggest that the affected astrocytes could negatively impact nearby neurons [85]. When MAO-B oxidized 6-OHDA, free radicals were generated as by- products, which could, in turn, have neurologically damaging effects [85]. These ROS could be attached via reduced glutathione (GSH), leading to its oxidation into GSSG and the subsequent depletion of GSH. Therefore, reduced levels of GSH corresponded to high levels of oxidative stress and a high 6-OHDA metabolic rate. In the synaptosomal model, all the compounds were able to inhibit hMAO-B, decreasing the available MAO-B in the test system. As expected, the enhanced MAO-B inhibition, as observed in the compounds in series II, led to a higher synaptosomal viability and higher GSH levels. The ligand–enzyme interaction with compounds from series II mimicked the natural substrate, as their position was in the aromatic cage of the MAO-B active site.

The vascular barrier between the central nervous system (CNS) and the blood circulation, known as a blood–brain barrier (BBB), comprises a semipermeable endothelial monolayer that supplies neurons and glial cells with nutrients and hormones. By maintaining its integrity, the endothelium protects the brain against environmental toxins and other blood-borne stressors. However, in some pathological conditions, the BBB becomes permeable and fails to preserve the brain’s homeostasis, allowing certain chemicals to penetrate through intra- and intercellular pathways. Such BBB disruptions have also been observed in PD patients but were not sufficiently studied [86]. Given that drug accessible to the CNS is limited by the BBB, new drugs with the potential to cross the BBB in order to treat the CNS are consistently being developed. As in vivo studies were somewhat limited, several simplified in vitro BBB models have been proposed to study BBB’s permeability [87]. Such evaluations are important not only to assess the potential of MAO-B inhibitors to manage the CNS but also to treat non-CNS diseases [88]. Currently, rasagiline is the gold-standard drug for PD treatment, as it is an irreversible MAO-B inhibitor that can cross the BBB and has anti-apoptotic effects that are independent of its MAO-B inhibition. The last property is beneficial also for non-dopaminergic neurons, the loss of which has been associated with dementia, psychosis, and apathy. Regardless of the promising trials of MAO-B inhibitors, summarized by Cheong Teo et al. in 2013 [81], there are still many side effects, supporting the inquiry for new medication causing few or well-tolerated side effects. Therefore, the neuroprotective effects of the compounds in this study were further characterized by testing their ability to influence the BBB and its permeability, utilizing a static BBB model with the mouse-brain microvascular-endothelial cell line, bEnd3. Both of the compounds exhibiting chelating properties, **3e** and **5e**, showed high BBB permeability. However, they also had the highest cytotoxicity in the bEnd3 cells, as compared to the other compounds, with **5e** even disrupting the TJs. Recently, the chelation of iron was shown to protect the BBB against mitochondrial ROS formation and apoptosis [89], suggesting that another mechanism underlay the observed negative effects of the two compounds. It was shown that, in series I, the syringaldehyde derivative **3f** increased the permeability of the monolayer to the highest extent, while the ZO-1 distribution, i.e., the TJs, remained intact for all the compounds. Meanwhile, series II showed an overall change in the permeability in a dose-dependent manner. The compounds that demonstrated good hMAO-B inhibition, as well as high antioxidant and neuroprotective activity at high concentrations, such as **5a** and **5b**, stimulated the BBB’s integrity and reduced its permeability. Interestingly, lower concentrations increased the passage through the endothelial monolayer, while maintaining the cell–cell contacts.

## 5. Conclusions

The current study evaluated the potential of new aryl-hydrazone hybrids of IPA and 5-methoxy-indole-2-carboxilic acid as neuroprotectors through various antioxidant mechanisms, as well as their ability to inhibit the MAO-B enzyme. The compounds had good safety profiles with hemolytic effects <5% at the highest concentration of 200 μM. Furthermore, their neurotoxic effects were evaluated on two different cell lines, the SH-SY5Y neuroblastoma cell line and the bEnd3 mouse-brain endothelial cell lines. The majority of the compounds had high IC_50_ values, indicating low toxicity. The compounds **3e** and **3f** had no cytotoxic effects, with IC_50_ values > 500 µM, followed by the compounds **3b** (265.3 µM), **3c** (275.2 µM), and **5f** (286.9 µM). For both cell lines, **3f** did not exhibit any cytotoxic effects. The in vitro neuroprotective effects were evaluated by H_2_O_2_-induced oxidative stress on SH-SY5Y cells, on which all the tested compounds showed highly potent activity that was more pronounced than the reference IPA, melatonin, and rasagiline. In the model of 6-OHDA-induced oxidative stress on isolated synaptosomes, all the compounds exhibited statistically significant effects by preserving the SV and GSH levels. The strongest neuroprotection was observed with the 2,3-dihydroxy, 2-hydroxy-4-methoxy, and syringaldehyde derivatives in series II. All hybrids in both series suppressed the iron-induced lipid peroxidation. Two 2,3-dihydroxy compounds from both series (**3a** and **5a**) revealed the strongest inhibition against LP and deoxyribose degradation. In the evaluation of the potency of the tested hydrazones, both of the 3,4-dihydroxy derivatives, **3b** and **5b**, showed a promising effect on decreasing the amount of generated superoxide-anion radicals. In the presence of **3b** (100 µM), the reduction was more than 40%, as compared to the controls, and **5b** (10 µM) managed to decrease the effect to 20%. In addition to neuroprotective and radical-scavenging properties, all the compounds demonstrated their statistically significant inhibition of hMAO-B, with series II being more active than series I, which was also supported by the docking studies. It was shown that the series-I syringaldehyde derivative **3f** increased the permeability of the endothelial monolayer to the highest extent in the in vitro BBB model, while the tight junctions remained intact for all the compounds. Based on the conducted studies, the combined effects of MAO-B inhibition, the reduction in OS, and the maintenance of synaptosomal viability, with the potential to pass the BBB, indicates the series-II compounds as promising candidates for PD treatment.

## Figures and Tables

**Table 1:** In vitro cytotoxicity of new benzimidazole derivatives **3a–f** and **5a–f** (IC_50_ values) on SH-SY5Y and bEnd3 cells.

Compound	SH-SY5Y Cells			bEnd3 Cells
	IC_50_ (µM)	95% Confidence Intervals	IC_50_ (µM)	95% Confidence Intervals
IPA	>500		NA	NA
**3a**	119.6	80.2 to 140.2	355.6	329.0 to 385.1
**3b**	265.3	212.3 to 289.2	352.2	305.6 to 405.6
**3c**	275.2	248.2 to 311.3	>500	
**3d**	155.9	115.7 to 219.1	367.3	361.4 to 373.5
**3e**	>500		85.5	75.6 to 96.6
**3f**	>500		>500	
**5a**	79.3	59.63 to 105.6	197.4	169.0 to 230.6
**5b**	111.1	95.31 to 128.8	157.9	131.6 to 189.6
**5c**	73.9	56.3 to 89.32	>500	
**5d**	98.2	79.4 to 111.5	352.8	325.4 to 382.6
**5e**	199.5	187.42 to 204.54	12.1	9.3 to 15.5
**5f**	286.9	235.3 to 320.1	184.0	145.9 to 232.1
Melatonin	>500		>500	
Rasagiline.HCl	>500		NA	NA

## Data Availability

Data is contained within the article.

## Supplementary Materials

The following supporting information can be downloaded at: https://www.mdpi.com/article/10.3390/antiox12040977/s1, Figure S1: ^1^H NMRspectrum of 2 (400 MHz, DMSO-d6; Figure S2: 1H NMRspectrum of (3a) (400 MHz, DMSO-d6); Figure S3: 13C NMR spectrum of 3a (151 MHz, DMSO-d6); Figure S4: 1H NMR spectrum of (3c) (600 MHz, DMSO-d6); Figure S5. 13C NMR spectrum of 3c (151 MHz, DMSO-d6); Figure S6: 1H NMRspectrum of (3d) (600 MHz, DMSO-d6); Figure S7: 13C NMR spectrum of 3d (151 MHz, DMSO-d6); Figure S8: 1H NMRspectrum of (3e) (600 MHz, DMSO-d6); Figure S9: 13C NMR spectrum of 3e (151 MHz, DMSO-d6); Figure S10: 1H NMRspectrum of (3f) (600 MHz, DMSO-d6); Figure S11: 1H NMRspectrum of 4 (600 MHz, DMSO-d6); Figure S12: 13C NMR spectrum of 4 (151 MHz, DMSO-d6); Figure S13: 1H NMRspectrum of (5a) (600 MHz, DMSO-d6); Figure S14: 13C NMR spectrum of 5a (151 MHz, DMSO-d6); Figure S15: 1H NMR spectrum of 5b (600 MHz, DMSO-d6); Figure S16: 13C NMR spectrum of 5b (151 MHz, DMSO-d6); Figure S17: 1H NMR spectrum of 5c (600 MHz, DMSO-d6); Figure S18: 13C NMR spectrum of 5c (151 MHz, DMSO-d6); Figure S19: 1H NMR spectrum of 5d (600 MHz, DMSO-d6); Figure S20: 13C NMR spectrum of 5d (151 MHz, DMSO-d6); Figure S21: 1H NMR spectrum of 5e (600 MHz, DMSO-d6); Figure S22: 13C NMR spectrum of 5e (151 MHz, DMSO-d6); Figure S23: 1H NMR spectrum of 5f (600 MHz, DMSO-d6); Figure S24: 13C NMR spectrum of 5f (151 MHz, DMSO-d6); Figure S25: ATR-IR spectrum of 3a; Figure S26: ATR-IR spectrum of 3b; Figure S27: ATR-IR spectrum of 3c; Figure S28: ATR-IR spectrum of 3d; Figure S29: ATR-IR spectrum of 3e; Figure S30: ATR-IR spectrum of 3f; Figure S31: ATR-IR spectrum of 5a; Figure S32: ATR-IR spectrum of 5b; Figure S33: ATR-IR spectrum of 5c; Figure S34: ATR-IR spectrum of 5d; Figure S35: ATR-IR spectrum of 5e; Figure S36: ATR-IR spectrum of 5f; Figure S37: UV-Vis spectra absorbance spectra of the Control; Figure S38: UV spectra of 3a in buffers with pH 2, 4.5, 7.4 and 8.5 measured at the initial point (0 min) and after 1, 2, 3 and 24 h; Figure S39: UV spectra of 3d in buffers with pH 2, 4.5, 7.4 and 8.5 measured at the initial point (0 min) and after 1, 2, 3 and 24 h ; Figure S40: UV spectra of 5d in buffers with pH 2, 4.5, 7.4 and 8.5 measured at the initial point (0 min) and after 1, 2, 3 and 24 h; Figure S41: UV spectra of 5e in buffers with pH 2, 4.5, 7.4 and 8.5 measured at the initial point (0 min) and after 1, 2, 3 and 24 h.

## Appendix A

Supplementary data: ^1^H NMR and ^13^C NMR spectra of the synthesized compounds; ATR-IR spectra of the synthesized compounds; UV-vis absorbance spectra of control-standard orange–red Fe(II)-1,10 phenantroline complex; the tested hydrazone compounds; UV absorbance spectra at different pH measured from 0, 1, 2, 3, and 24 h.
