# Supplementary material for: New Indole-3-Propionic Acid and 5-Methoxy-Indole Carboxylic Acid Derived Hydrazone Hybrids as Multifunctional Neuroprotectors
Source: Antioxidants (Basel). 2023 Apr 21;12(4):977. doi: 10.3390/antiox12040977 (PMC10135567; doi:10.3390/antiox12040977)
Supplement: Supplementary file 1 [file antioxidants-12-00977-s001.zip › antioxidants-2262553-tex.zip!/Definitions/antioxidants-2262553-g002.pdf]

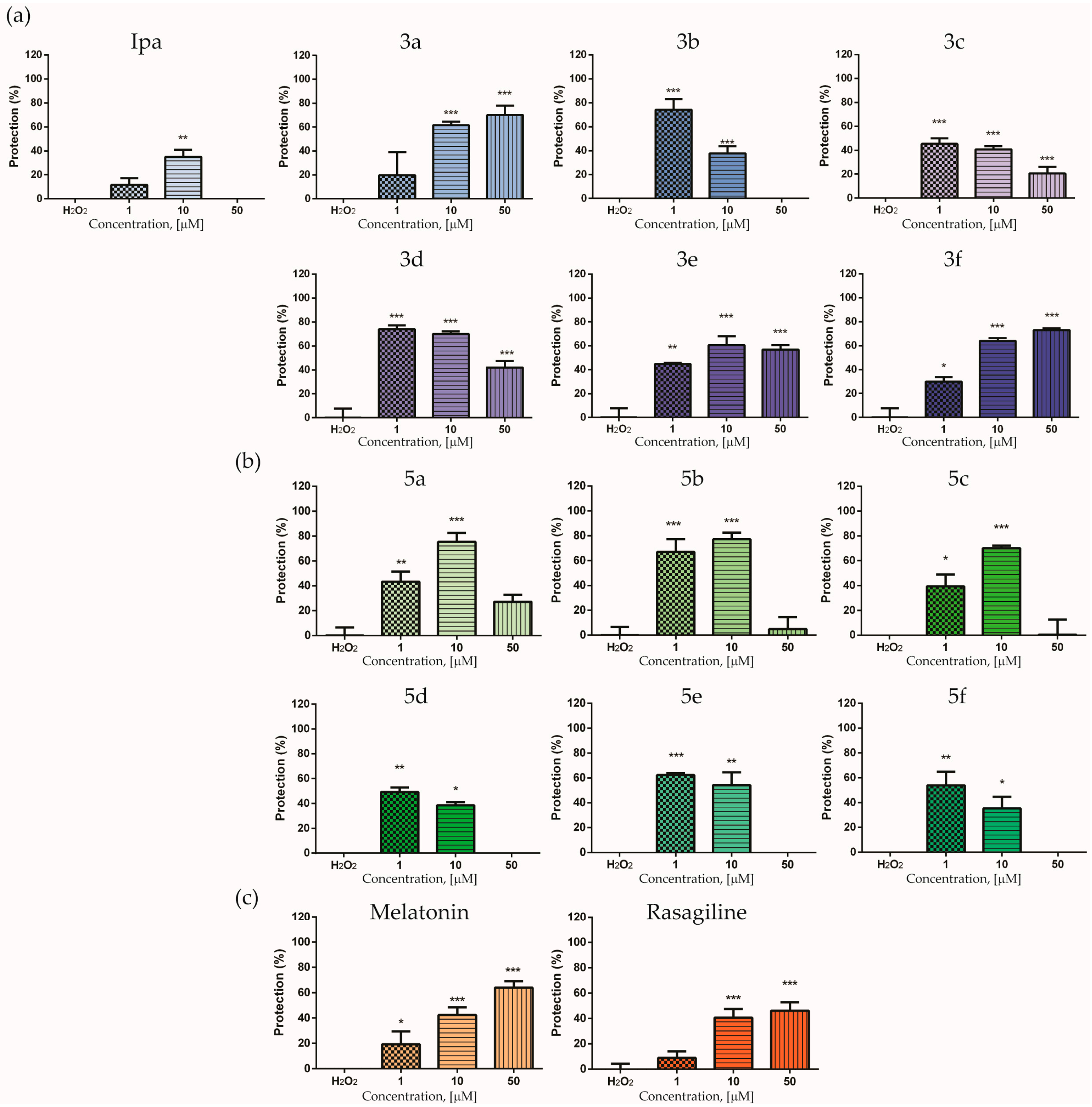

(a)
Ipa
3a
3b
3c
3d
3e
3f
(b)
5a
5b
5c
5d
5e
5f
(c)
Melatonin
Rasagiline
Protection (%)
Concentration, [µM]
H2O2
1
10
50
