# Supplementary material for: New Indole-3-Propionic Acid and 5-Methoxy-Indole Carboxylic Acid Derived Hydrazone Hybrids as Multifunctional Neuroprotectors
Source: Antioxidants (Basel). 2023 Apr 21;12(4):977. doi: 10.3390/antiox12040977 (PMC10135567; doi:10.3390/antiox12040977)
Supplement: Supplementary file 1 [file antioxidants-12-00977-s001.zip › antioxidants-2262553-tex.zip!/Definitions/antioxidants-2262553-g003.pdf]

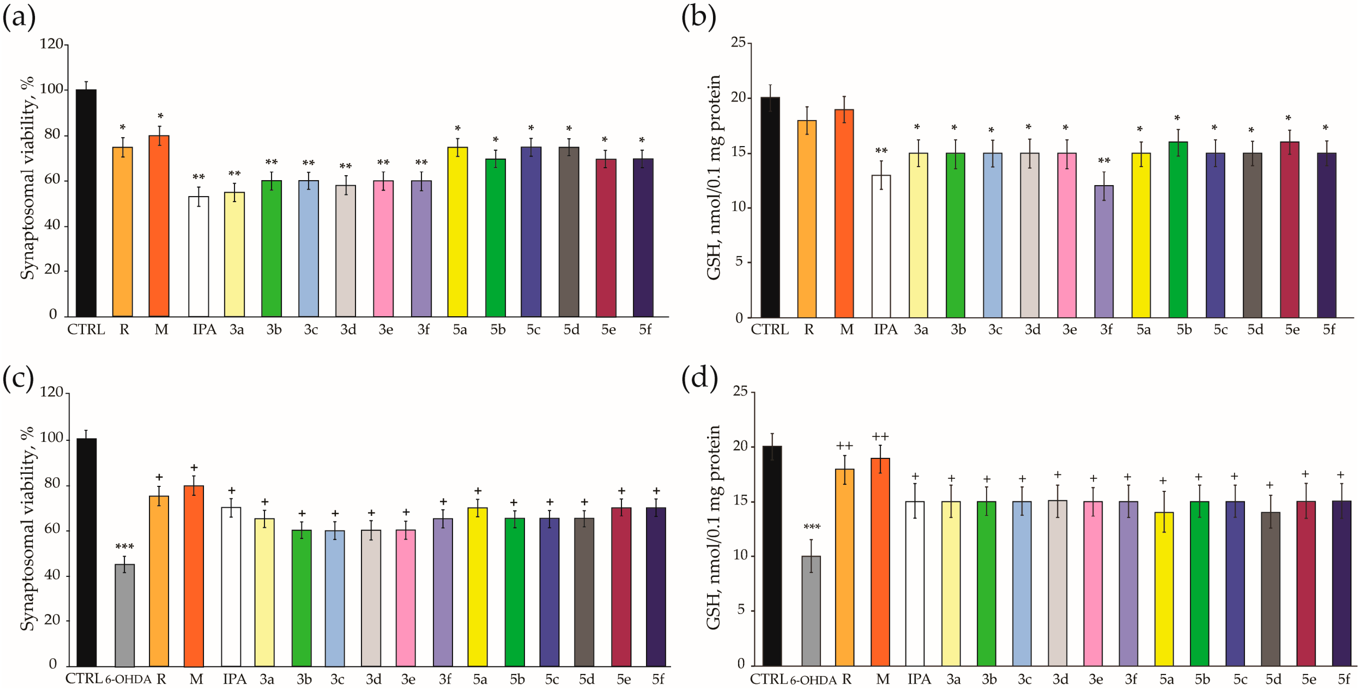
(a)
Synaptosomal viability, %
0
20
40
60
80
100
120
CTRL R M IPA 3a 3b 3c 3d 3e 3f 5a 5b 5c 5d 5e 5f
(b)
GSH, nmol/0.1 mg protein
0
5
10
15
20
25
CTRL R M IPA 3a 3b 3c 3d 3e 3f 5a 5b 5c 5d 5e 5f
(c)
Synaptosomal viability, %
0
20
40
60
80
100
120
CTRL 6-OHDA R M IPA 3a 3b 3c 3d 3e 3f 5a 5b 5c 5d 5e 5f
(d)
GSH, nmol/0.1 mg protein
0
5
10
15
20
25
CTRL 6-OHDA R M IPA 3a 3b 3c 3d 3e 3f 5a 5b 5c 5d 5e 5f
