# Supplementary material for: New Indole-3-Propionic Acid and 5-Methoxy-Indole Carboxylic Acid Derived Hydrazone Hybrids as Multifunctional Neuroprotectors
Source: Antioxidants (Basel). 2023 Apr 21;12(4):977. doi: 10.3390/antiox12040977 (PMC10135567; doi:10.3390/antiox12040977)
Supplement: Supplementary file 1 [file antioxidants-12-00977-s001.zip › antioxidants-2262553-tex.zip!/Definitions/antioxidants-2262553-g010a.pdf]

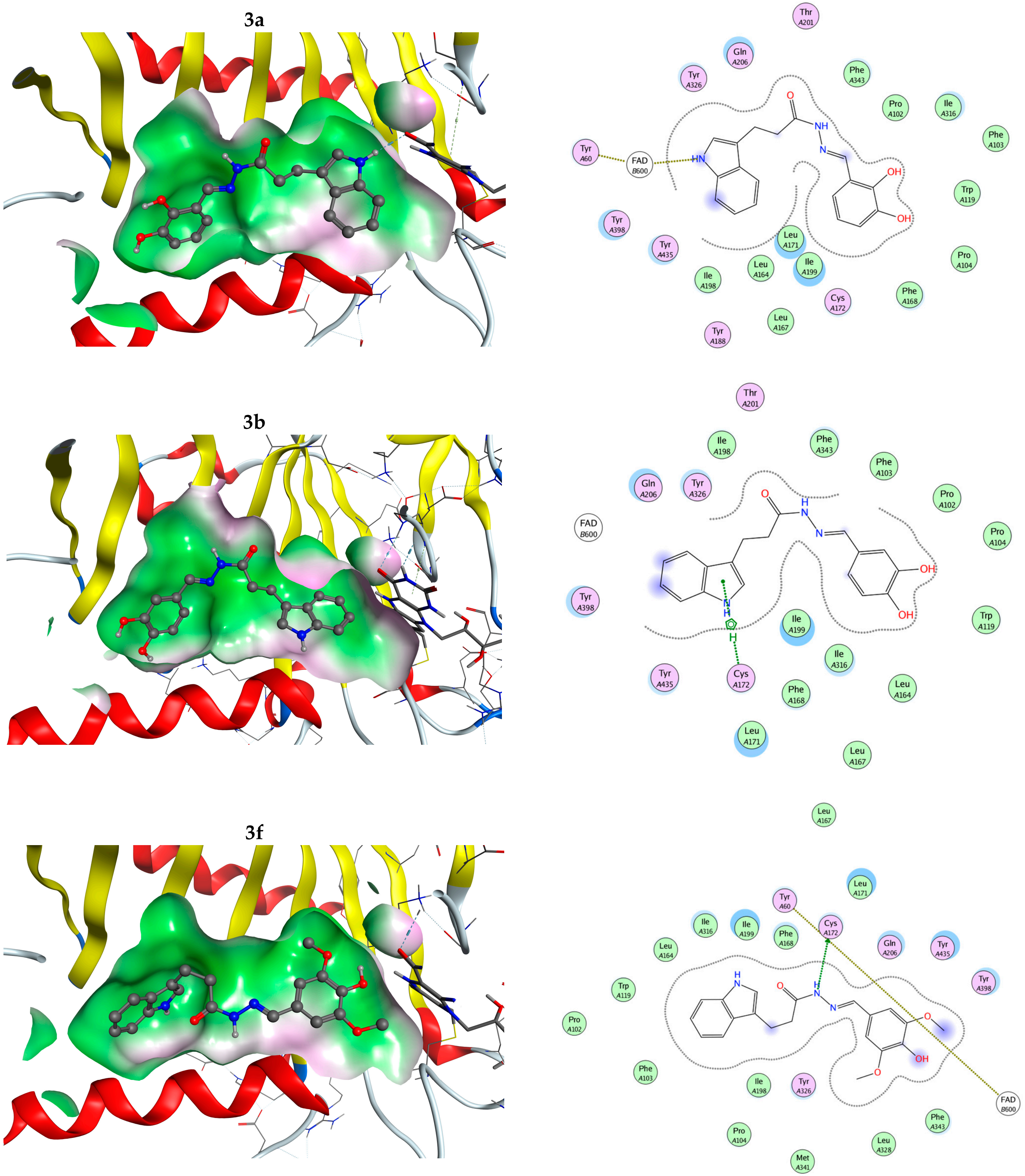
3a
Thr A201
Gln A206
Tyr A326
Phe A343
Pro A102
Ile A316
Phe A103
Tyr A60
FAD B600
HN
O
NH
N
OH
OH
Trp A119
Tyr A398
Tyr A435
Leu A171
Leu A164
Ile A199
Ile A198
Pro A104
Cys A172
Phe A168
Leu A167
Tyr A188
3b
Thr A201
Ile A198
Phe A343
Phe A103
Gln A206
Tyr A326
Pro A102
FAD B600
Pro A104
O
H
N
N
OH
OH
Tyr A398
N
H
H
Ile A199
Trp A119
Ile A316
Tyr A435
Cys A172
Phe A168
Leu A164
Leu A171
Leu A167
3f
Leu A167
Leu A171
Tyr A60
Cys A172
Ile A316
Ile A199
Phe A168
Gln A206
Tyr A435
Leu A164
Tyr A398
Trp A119
H
N
O
H
N
N
O
OH
O
Pro A102
FAD B600
Phe A103
Ile A198
Tyr A326
Phe A343
Pro A104
Leu A328
Met A341
