# Supplementary material for: New Indole-3-Propionic Acid and 5-Methoxy-Indole Carboxylic Acid Derived Hydrazone Hybrids as Multifunctional Neuroprotectors
Source: Antioxidants (Basel). 2023 Apr 21;12(4):977. doi: 10.3390/antiox12040977 (PMC10135567; doi:10.3390/antiox12040977)
Supplement: Supplementary file 1 [file antioxidants-12-00977-s001.zip › antioxidants-2262553-tex.zip!/Definitions/antioxidants-2262553-g010b.pdf]

5a

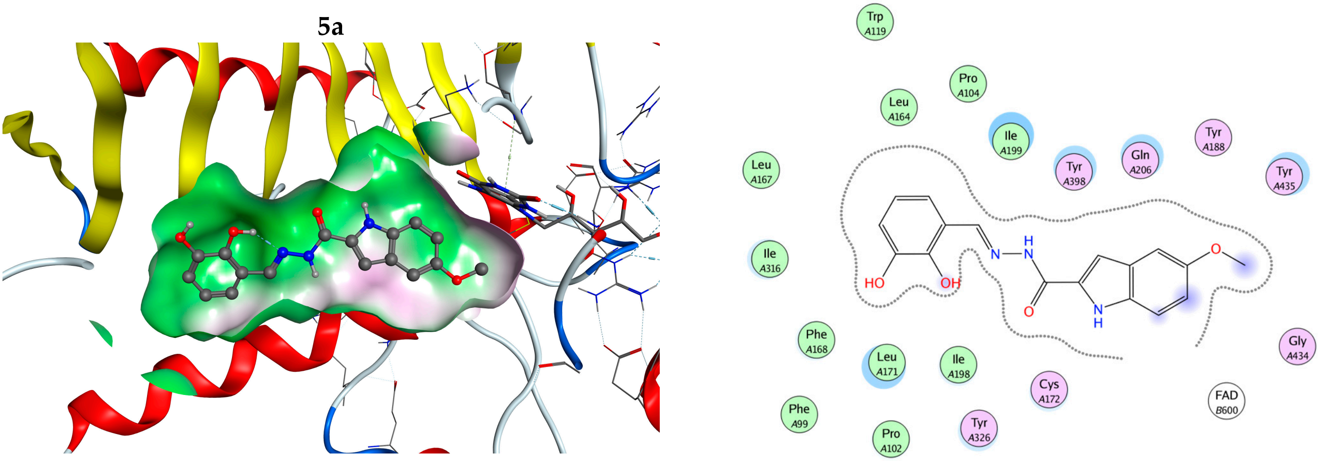

Trp A119
Pro A104
Leu A164
Ile A199
Tyr A398
Gln A206
Tyr A188
Tyr A435
Leu A167
Ile A316
HO
OH
N
N
H
O
N
H
O
Phe A168
Leu A171
Ile A198
Cys A172
Gly A434
FAD B600
Phe A99
Pro A102
Tyr A326

5b

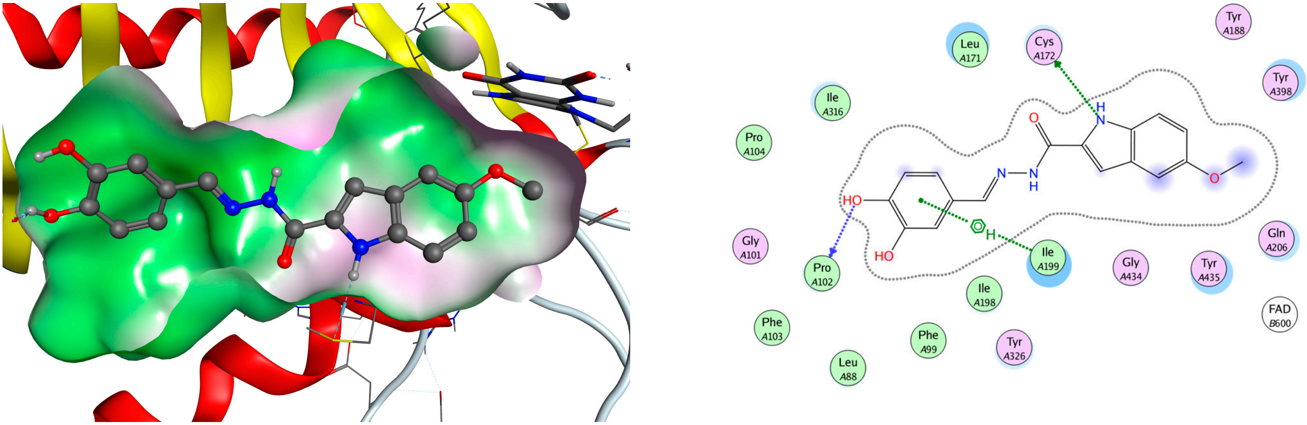

Tyr A188
Leu A171
Cys A172
Tyr A398
Ile A316
Pro A104
H
N
O
N
N
H
O
HO
HO
H
Gly A101
Pro A102
Ile A199
Gly A434
Tyr A435
Gln A206
Ile A198
FAD B600
Phe A103
Phe A99
Tyr A326
Leu A88

5f

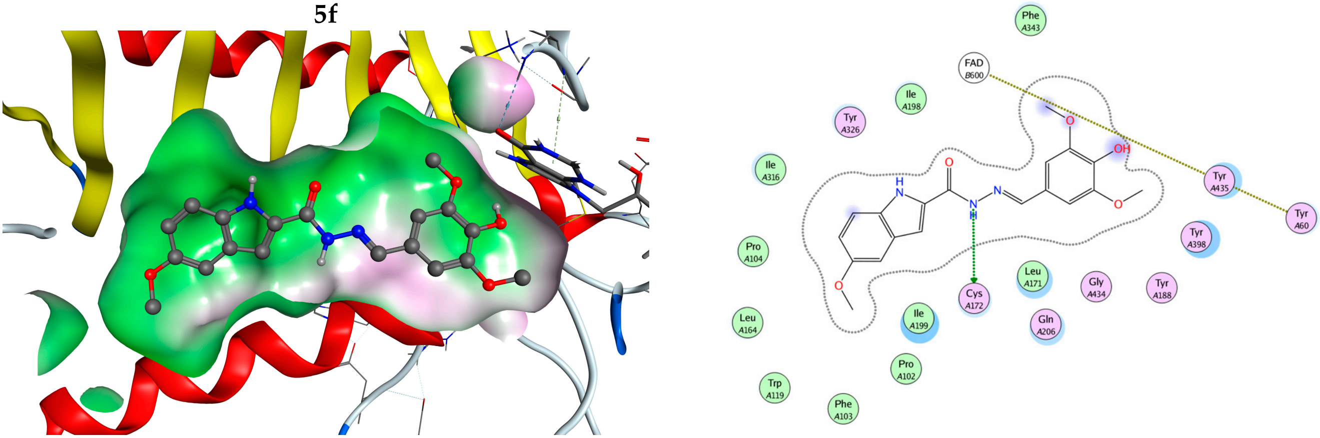

Phe A343
FAD B600
Ile A198
Tyr A326
O
OH
Ile A316
H
N
O
N
H
N
O
Tyr A435
Tyr A460
Tyr A398
Pro A104
O
Leu A171
Gly A434
Tyr A188
Cys A172
Leu A164
Ile A199
Gln A206
Pro A102
Trp A119
Phe A103
