# Supplementary material for: New Indole-3-Propionic Acid and 5-Methoxy-Indole Carboxylic Acid Derived Hydrazone Hybrids as Multifunctional Neuroprotectors
Source: Antioxidants (Basel). 2023 Apr 21;12(4):977. doi: 10.3390/antiox12040977 (PMC10135567; doi:10.3390/antiox12040977)
Supplement: Supplementary file 1 [file antioxidants-12-00977-s001.zip › antioxidants-2262553-tex.zip!/Definitions/antioxidants-2262553-g011.pdf]

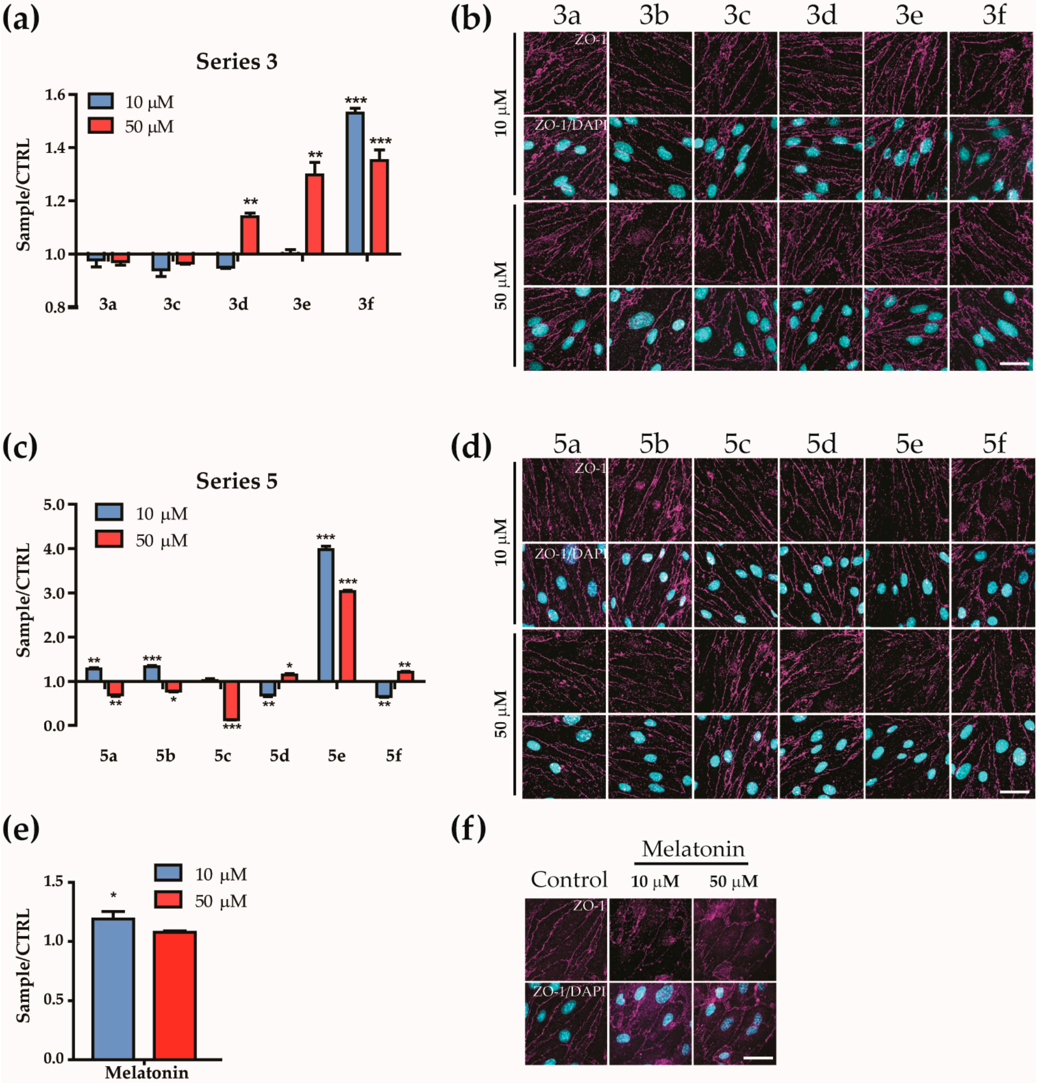
(a)
Series 3
10 μM
50 μM
Sample/CTRL
1.6
1.4
1.2
1.0
0.8
***
***
**
**
3a
3c
3d
3e
3f
(b)
3a 3b 3c 3d 3e 3f
ZO-1
ZO-1/DAPI
10 μM
50 μM
(c)
Series 5
10 μM
50 μM
Sample/CTRL
5.0
4.0
3.0
2.0
1.0
0.0
***
***
**
**
***
**
*
**
*
***
**
**
5a
5b
5c
5d
5e
5f
(d)
5a 5b 5c 5d 5e 5f
ZO-1
ZO-1/DAPI
10 μM
50 μM
(e)
10 μM
50 μM
Sample/CTRL
1.5
1.0
0.5
0.0
*
Melatonin
(f)
Melatonin
Control
10 μM
50 μM
ZO-1
ZO-1/DAPI
