# Supplementary material for: New Indole-3-Propionic Acid and 5-Methoxy-Indole Carboxylic Acid Derived Hydrazone Hybrids as Multifunctional Neuroprotectors
Source: Antioxidants (Basel). 2023 Apr 21;12(4):977. doi: 10.3390/antiox12040977 (PMC10135567; doi:10.3390/antiox12040977)
Supplement: Supplementary file 1 [file antioxidants-12-00977-s001.zip › antioxidants-2262553-tex.zip!/Definitions/logo-ccby-eps-converted-to.pdf]

CC BY
