# Supplementary material for: New Indole-3-Propionic Acid and 5-Methoxy-Indole Carboxylic Acid Derived Hydrazone Hybrids as Multifunctional Neuroprotectors
Source: Antioxidants (Basel). 2023 Apr 21;12(4):977. doi: 10.3390/antiox12040977 (PMC10135567; doi:10.3390/antiox12040977)
Supplement: Supplementary file 1 [file antioxidants-12-00977-s001.zip › antioxidants-2262553-supplementary.zip!/antioxidants-2262553-supplementary.pdf]

Supplementary material

New Indole-3-Propionic Acid and 5-Methoxy-Indole Carboxylic Acid Derived Hydrazone Hybrids as Multifunctional Neuroprotectors

Neda Anastassova [1,*], Denitsa Stefanova [2], Nadya Hristova-Avakumova [3], Irina Georgieva [4], Magdalena Kondeva-Burdina [2], Miroslav Rangelov [1], Nadezhda Todorova [5], Rumiana Tzoneva [4] and Denitsa Yancheva [1,*]

[1] Institute of Organic Chemistry with Centre of Phytochemistry, Bulgarian Academy of Sciences, Acad. G. Bonchev Str., Building 9, 1113 Sofia, Bulgaria
[2] Laboratory of Drug Metabolism and Drug Toxicity, Department of Pharmacology, Pharmacotherapy and Toxicology, Faculty of Pharmacy, Medical University-Sofia, 2 Dunav Str., 1000 Sofia, Bulgaria
[3] Department of Medical Physics and Biophysics, Faculty of Medicine, Medical University of Sofia, 2 Zdrave Str.,1431 Sofia, Bulgaria
[4] Laboratory of Transmembrane Signaling, Institute of Biophysics and Biomedical Engineering, Bulgarian Academy of Sciences, Acad. G. Bonchev Str., Block 21, 1113 Sofia, Bulgaria
[5] Institute of Biodiversity and Ecosystem Research, Bulgarian Academy of Sciences, 2 Gagarin Str., 1113 Sofia, Bulgaria
* Correspondence: neda.anastassova@orgchm.bas.bg (N.A.); denitsa.pantaleeva@orgchm.bas.bg (D.Y.)

Contents

1. 1H NMR and13C NMR spectra of the synthesized compounds
2. ATR-IR spectra of the synthesized compouns
3. UV-Vis spectra absorbance spectra of the: control - standard orange-red Fe(II) – 1,10 Phenantroline complex; the tested hydrazone compounds
4. UV spectra of selected compounds in buffers with pH 2, 4.5, 7.4 and 8.5 measured at the initial point (0 min) and after 1, 2, 3 and 24 h

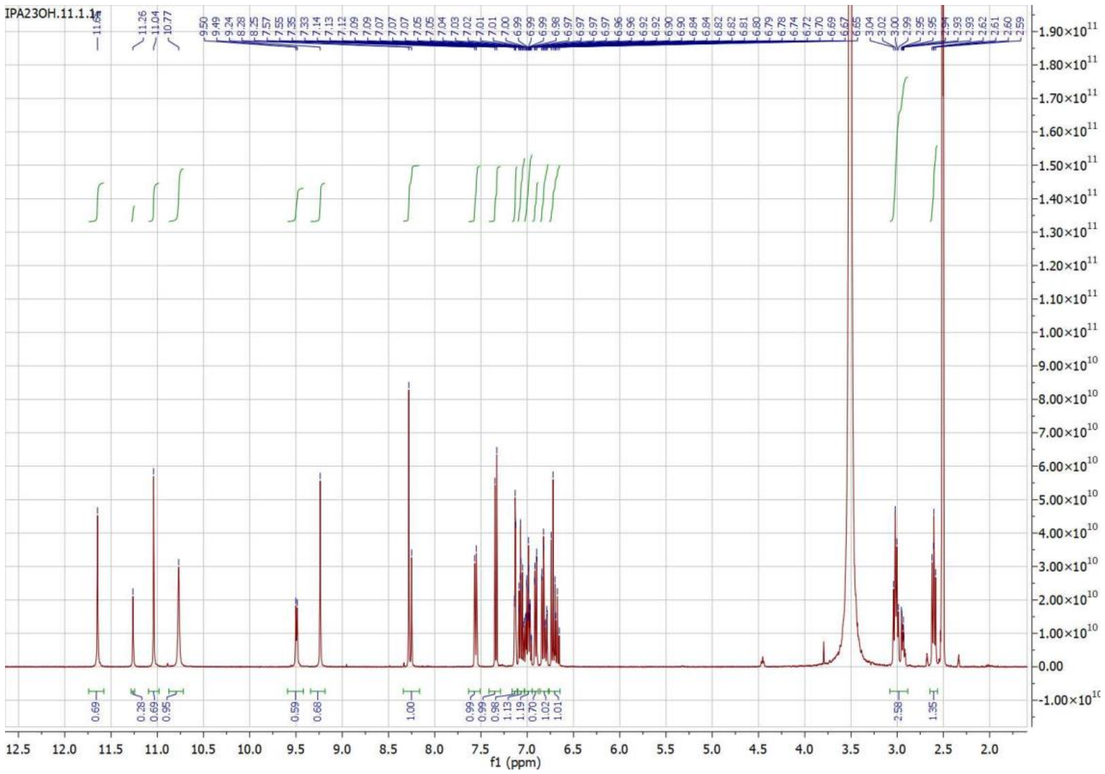

Figure S1. ^{1}H NMRspectrum of **2** (400 MHz, DMSO-d_6

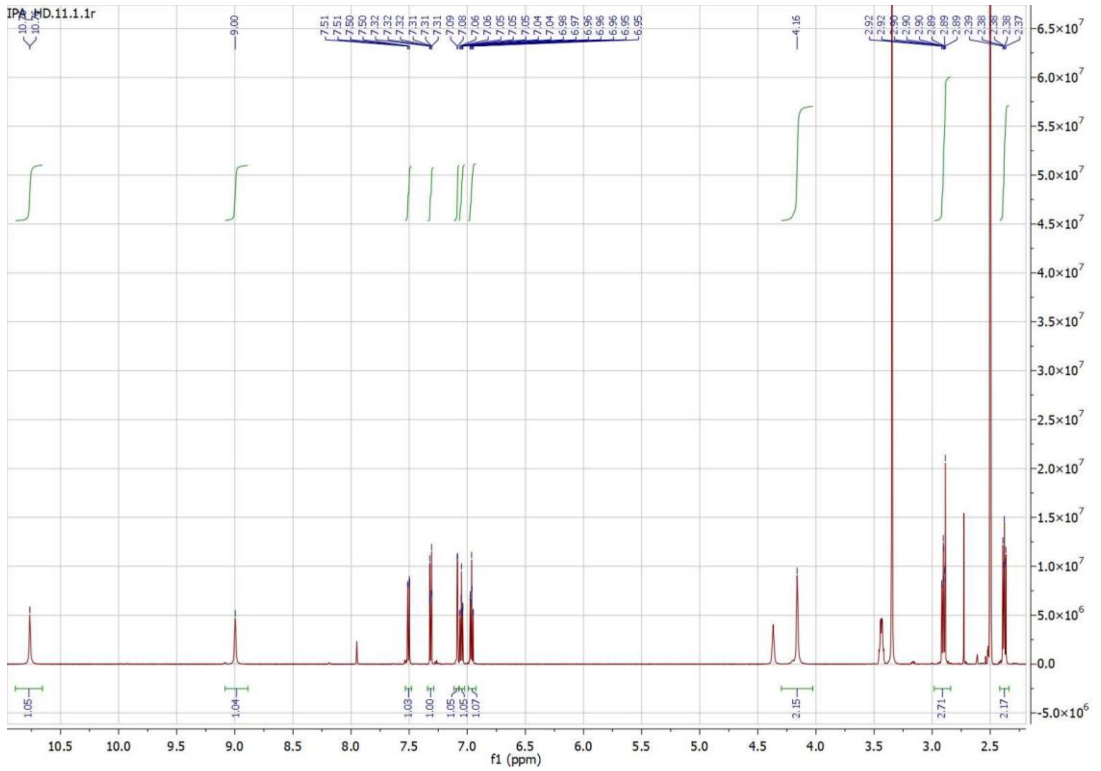

Figure S2. ^{1}H NMRspectrum of **(3a)** (400 MHz, DMSO-d_6

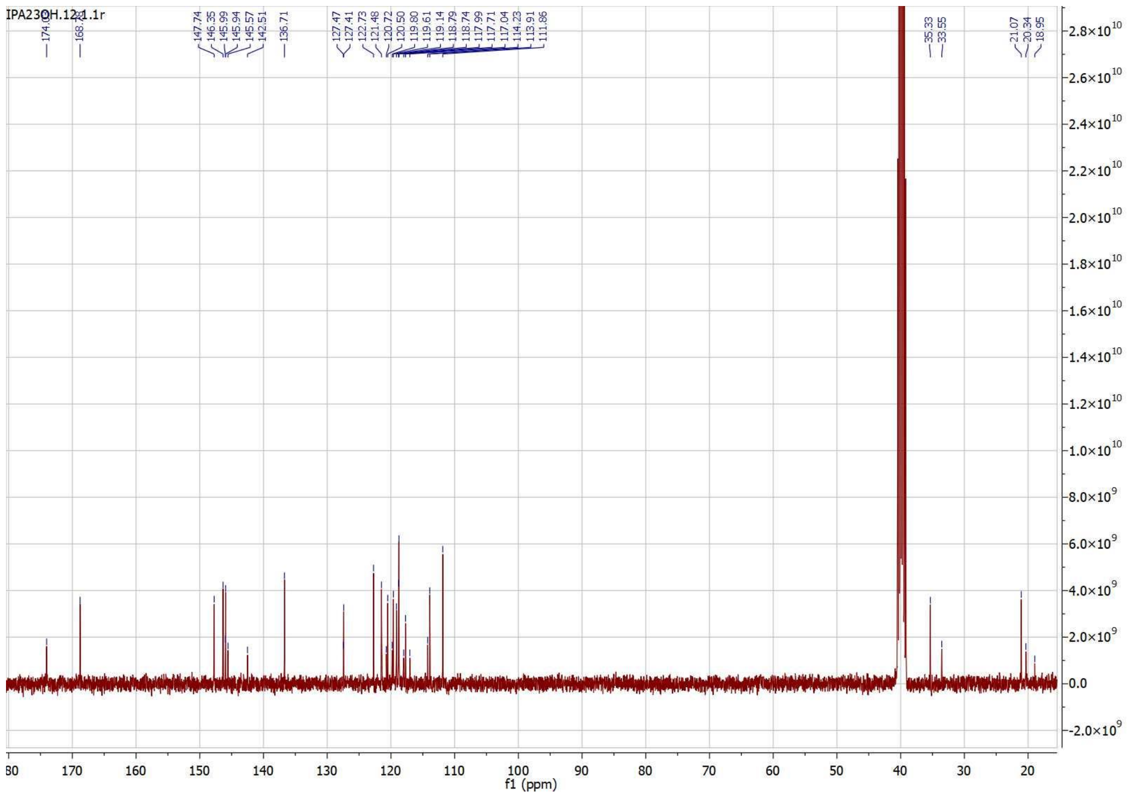

Figure S3. ^{13}C NMR spectrum of **3a** (151 MHz, DMSO-d_6).

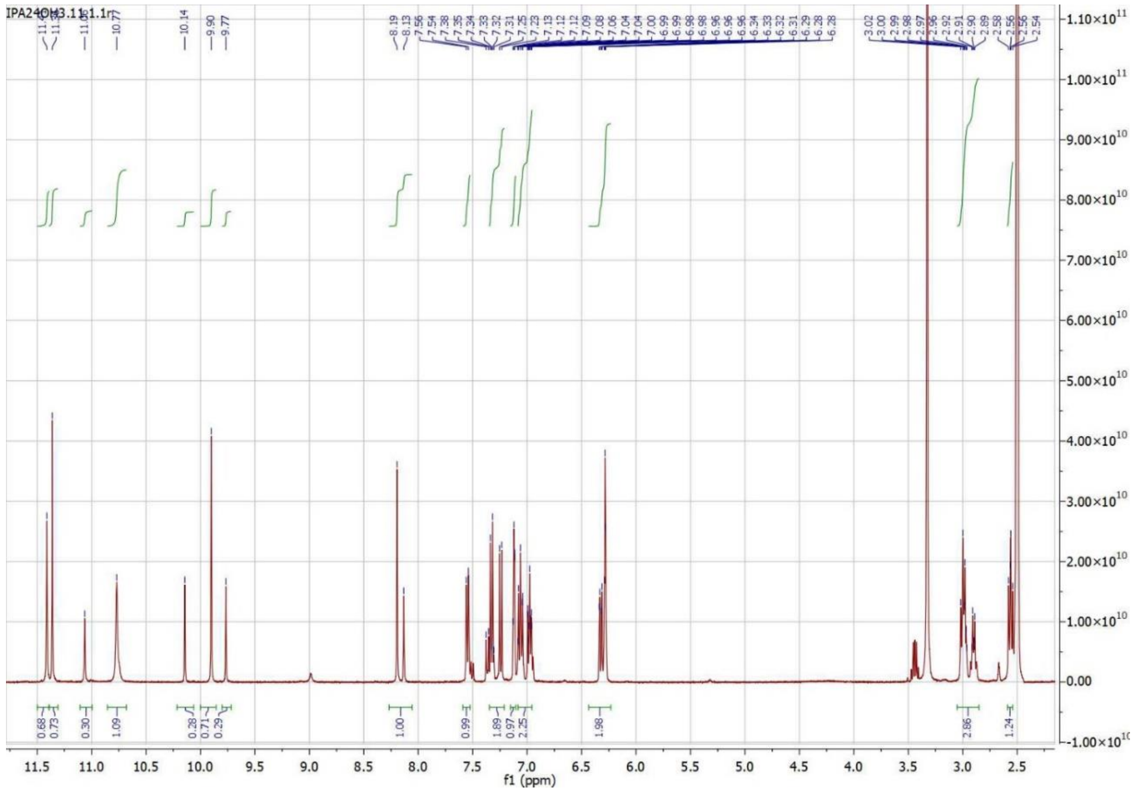

Figure S4. ^{1}H NMR spectrum of **(3c)** (600 MHz, DMSO-d_6).

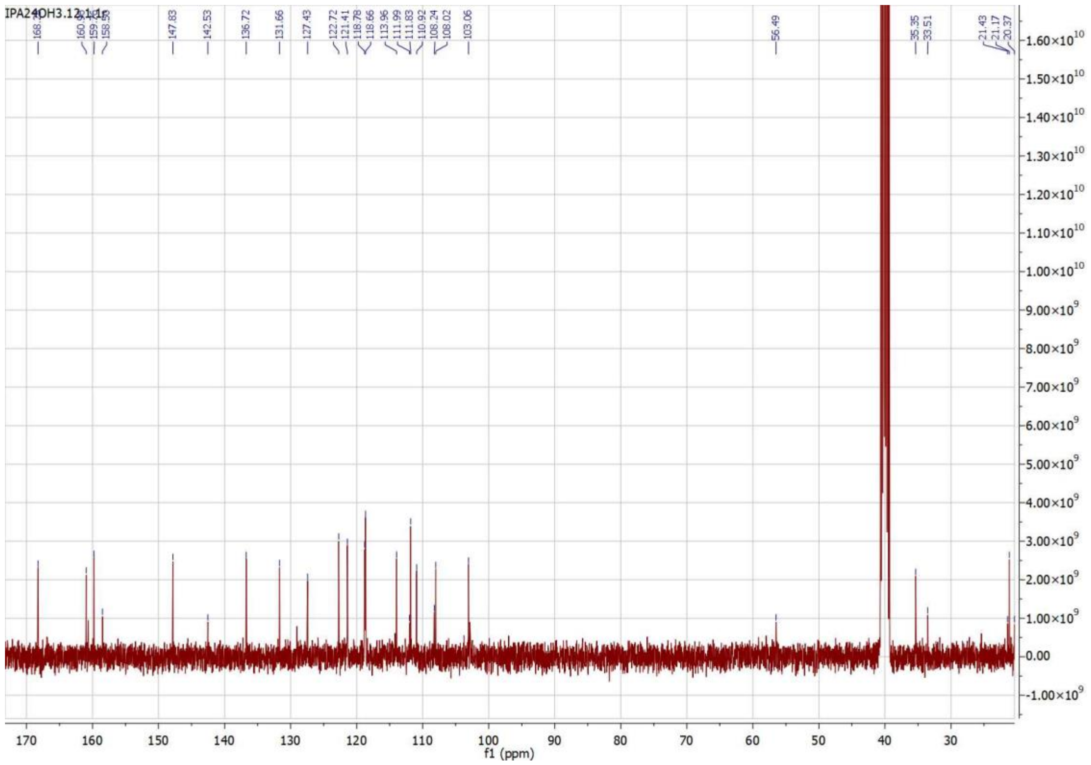

Figure S5. ^{13}C NMR spectrum of **3c** (151 MHz, DMSO-d$_6$).

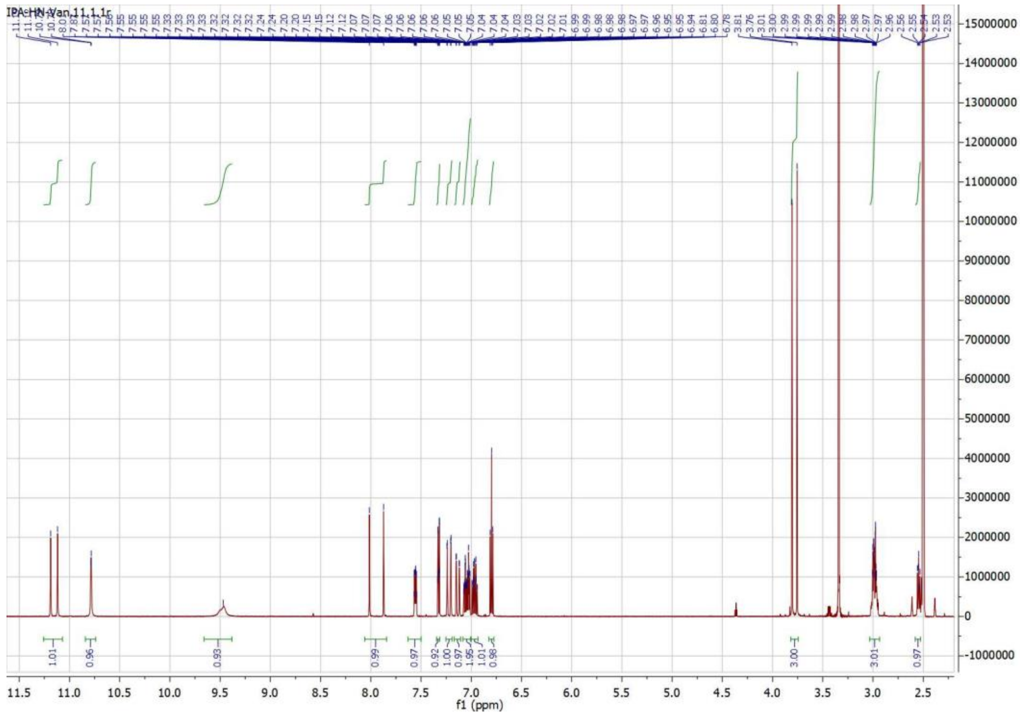

Figure S6. ^{1}H NMRspectrum of **(3d)** (600 MHz, DMSO-d$_6$).

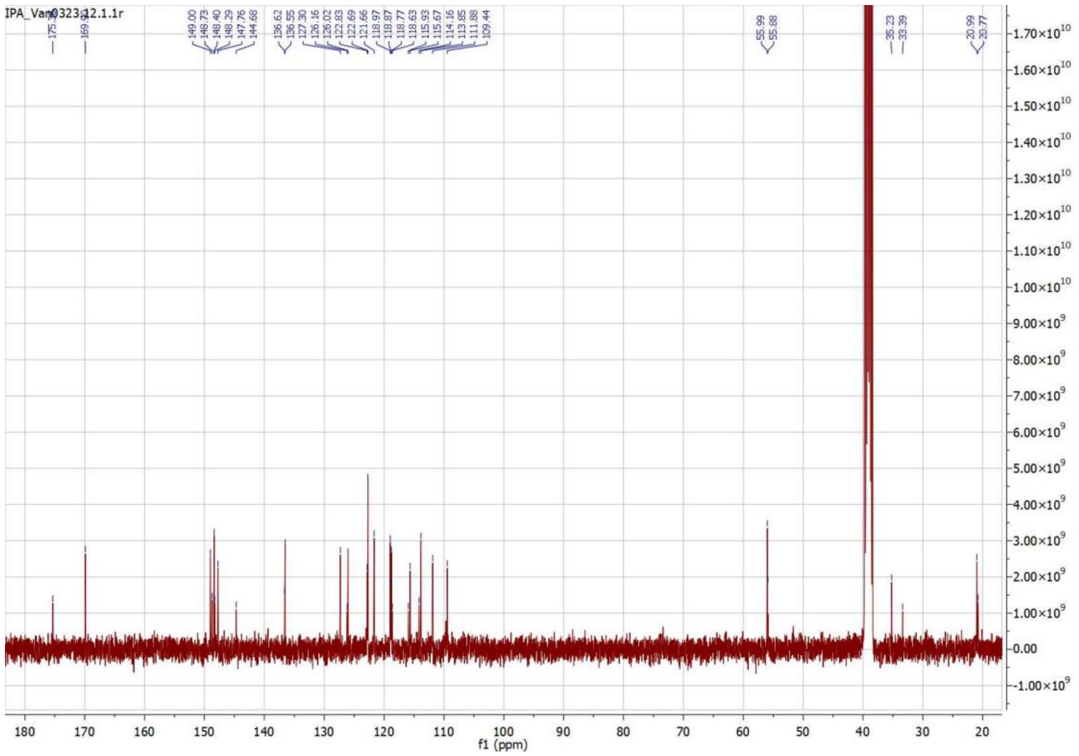

Figure S7. ^{13}C NMR spectrum of **3d** (151 MHz, DMSO-d_6).

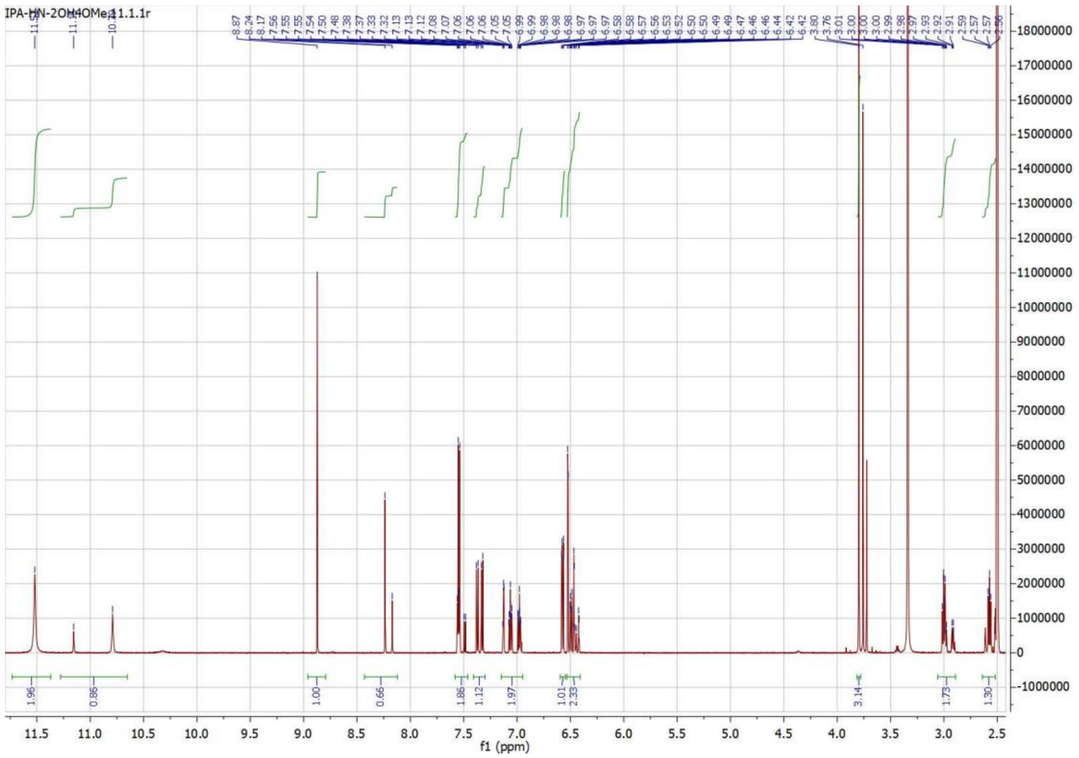

Figure S8. ^{1}H NMRspectrum of **(3e)** (600 MHz, DMSO-d_6).

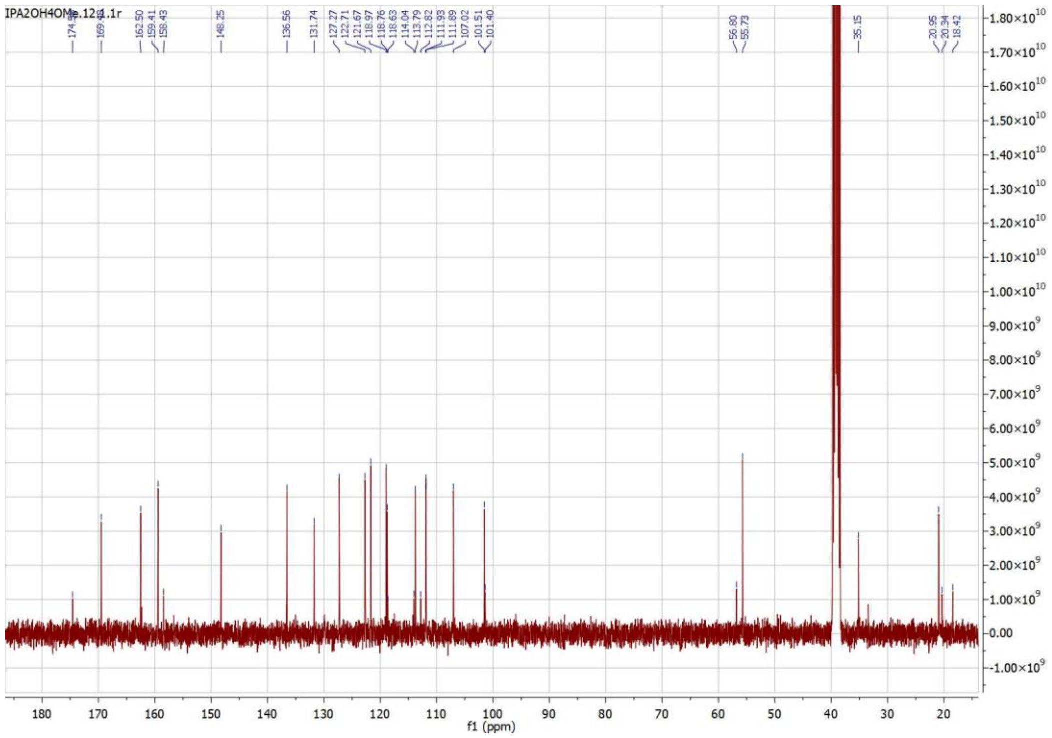

Figure S9. ^{13}C NMR spectrum of **3e** (151 MHz, DMSO-d_6).

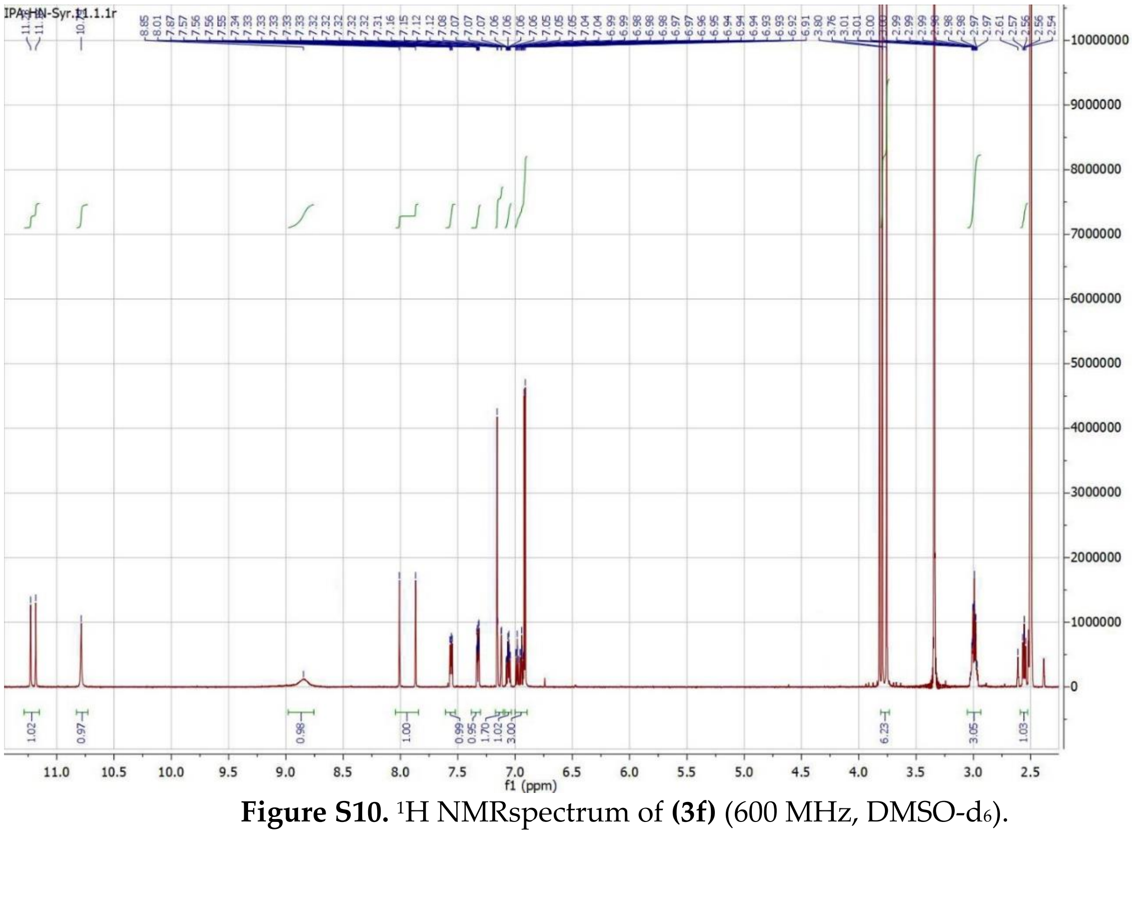

Figure S10. ^1H NMRspectrum of **(3f)** (600 MHz, DMSO-d_6).

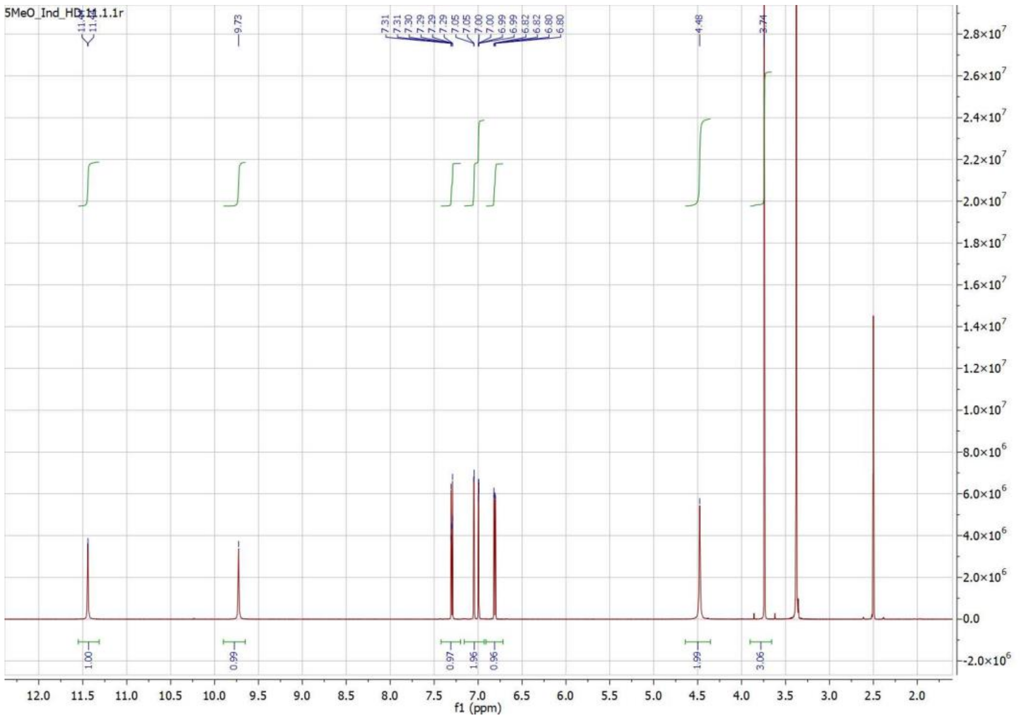

Figure S11. ^{1}H NMRspectrum of **4** (600 MHz, DMSO-d6)

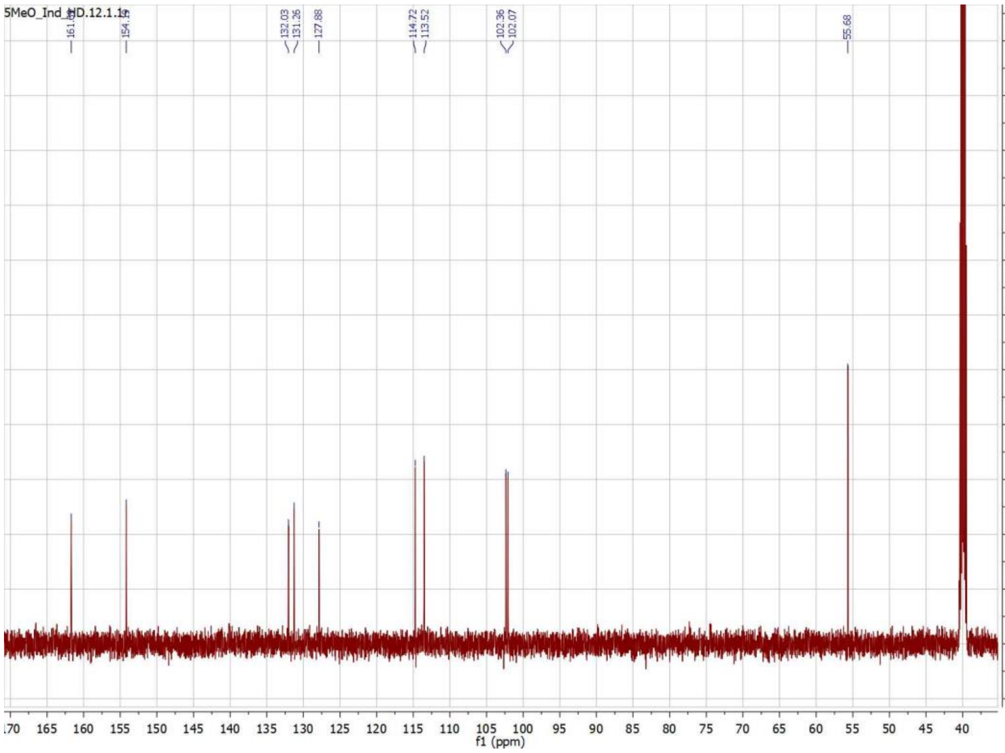

Figure S12. ^{13}C NMR spectrum of **4** (151 MHz, DMSO-d6).

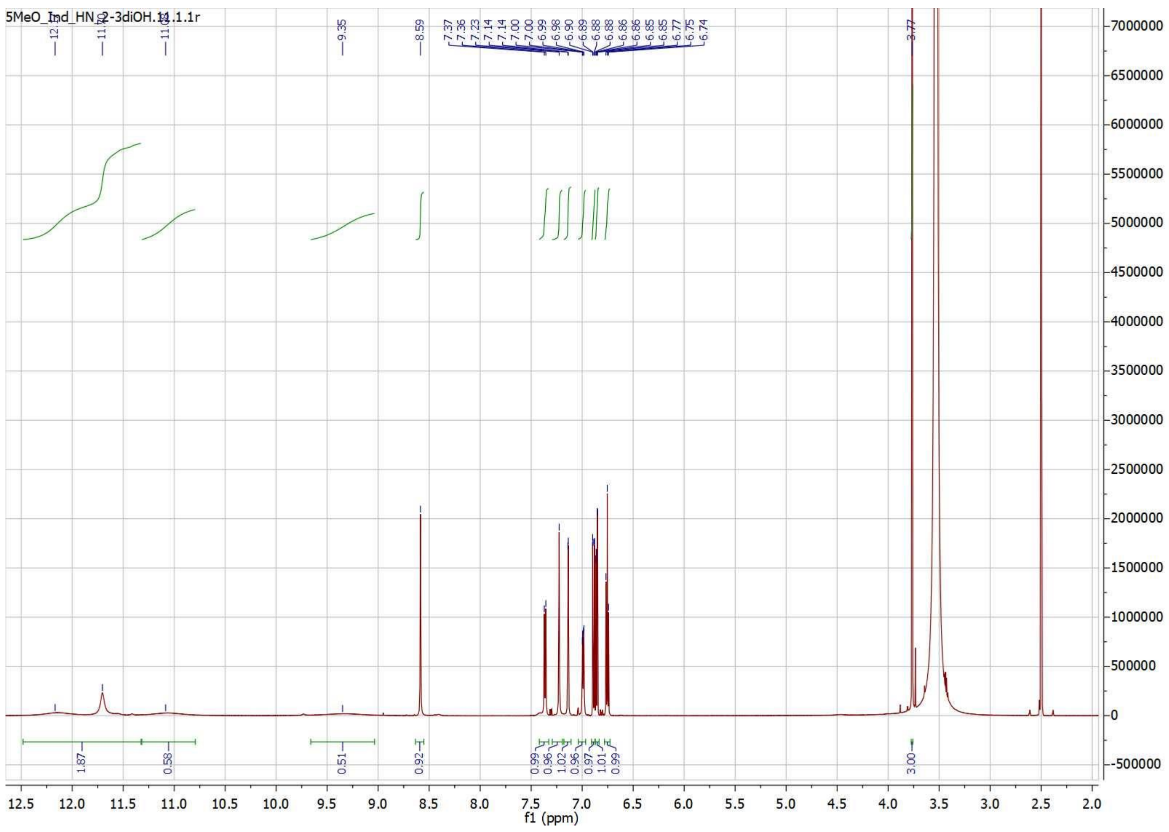

Figure S13. ^{1}H NMRspectrum of **(5a)** (600 MHz, DMSO-d$_6$)

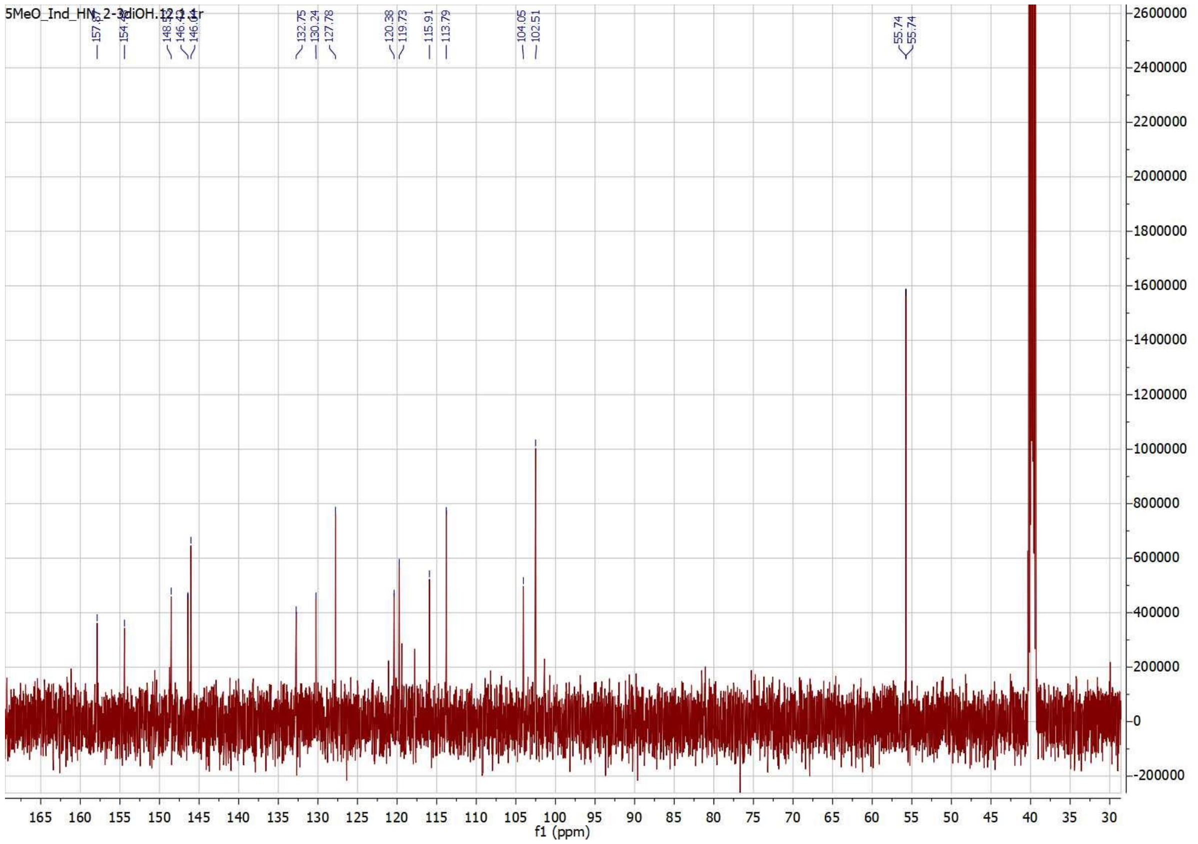

Figure S14. ^{13}C NMR spectrum of **5a** (151 MHz, DMSO-d$_6$).

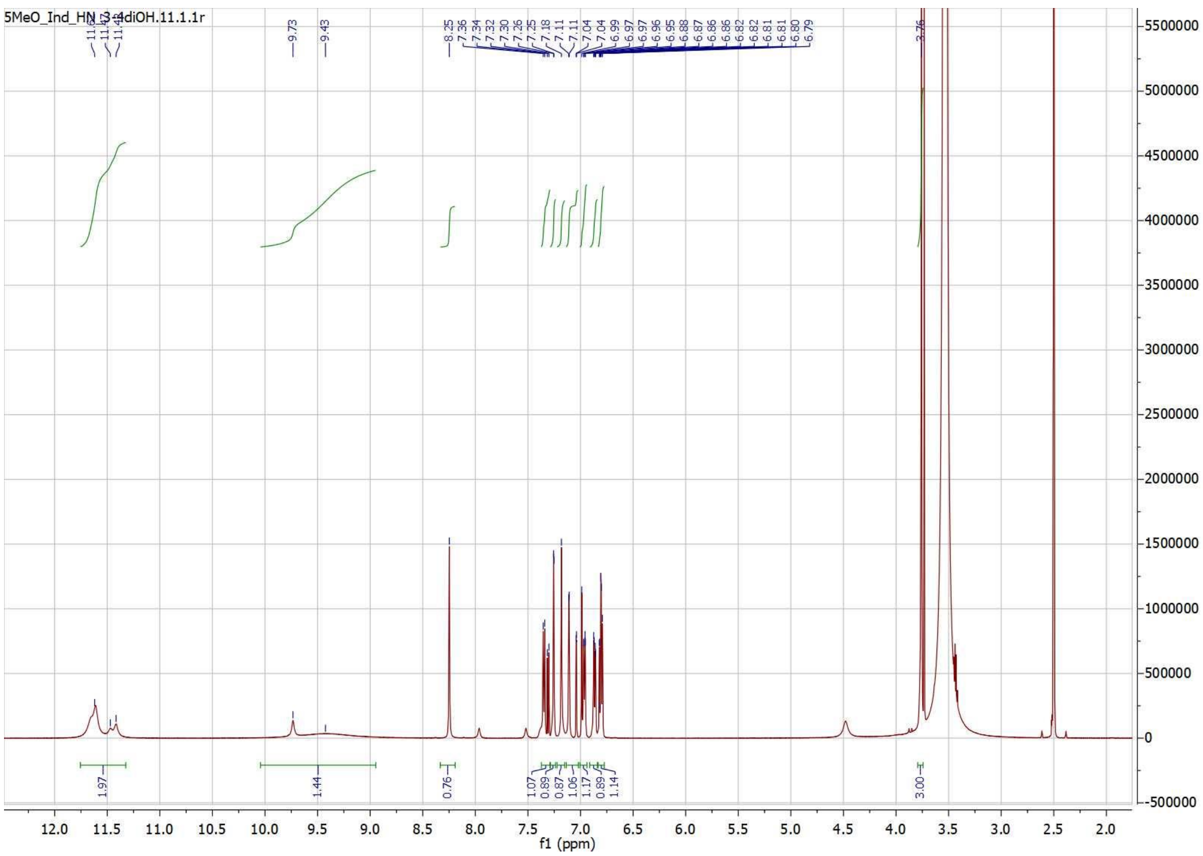

Figure S15. ^{1}H NMR spectrum of **5b** (600 MHz, DMSO-d_6).

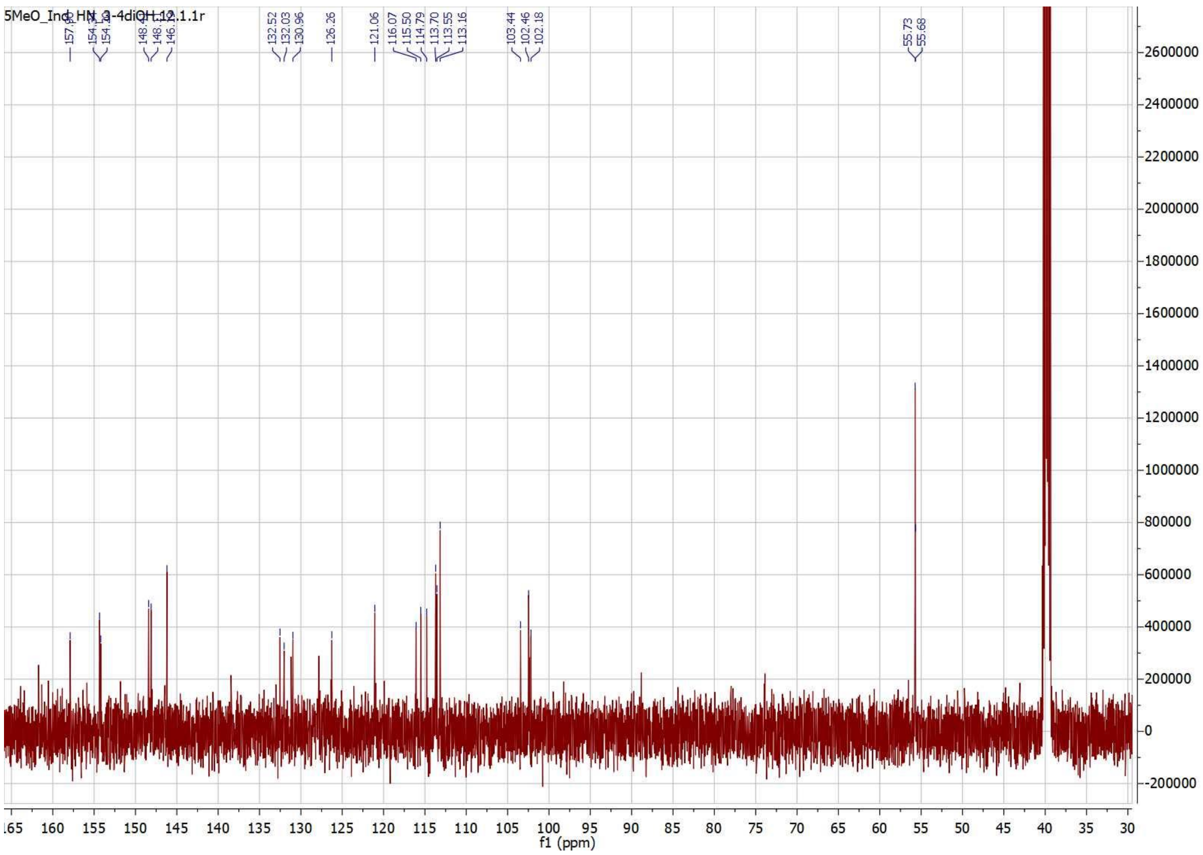

Figure S16. ^{13}C NMR spectrum of **5b** (151 MHz, DMSO-d_6).

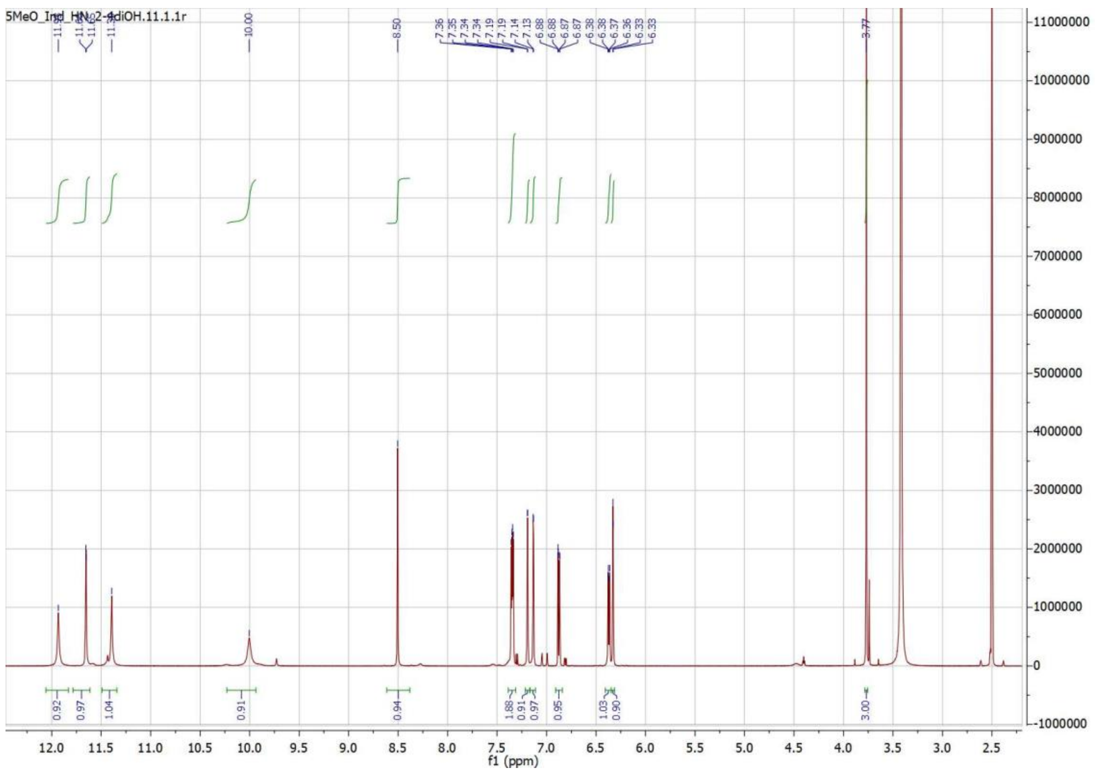

Figure S17. ^{1}H NMR spectrum of **5c** (600 MHz, DMSO-d_6)

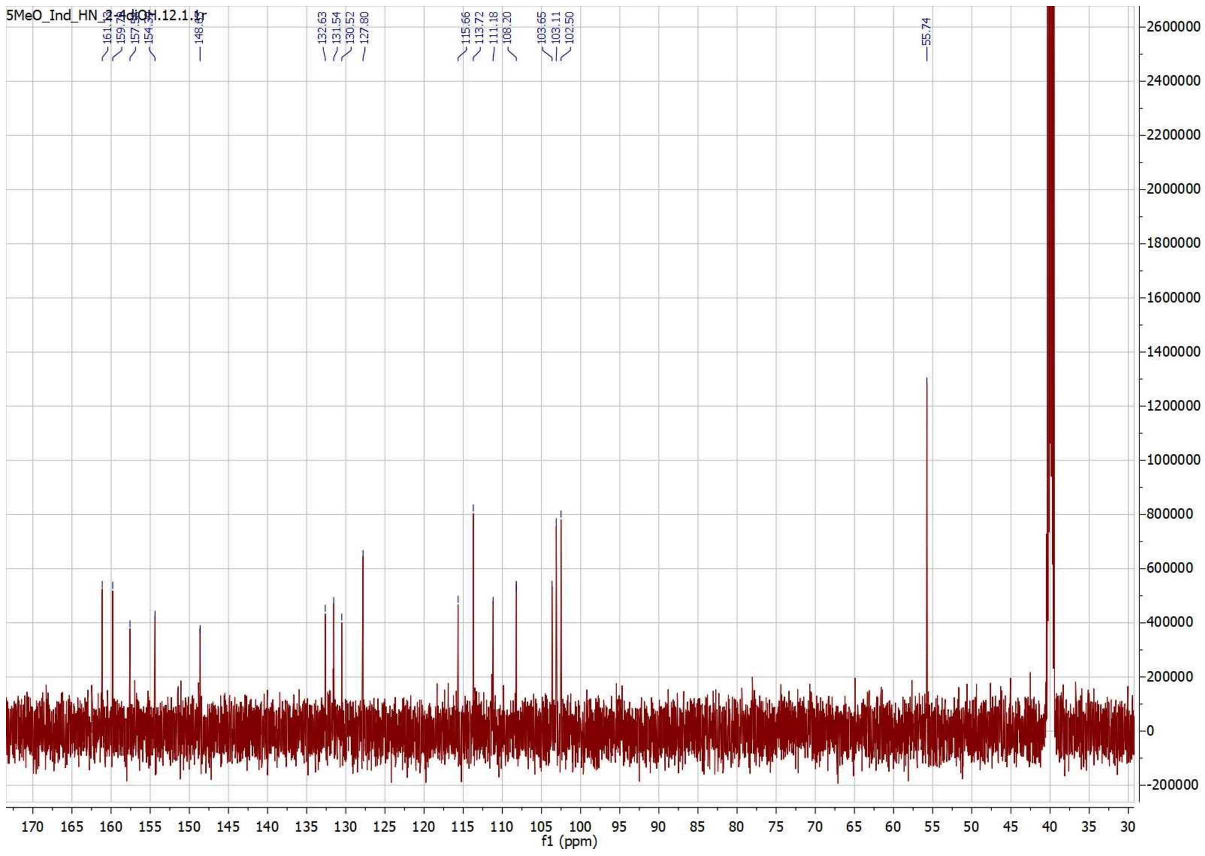

Figure S18. ^{13}C NMR spectrum of **5c** (151 MHz, DMSO-d_6).

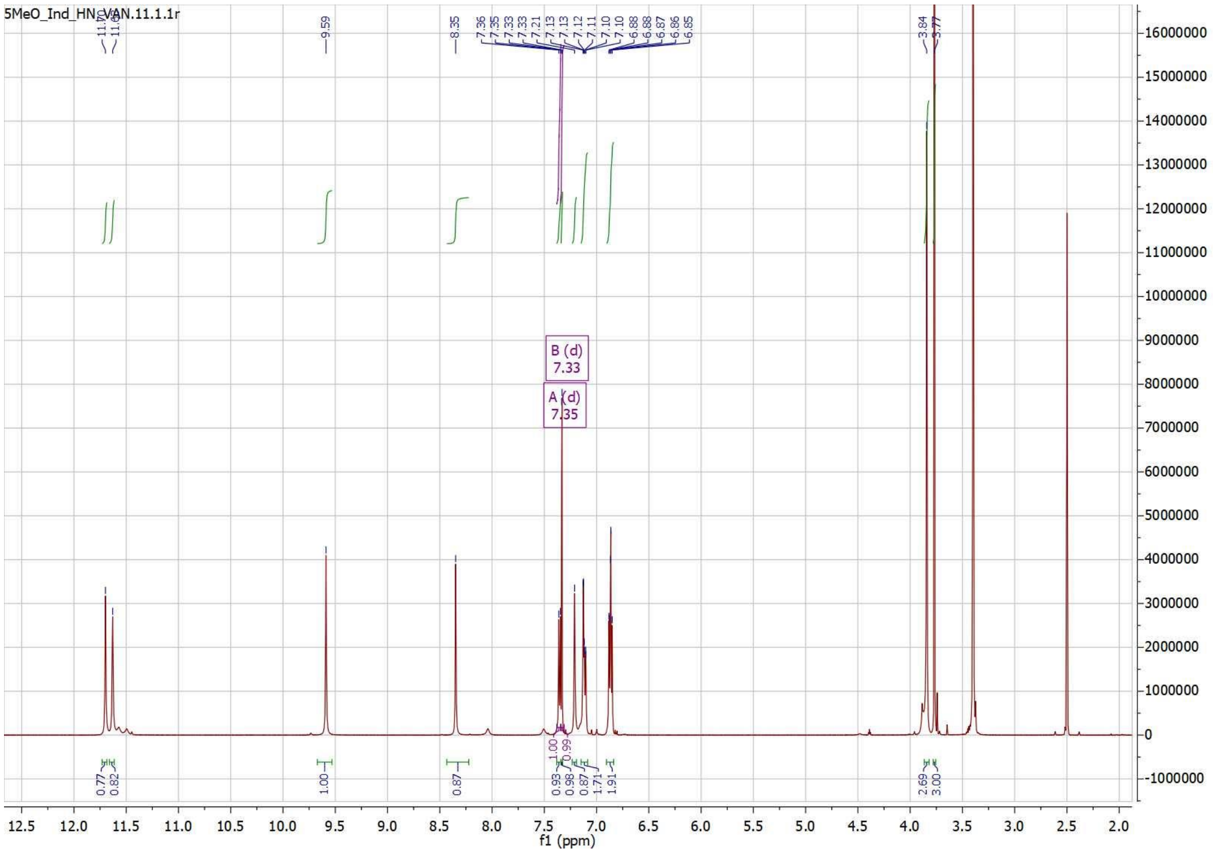

Figure S19. ^{1}H NMR spectrum of 5**d** (600 MHz, DMSO-d_6)

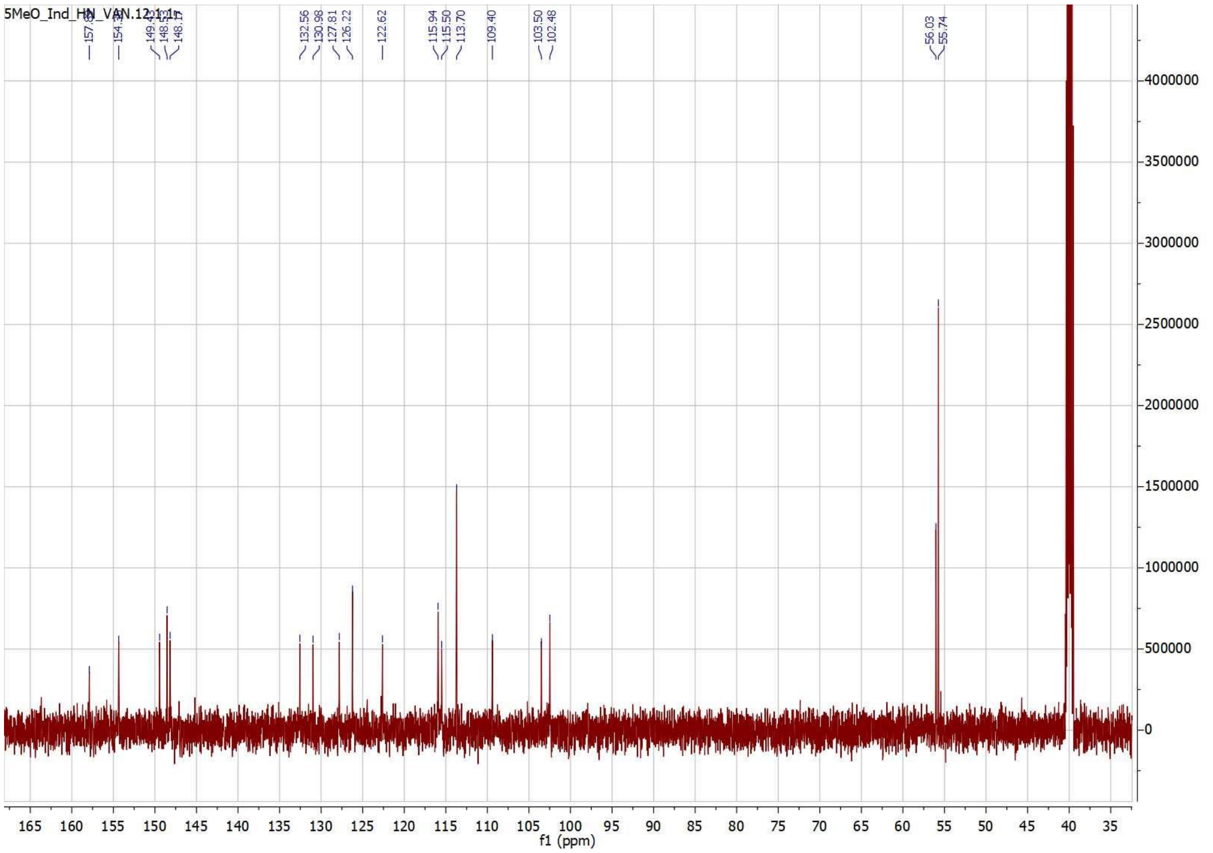

Figure S20. ^{13}C NMR spectrum of **5d** (151 MHz, DMSO-d_6)

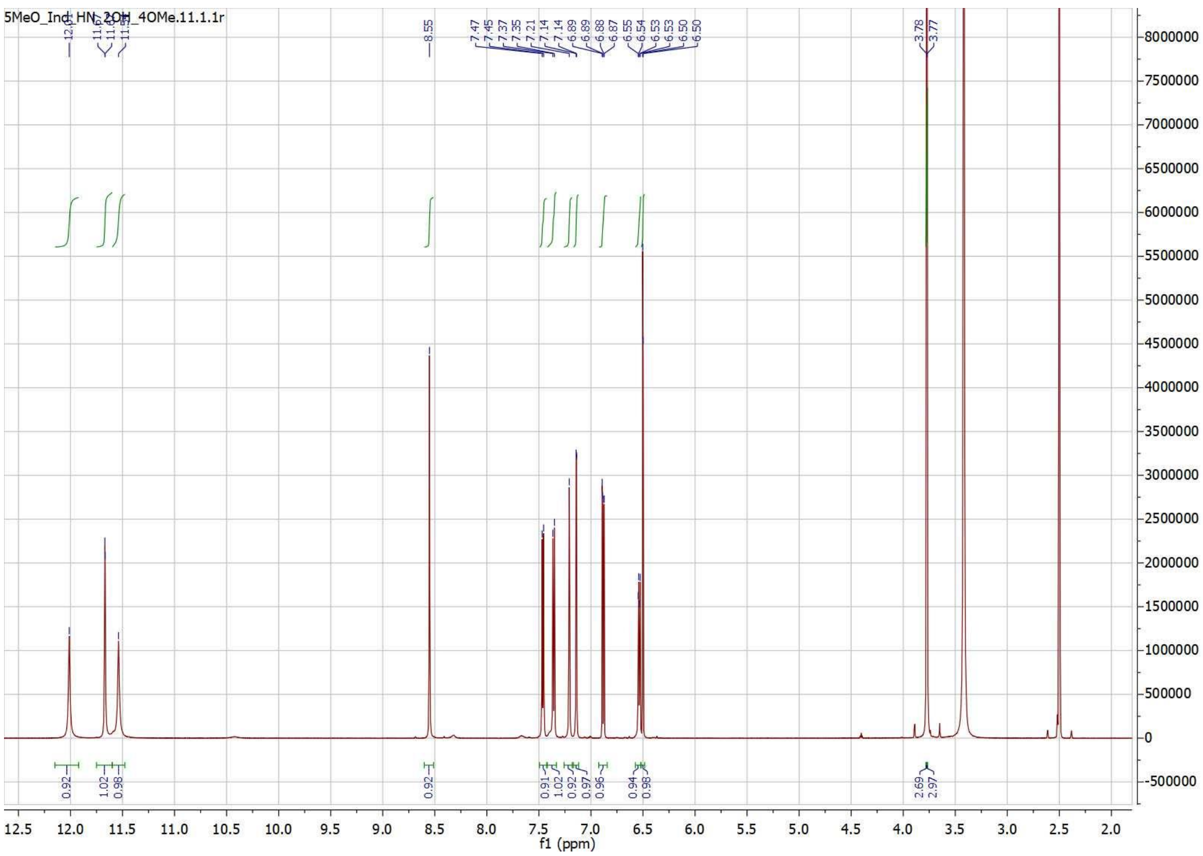

Figure S21. ^{1}H NMR spectrum of **5e** (600 MHz, DMSO-d_6)

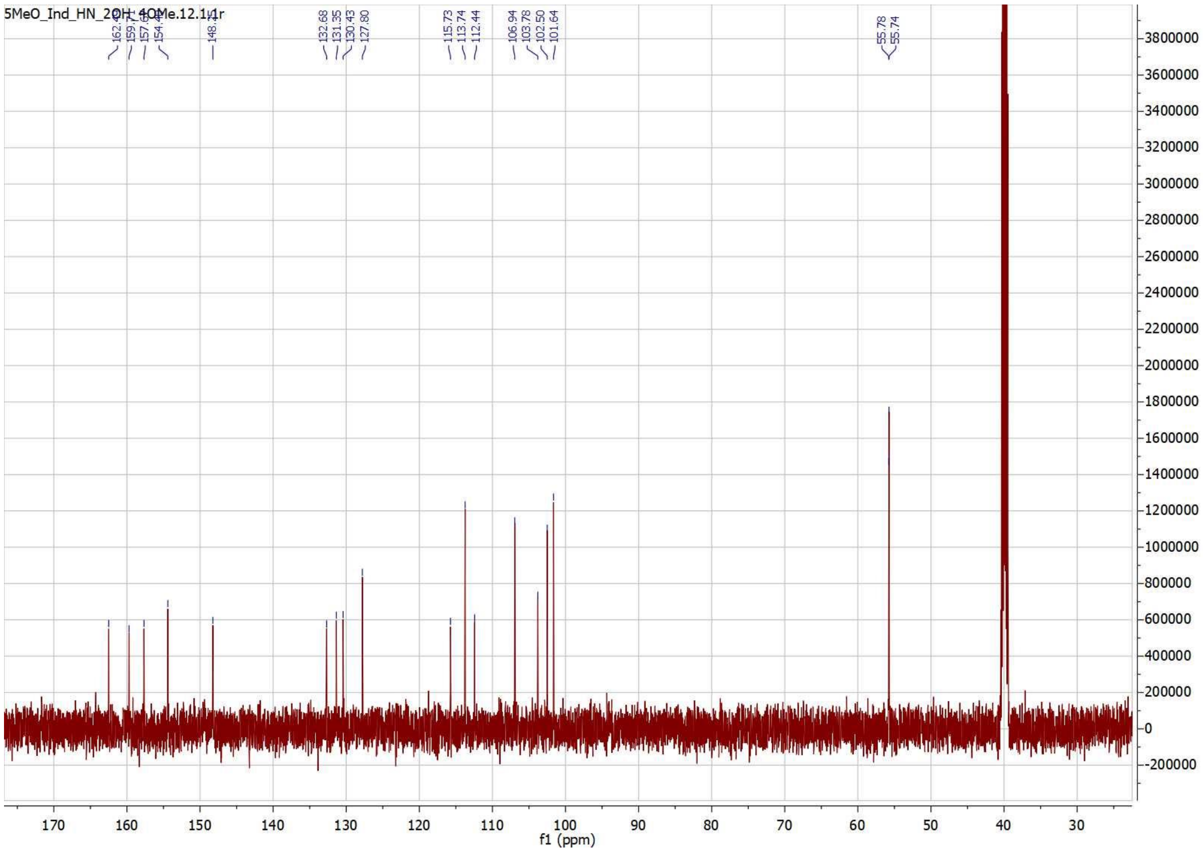

Figure S22. ^{13}C NMR spectrum of **5e** (151 MHz, DMSO-d_6)

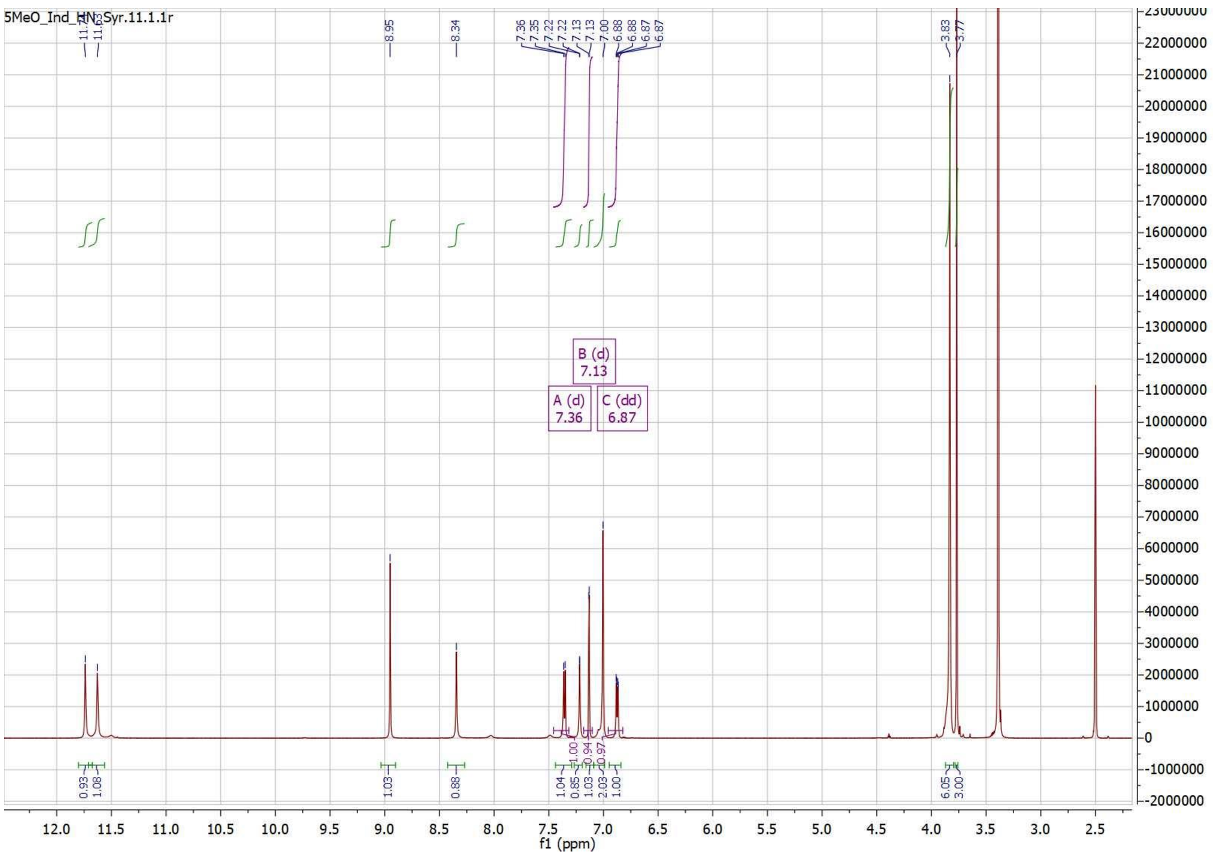

Figure S23. ^{1}H NMR spectrum of **5f** (600 MHz, DMSO-d_6)

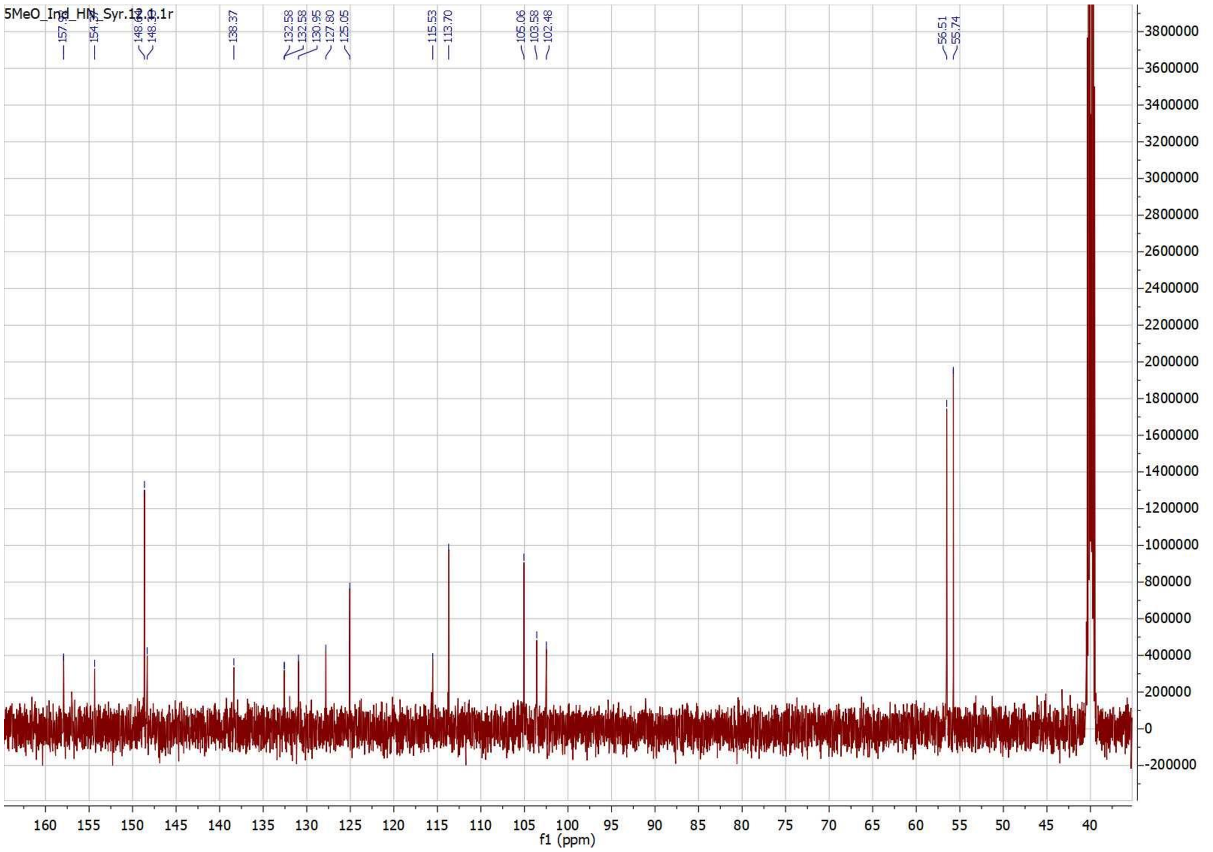

Figure S24. ^{13}C NMR spectrum of **5f** (151 MHz, DMSO-d_6)

3. IR spectra of the synthesized compounds

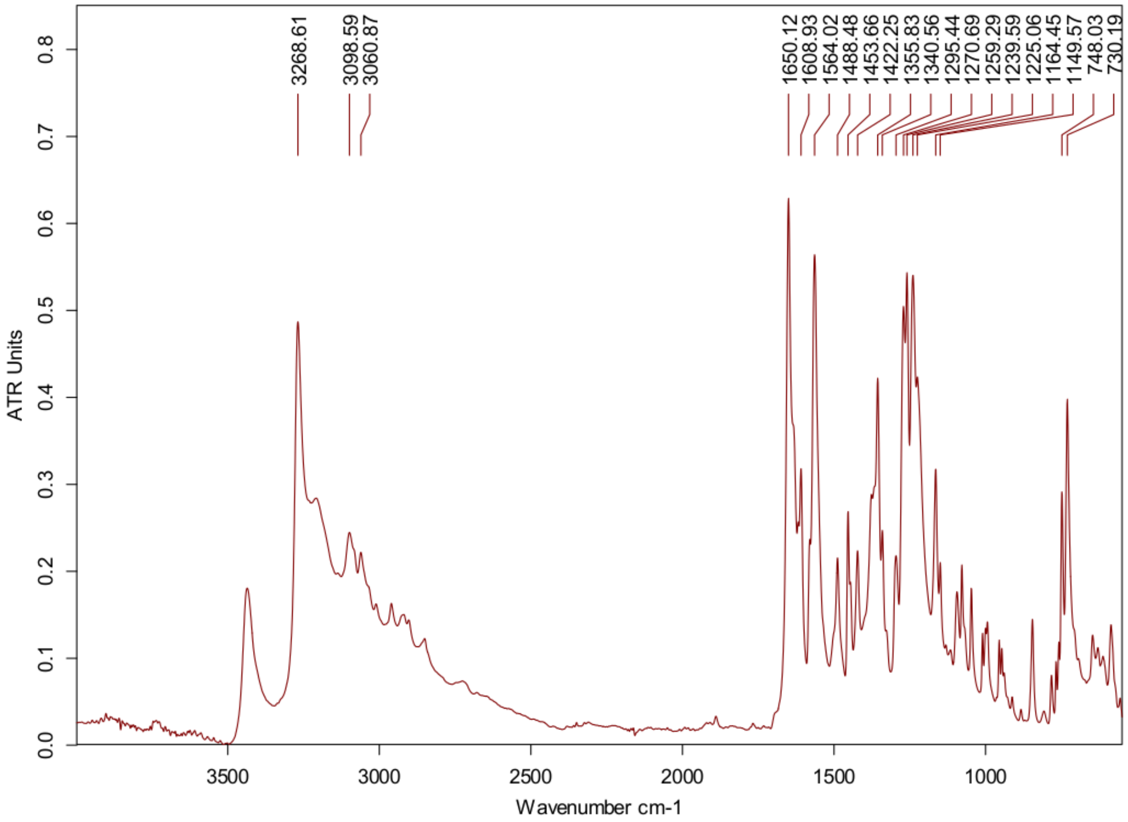

Figure S25. ATR-IR spectrum of **3a**

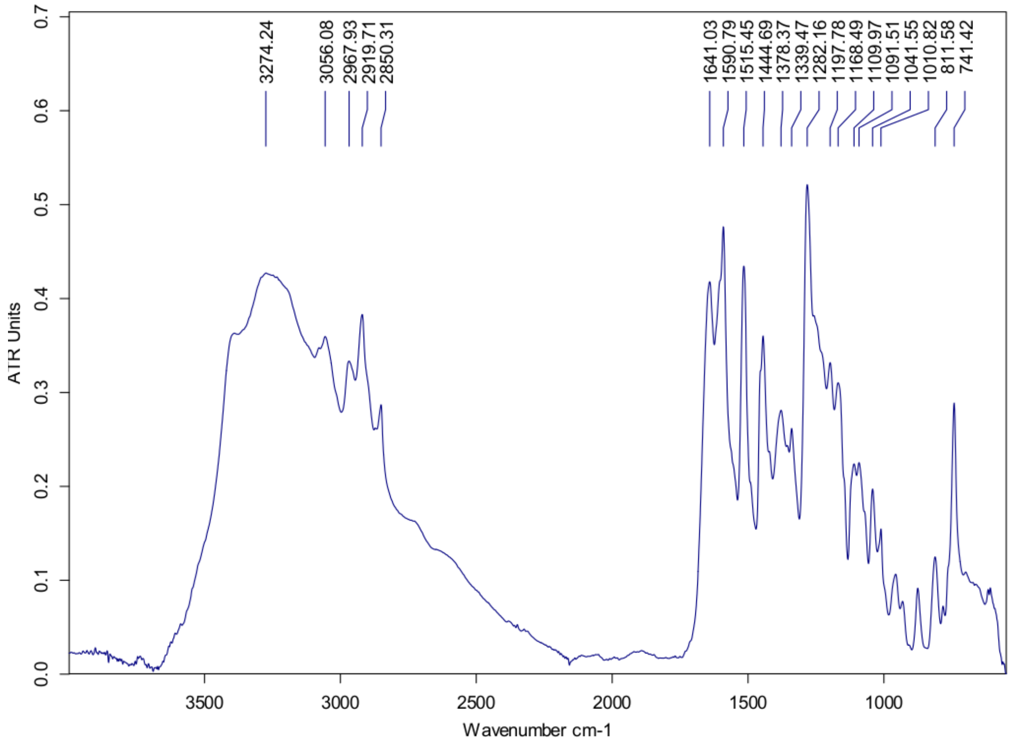

Figure S26. ATR-IR spectrum of **3b**

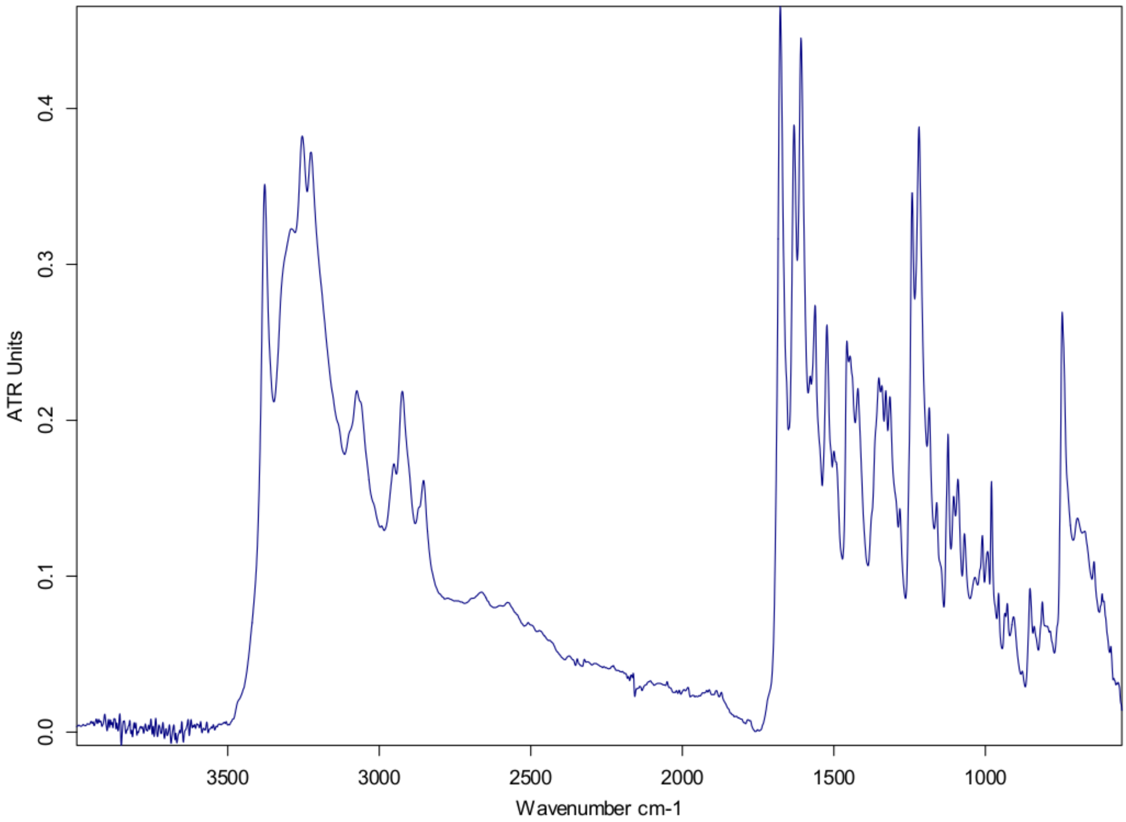

Figure S27. ATR-IR spectrum of **3c**

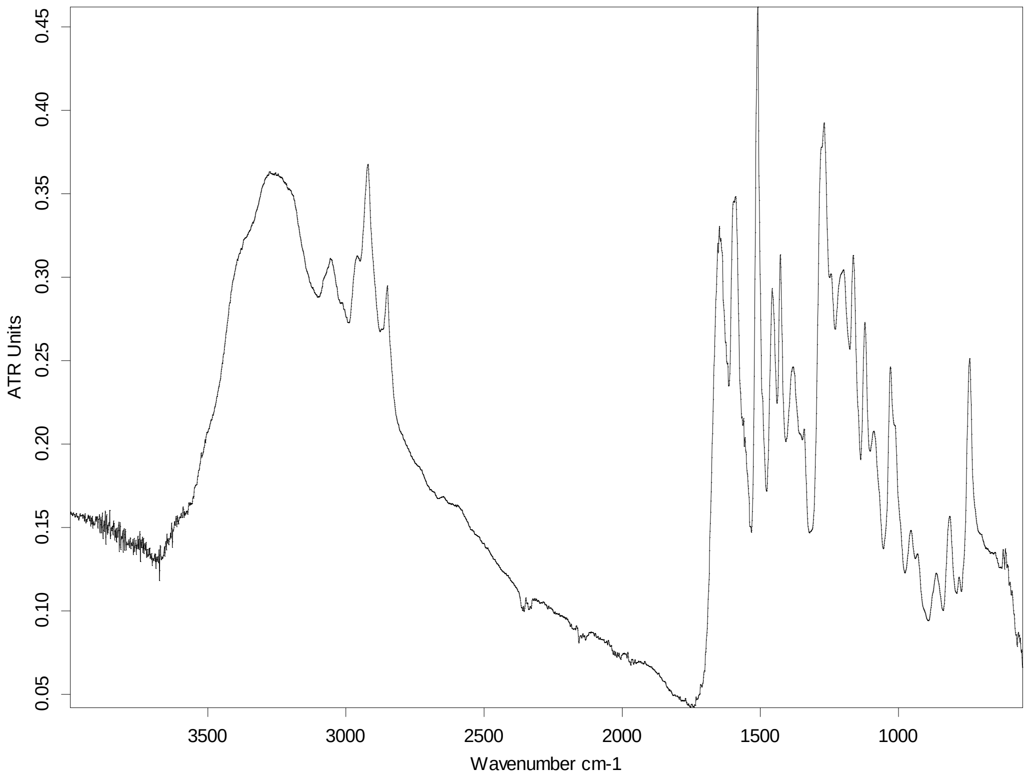

Figure S28. ATR-IR spectrum of **3d**

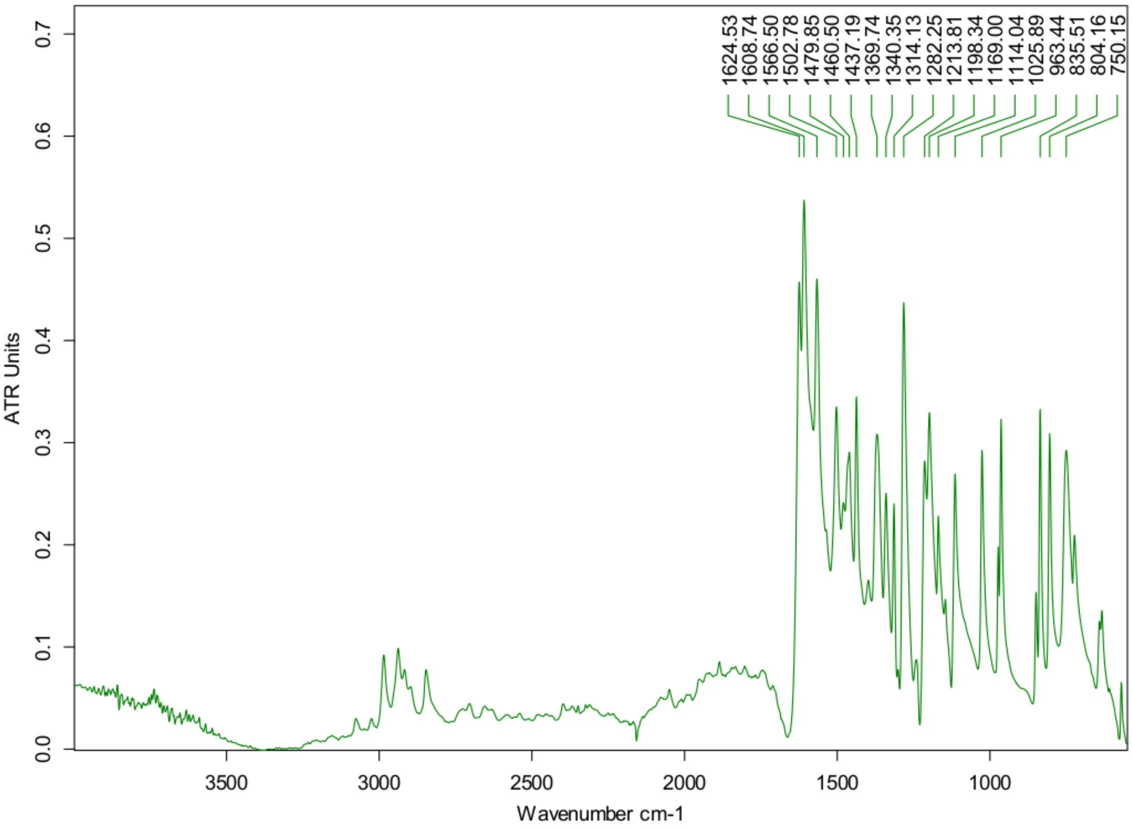

Figure S29. ATR-IR spectrum of **3e**

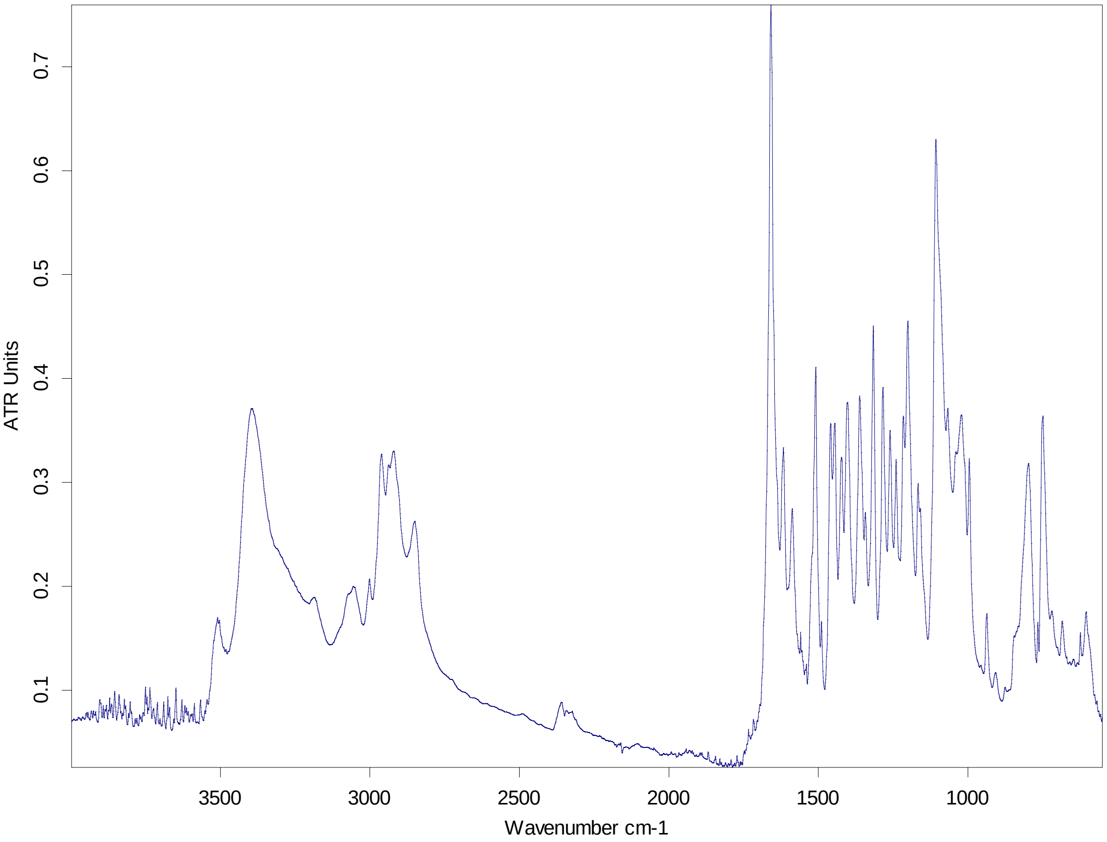

Figure S30. ATR-IR spectrum of **3f**

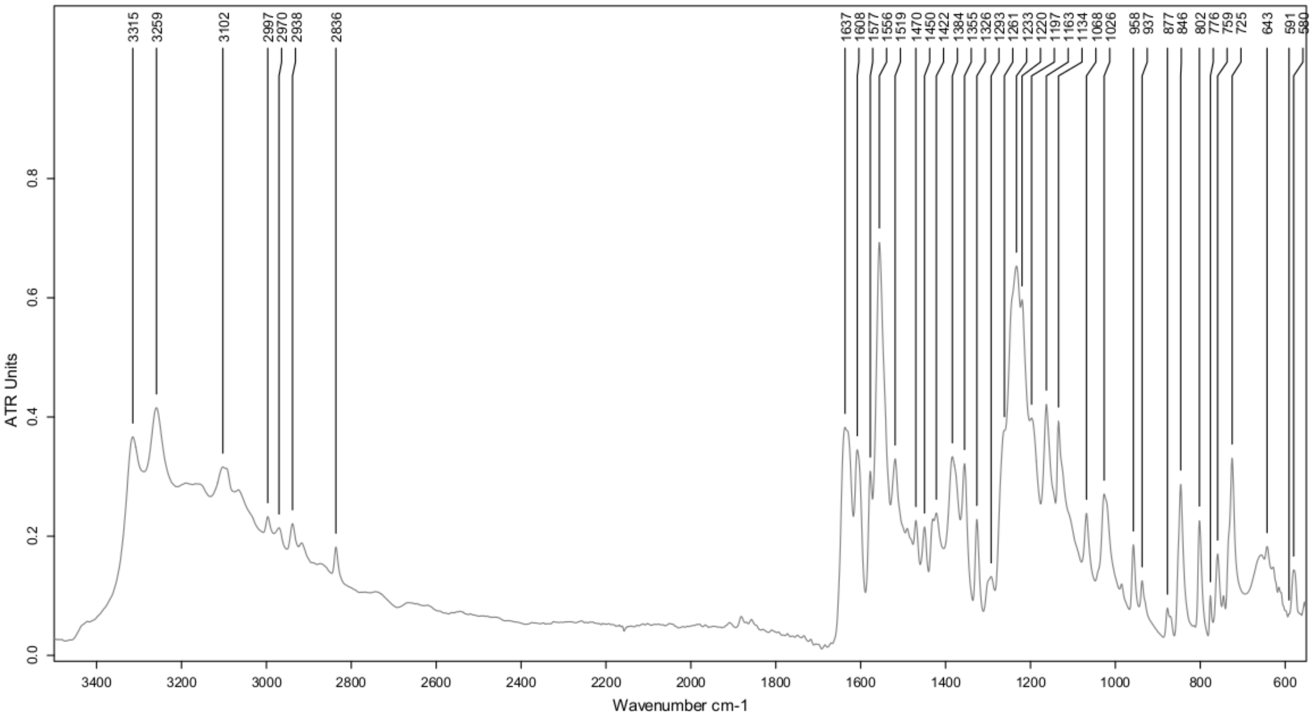

Figure S31. ATR-IR spectrum of **5a**

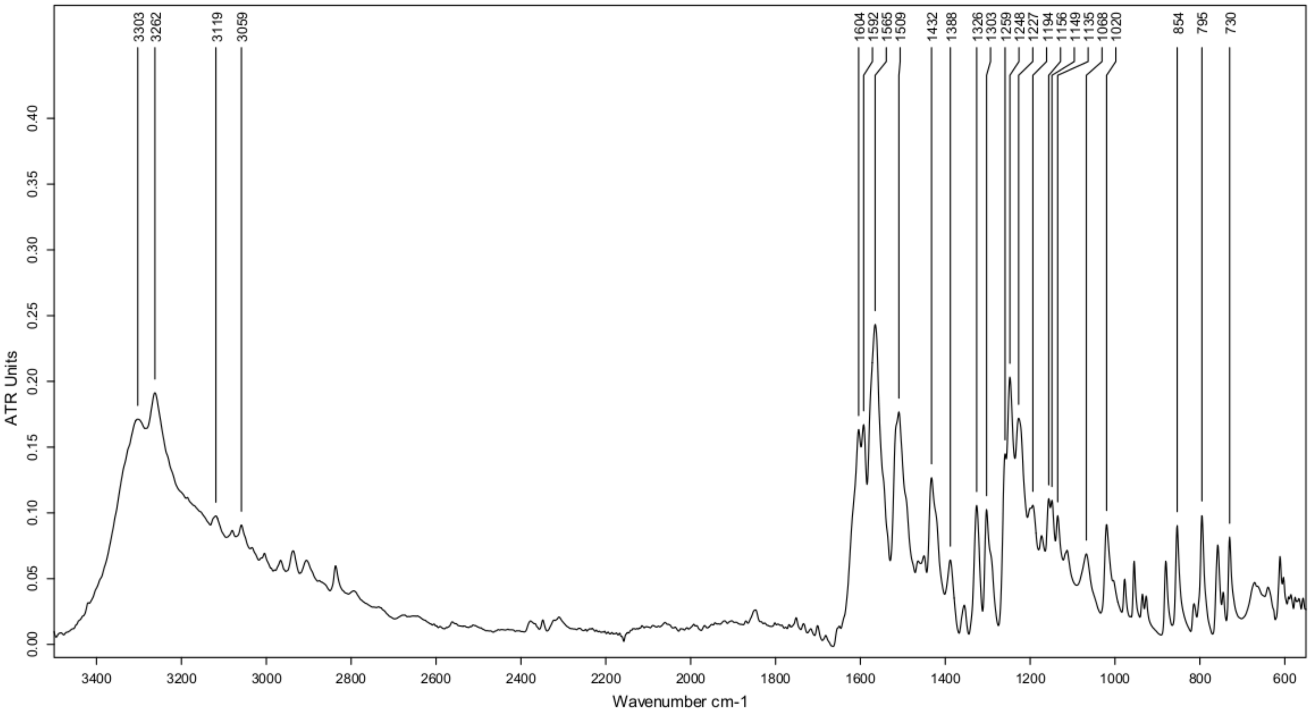

Figure S32. ATR-IR spectrum of **5b**

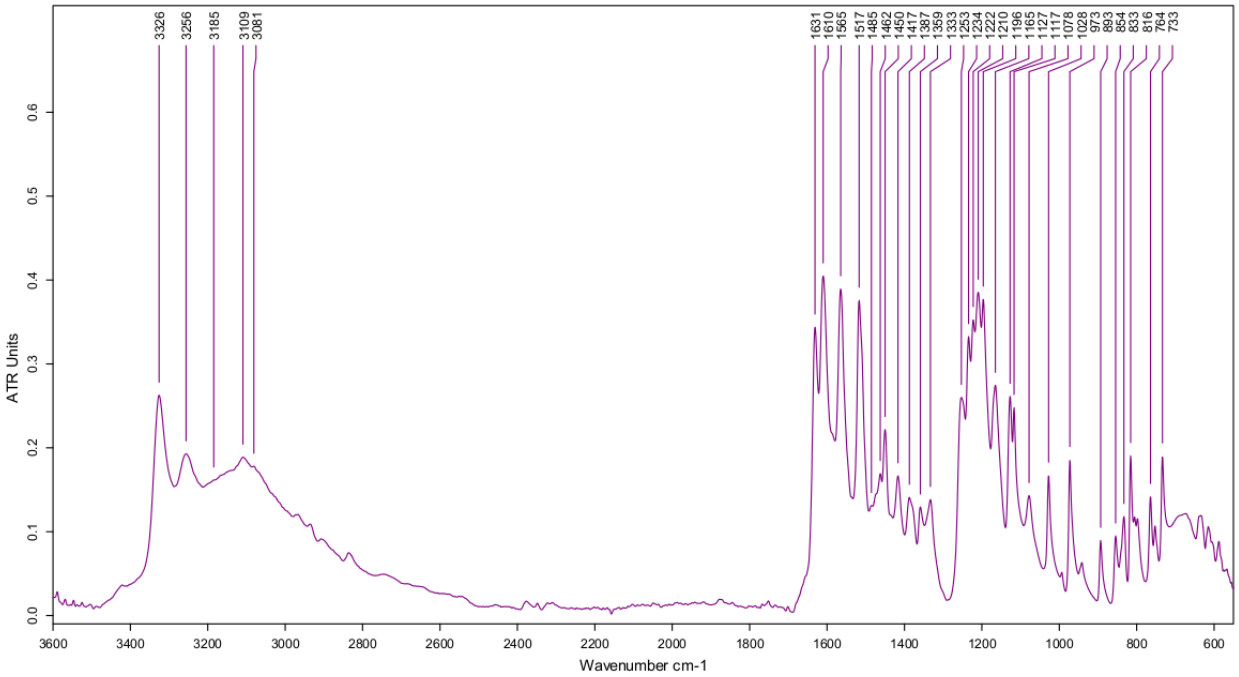

Figure S33. ATR-IR spectrum of **5c**

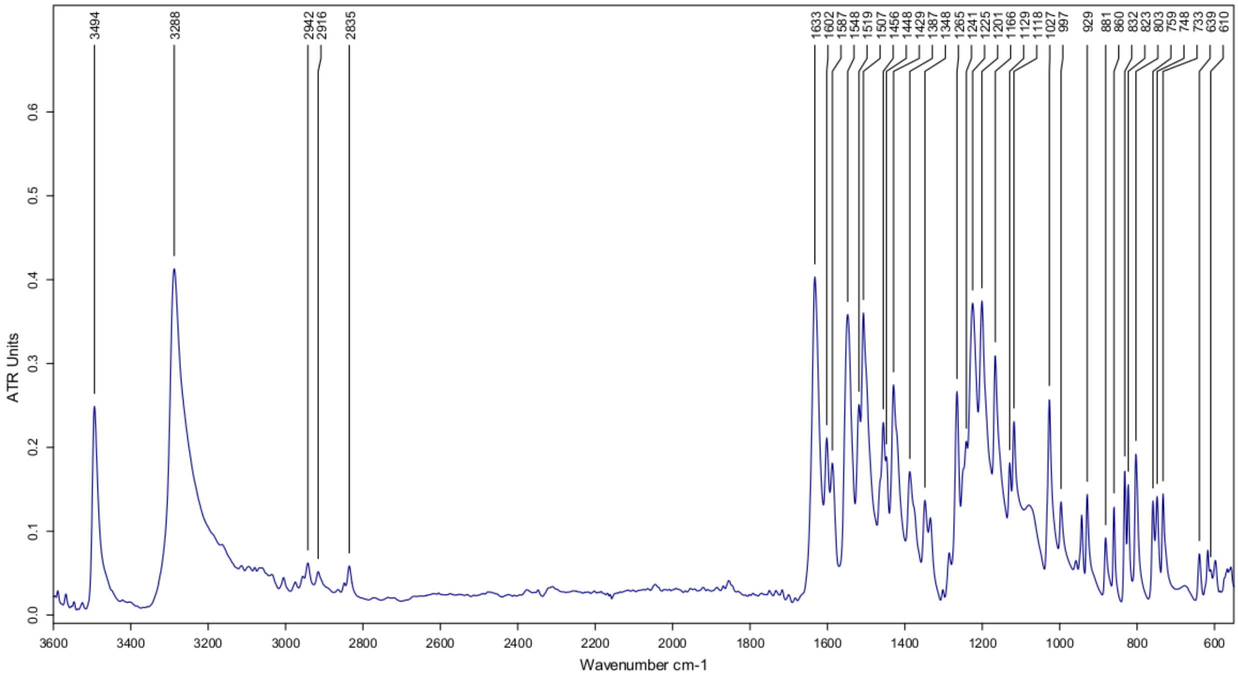

Figure S34. ATR-IR spectrum of **5d**

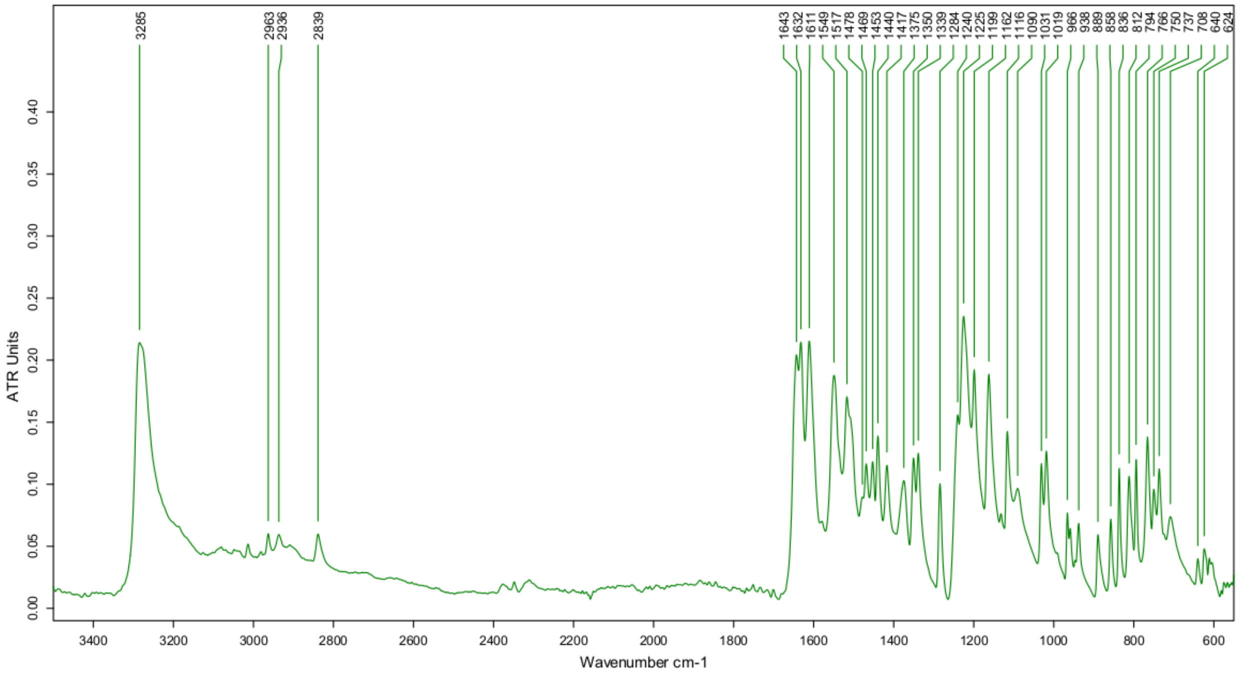

Figure S35. ATR-IR spectrum of **5e**

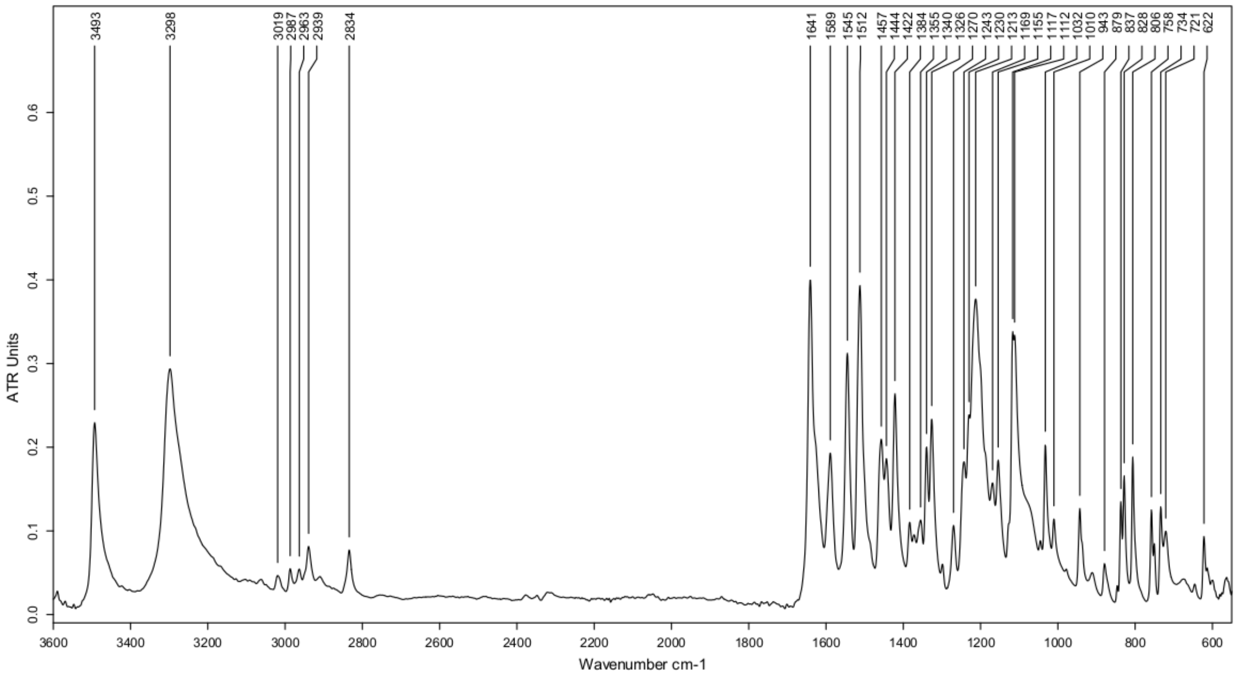

Figure S36. ATR-IR spectrum of **5f**

3. UV-Vis spectra absorbance spectra of the: Control - standard orange-red Fe(II) – 1,10 Phenantroline complex; the tested hydrazone compounds

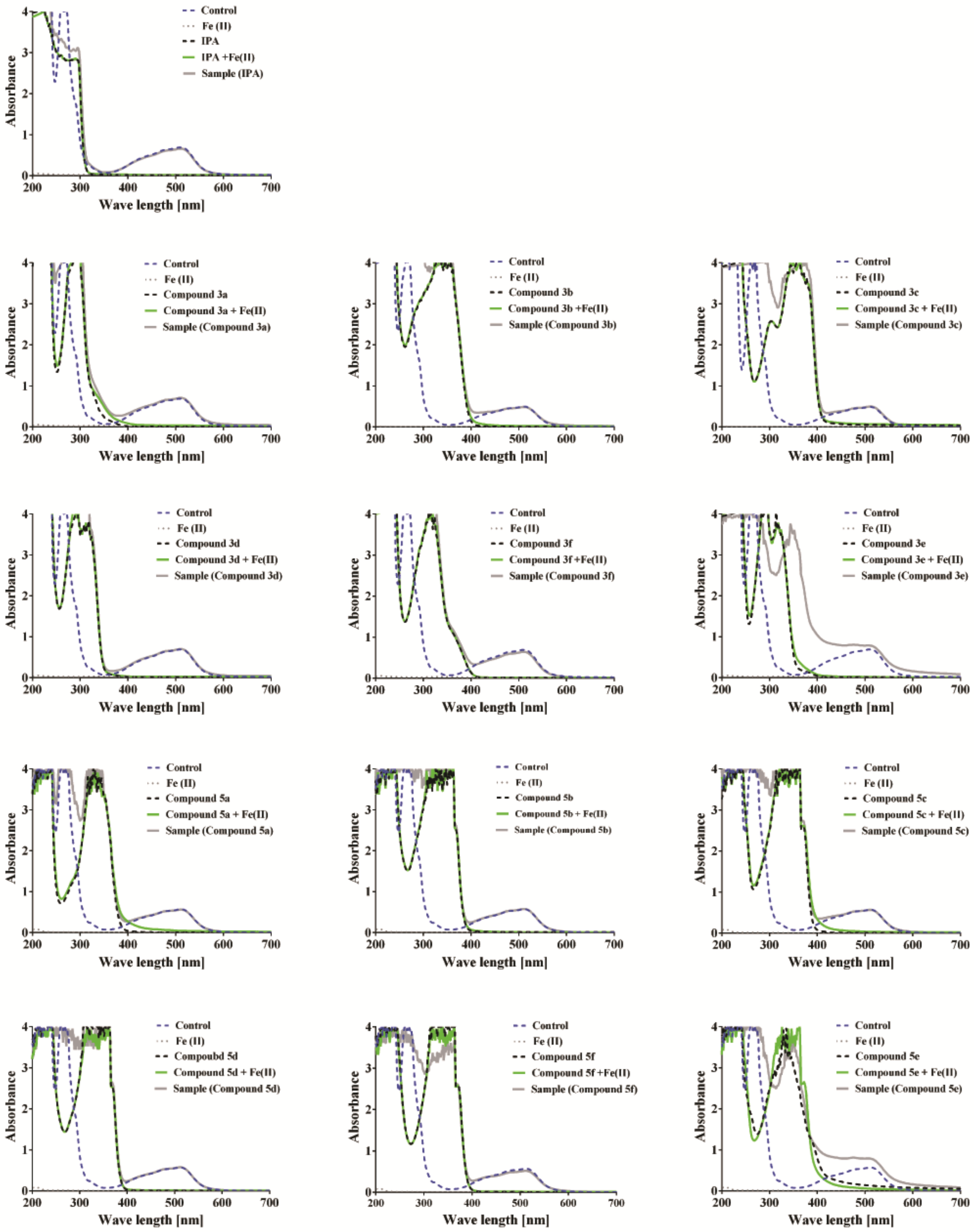

Figure S37. UV-Vis spectra absorbance spectra of the: Control - standard orange-red Fe(II) – 1,10 Phenantroline complex; the tested hydrazone compounds [0.2 mmol/L] alone and in the presence of Fe(II) [0.05 mmol/L]; and the sample solution containing 1,10 phenanthroline [0.2 mmol/L], Fe(II) [0.05 mmol/L] and the hydrazone derivatives [0.2 mmol/L].

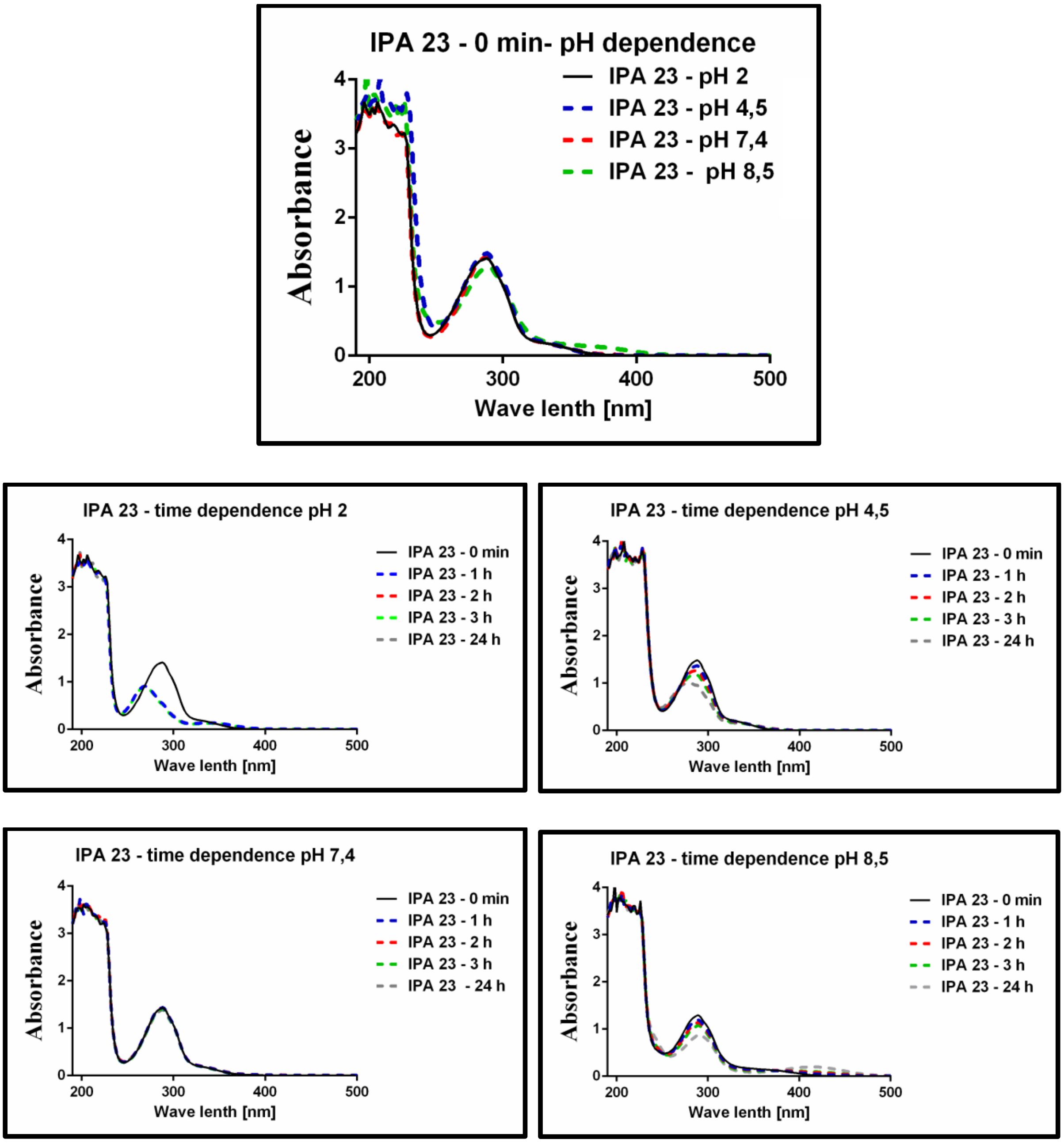

Figure S38. UV spectra of **3a** in buffers with pH 2, 4.5, 7.4 and 8.5 measured at the initial point (0 min) and after 1, 2, 3 and 24 h

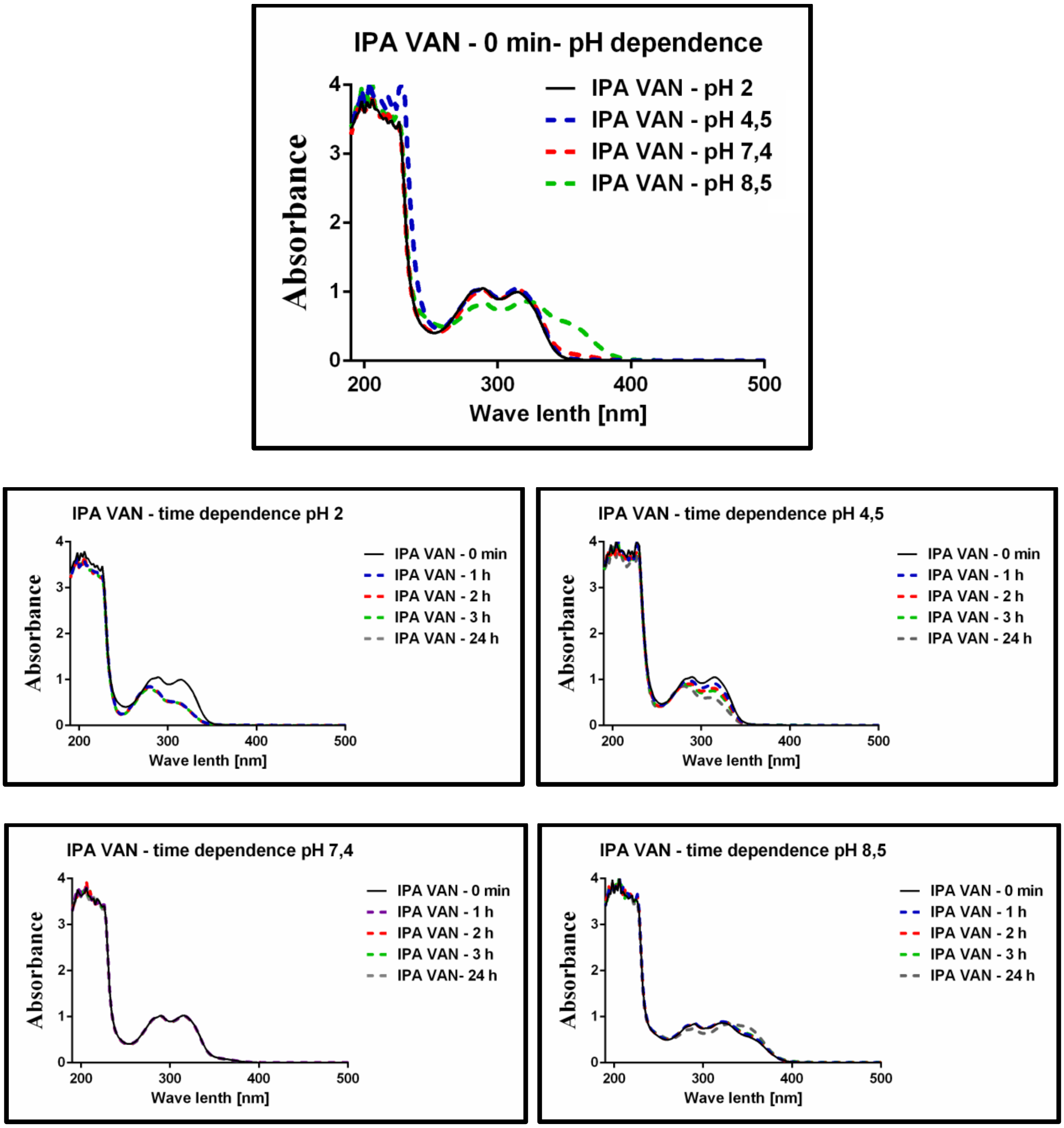

Figure S39. UV spectra of **3d** in buffers with pH 2, 4.5, 7.4 and 8.5 measured at the initial point (0 min) and after 1, 2, 3 and 24 h

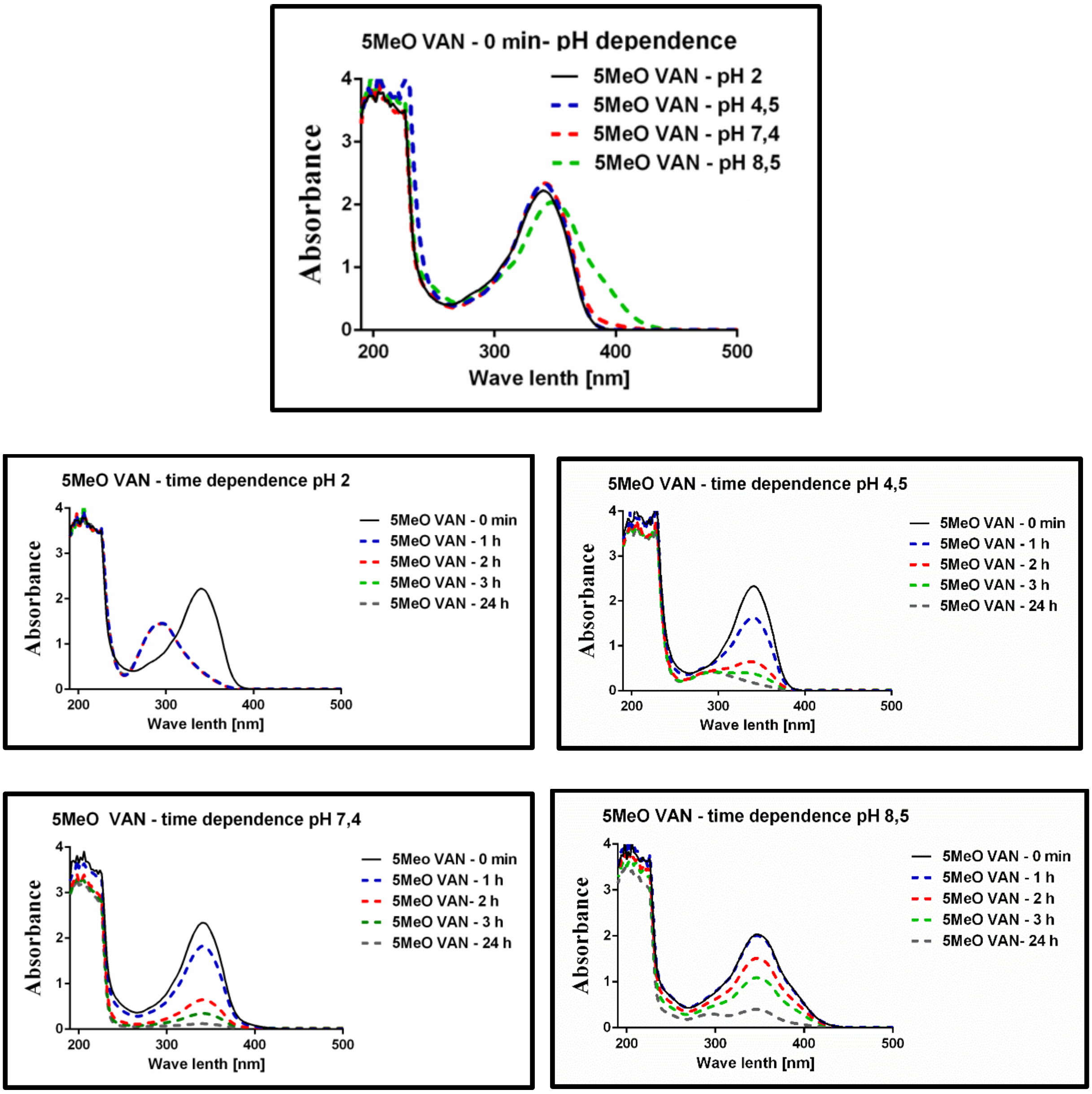

Figure S40. UV spectra of **5d** in buffers with pH 2, 4.5, 7.4 and 8.5 measured at the initial point (0 min) and after 1, 2, 3 and 24 h

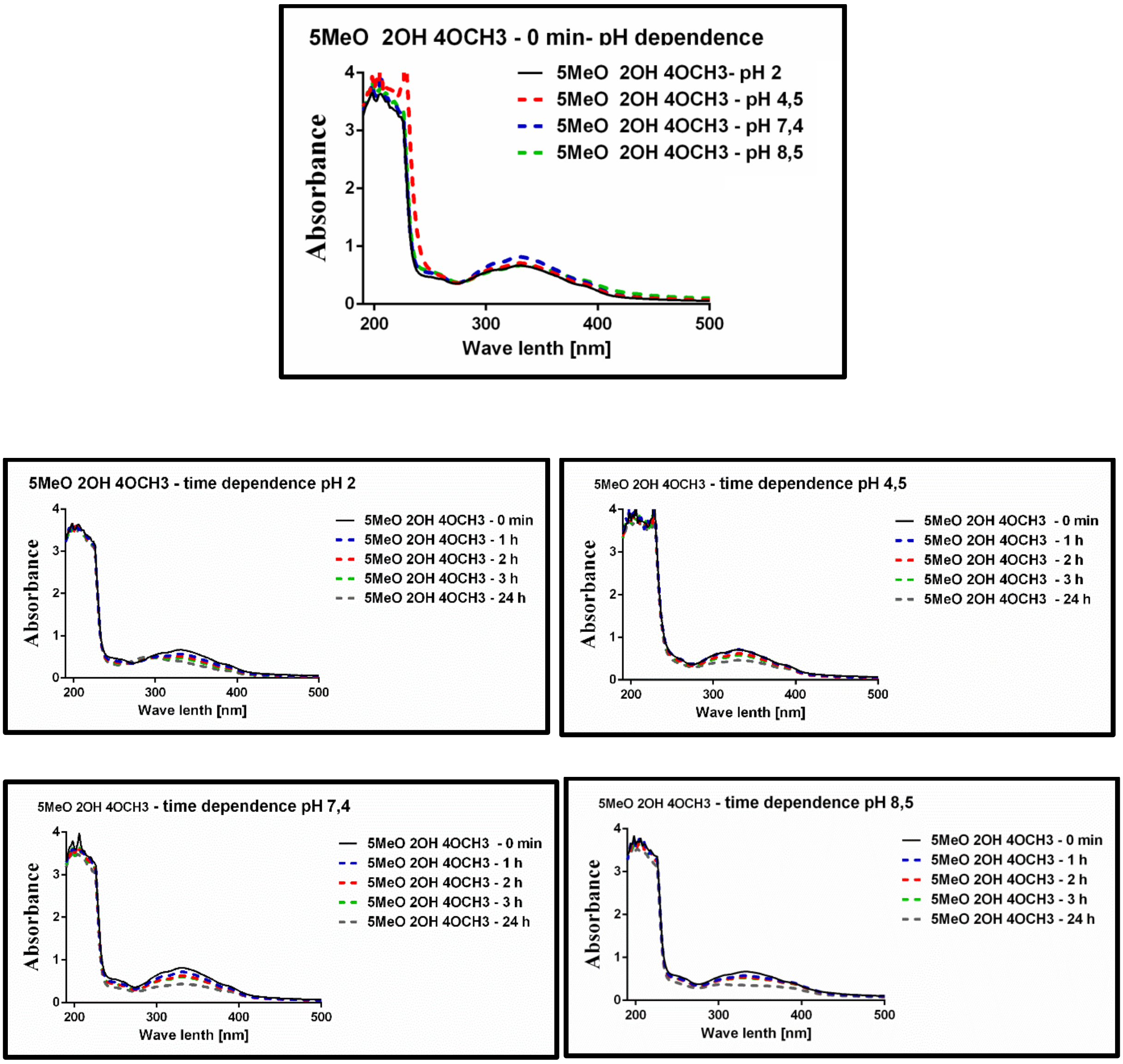

Figure S41. UV spectra of **5e** in buffers with pH 2, 4.5, 7.4 and 8.5 measured at the initial point (0 min) and after 1, 2, 3 and 24 h
